# Benefits and Harms of Sodium-Glucose Co-Transporter 2 Inhibitors in Patients with Type 2 Diabetes: A Systematic Review and Meta-Analysis

**DOI:** 10.1371/journal.pone.0166125

**Published:** 2016-11-11

**Authors:** Heidi Storgaard, Lise L. Gluud, Cathy Bennett, Magnus F. Grøndahl, Mikkel B. Christensen, Filip K. Knop, Tina Vilsbøll

**Affiliations:** 1 Centre for Diabetes Research, Gentofte Hospital, University of Copenhagen, Hellerup, Denmark; 2 Gastrounit, Copenhagen University, Hvidovre Hospital, Hvidovre, Denmark; 3 Centre for Technology Enabled Health Research, Coventry University, Coventry, United Kingdom; 4 Department of Clinical Pharmacology, Bispebjerg Hospital, University of Copenhagen, Copenhagen, Denmark; 5 Department of Clinical Medicine, Faculty of Health and Medical Sciences, University of Copenhagen, Copenhagen, Denmark; 6 The Novo Nordisk Foundation Centre for Basic Metabolic Research, Faculty of Health and Medical Sciences, University of Copenhagen, Copenhagen, Denmark; Florida International University Herbert Wertheim College of Medicine, UNITED STATES

## Abstract

**Objective:**

Sodium-glucose co-transporter 2 inhibitors (SGLT2-i) are a novel drug class for the treatment of diabetes. We aimed at describing the maximal benefits and risks associated with SGLT2-i for patients with type 2 diabetes.

**Design:**

Systematic review and meta-analysis.

**Data Sources and Study Selection:**

We included double-blinded, randomised controlled trials (RCTs) evaluating SGLT2-i administered in the highest approved therapeutic doses (canagliflozin 300 mg/day, dapagliflozin 10 mg/day, and empagliflozin 25 mg/day) for ≥12 weeks. Comparison groups could receive placebo or oral antidiabetic drugs (OAD) including metformin, sulphonylureas (SU), or dipeptidyl peptidase 4 inhibitors (DPP-4-i). Trials were identified through electronic databases and extensive manual searches. Primary outcomes were glycated haemoglobin A1c (HbA1c) levels, serious adverse events, death, severe hypoglycaemia, ketoacidosis and CVD. Secondary outcomes were fasting plasma glucose, body weight, blood pressure, heart rate, lipids, liver function tests, creatinine and adverse events including infections. The quality of the evidence was assessed using GRADE.

**Results:**

Meta-analysis of 34 RCTs with 9,154 patients showed that SGLT2-i reduced HbA1c compared with placebo (mean difference -0.69%, 95% confidence interval -0.75 to -0.62%). We downgraded the evidence to ‘*low quality’* due to variability and evidence of publication bias (P = 0.015). Canagliflozin was associated with the largest reduction in HbA1c (-0.85%, -0.99% to -0.71%). There were no differences between SGLT2-i and placebo for serious adverse events. SGLT2-i increased the risk of urinary and genital tract infections and increased serum creatinine, and exerted beneficial effects on bodyweight, blood pressure, lipids and alanine aminotransferase (*moderate to low quality evidence*). Analysis of 12 RCTs found a beneficial effect of SGLT2-i on HbA1c compared with OAD (-0.20%, -0.28 to -0.13%; *moderate quality evidence*).

**Conclusion:**

This review includes a large number of patients with type 2 diabetes and found that SGLT2-i reduces HbA1c with a notable increased risk in non-serious adverse events. The analyses may overestimate the intervention benefit due bias.

## Introduction

Patients with type 2 diabetes are characterized by hyperglycaemia with elevated levels of glycated haemoglobin A1c (HbA1c) [1] which may lead to microvascular and macrovascular disease [2, 3]. Between 2012 and 2014, three sodium-glucose co-transporter 2 inhibitors (SGLT2-i), canagliflozin, dapagliflozin and empagliflozin, were approved by the US Food and Drug Administration (FDA) [4–6] and the European Medicines Agency (EMA) [7–10] for the treatment of patients with type 2 diabetes. SGLT2-i inhibit glucose reabsorption in the proximal tubules of the kidneys, increasing urinary glucose excretion and reducing the amount of circulating glucose [11]. SGLT2-i have been assessed as monotherapy or combined with other antidiabetic agents including metformin, sulphonylureas (SU), dipeptidyl peptidase 4 inhibitors (DPP-4-i), thiazolidinediones (pioglitazone) or insulin [12–19].

In 2015 the American Diabetes Association (ADA) and the European Association for the Study of Diabetes (EASD) recommend SGLT2-i as second-line agents in the management of type 2 diabetes [20]. A recent randomised controlled trial (RCT) evaluated the effect of empagliflozin on cardiovascular disease (CVD)-associated events in 7,020 patients with type 2 diabetes and a high risk of CVD events [21]. The study found that empagliflozin reduced the relative risk of the CVD events including death from cardiovascular causes, non-fatal myocardial infarction and non-fatal stroke by 14% (absolute risk reduction of 1.6%) compared to placebo. Whether the effect is specific for empagliflozin or represents a class effect for SGLT2-i will be assessed in on-going RCTs assessing the effect of canagliflozin [22]. and dapagliflozin [23] on CVD in patients with type 2 diabetes. However, the efficacy and safety of SGLT2-i in patients with a low to moderate cardiovascular risk or in a real world setting, where patients often have multiple co-morbidities and are treated with several drugs, have not been established.

In contrast to previous meta-analyses evaluating the effects of SGLT2-i in type 2 diabetes, we only included trials, which used the recommended maximum daily doses of the SGLT2-i [24–36] as we expect these dosages to be the most widely used in the clinic [4–10]. Lower or higher doses of SGLT2-i might overestimate or underestimate the efficacy or the risk of adverse events. The present approach provides the evidenced-based clinician with a clear and balanced summary of the existing evidence.

In addition, three studies found that intensive glucose lowering treatments may harm some patients [37–39] and recently, the safety of SGLT2i was put into question by the regulatory agencies [4–6, 8, 9].

We conducted the present systematic review with meta-analyses of RCTs evaluating the safety and efficacy of the SGLT2-i canagliflozin, dapagliflozin, and empagliflozin administered in highest clinically relevant doses for at least 12 weeks compared to placebo or OAD.

## Methods

We conducted our review based on a published protocol (PROSPERO CRD42014008960; S2 File) [40] and adhered to the PRISMA standards [41] for the conduct and reporting of this systematic review and meta-analysis (PRISMA checklist; S3 File).

### Search methods

Electronic searches were performed in the Cochrane Library, MEDLINE, EMBASE, the Science Citation Index and the WHO Trial Search Database, using the following search string: “((Sodium glucose (All Fields) AND co-transporter (All Fields)) OR (2-(3-(4-ethoxybenzyl)-4-chlorophenyl)-6-hydroxymethyltetrahydro-2H-pyran-3,4,5-triol (Supplementary Concept) OR 2-(3-(4-ethoxybenzyl)-4-chlorophenyl)-6-hydroxymethyltetrahydro-2H-pyran-3,4,5-triol (All Fields) OR dapagliflozin (All Fields)) OR (canagliflozin (Supplementary Concept) OR canagliflozin (All Fields)) OR (empagliflozin (Supplementary Concept) OR empagliflozin (All Fields))”. Additional manual searches were performed in reference lists of relevant papers. We obtained additional data on e.g. heart rate, ALT and lipids from the study investigators, the manufacturers and the YODA-project (details listed in S1 File) [42–45]. The last search update was October 2015.

### Trial eligibility and selection

We included English-language, full paper, double-blind RCTs conducted in adult patients (at least 18 years of age) with type 2 diabetes. The interventions assessed were the recommended daily target doses of the SGLT2-i canagliflozin 300 mg; dapagliflozin 10 mg; empagliflozin 25 mg [4–6, 8]. Controls could receive placebo or OAD including metformin, SU or DPP-4-i. We only included RCTs with a treatment duration of at least 12 weeks. Co-interventions (‘add-on’ therapies) with other antidiabetic agents were allowed if administered to both the intervention and control groups. We excluded studies, which involved participants with impaired kidney function and SGLT-2i only approved in Japan (ipragliglozin, luseogliflozin, tofogliflozin) or in clinical development (ertugliflozin, remogliflozin, sotagliflozin).

Trial selection was carried out by two review authors (HS and CB) who independently reviewed the search results and selected trials for inclusion, with involvement of a third review author (CB or TV) if necessary to resolve disagreements. Multiple publications, which reported results from the same RCT, were grouped into ‘studies’ (S1 File).

### Outcome variables and measures

Our primary outcomes were HbA1c (change from baseline) and serious adverse events defined as the number of participants experiencing cancer (all cancers, bladder cancer, breast cancer), death, severe hypoglycaemia, ketoacidosis and CVD. The secondary outcomes were fasting plasma glucose (FPG) (mmol/L), change in body weight (kg), systolic and diastolic blood pressure (SBP and DBP (mmHg)), heart rate (beats per minute (bpm)), plasma lipid profile (low-density lipoprotein (LDL) cholesterol (mmol/L) (which is known to increase the risk of CVD), high-density lipoprotein (HDL) cholesterol (mmol/L) and triglyceride (mmol/L)), alanine amino transferase (U/L), adverse events leading to discontinuation and drug-related adverse events. We also evaluated non-serious adverse events defined as the number of participants experiencing urinary tract infections (UTI), genital tract infections (GTI); 'non-severe' hypoglycaemia, and serum creatinine.

### Data extraction and management

Trial characteristics (methods, participants, interventions, study outcomes, potential risks of bias, and funding source) were recorded. Three authors (HS, MFG and MBC) independently identified outcomes from each included study and extracted outcome data into extraction forms (Excel spreadsheets). Consensus was reached through discussion. For trials presenting data from more than one treatment period (e.g. 26 and 52 weeks), data from the longest treatment period were used. For studies with multiple treatment arms for example SGLT2-i, other OAD and placebo. We conducted separate evaluations and analyses of a) SGLT2-i versus placebo and b) SGLT2-i versus other OAD.

### Assessment of risk of bias and quality

The bias risk assessment followed the Cochrane Collaboration’s risk of bias assessment tool.[46] In each domain, studies were given a rating of low, unclear or high risk. We used the Grades of Recommendation, Assessment, Development and Evaluation (GRADE) system to describe the quality of the evidence and the strength of recommendation, 'high' to 'very low’[47, 48].

### Statistical analyses

We undertook meta-analyses in RevMan [49] using random-effects models, unless stated otherwise. We chose the random-effects model due to an expected heterogeneity. We conducted the analyses with the assumption that if the estimates were similar, then any small-study effects had little effect on the intervention effect estimate. If the random-effects estimates were more beneficial, we planned to re-evaluate whether it was reasonable to conclude that the intervention was more effective in the smaller studies. However, in all of our analyses, the conclusions of the fixed-effect and random-effects meta-analyses were consistent. Based on the expected clinical heterogeneity, we expected that our analyses would display statistical between-trial heterogeneity (I^2^ > 0%). For random-effects models, precision will decrease with increasing heterogeneity and confidence intervals will widen correspondingly. We therefore (*a priori*) planned to report the random-effects model under the assumption that they would provide the most conservative (and a more correct) estimate of the intervention effect. We present results as mean differences (MD) or relative risks (RR) with 95% confidence intervals (CI). For effect sizes of MD, values greater than 0.70 were treated as large; values between 0.40 and 0.70 as moderate; and values less than 0.40 but greater than 0.10 as small.[46] We conducted subgroup analyses on the basis of SGLT2-i type (canagliflozin, dapagliflozin, empagliflozin), and on the basis of the type of OAD (metformin, SU, or DPP-4-i). Differences between subgroups were reported using tests for subgroup differences expressed as P values. I^2^ values were used as a measure of heterogeneity and are reported if they exceeded 30%. For meta-analyses with at least 10 RCTs, publication bias and other small study effects were assessed in regression analyses and funnel plots. For continuous variables, linear regression of the intervention effect estimates on their standard errors, weighting by 1/(variance of the intervention effect estimate), was used (Egger test). For dichotomous outcomes Z/sqrt(V) was regressed against sqrt(V) (Harbord test), where Z is the efficient score and V is Fisher's information (the variance of Z under the null hypothesis).

## Results

### Description of studies

**Fig 1:**
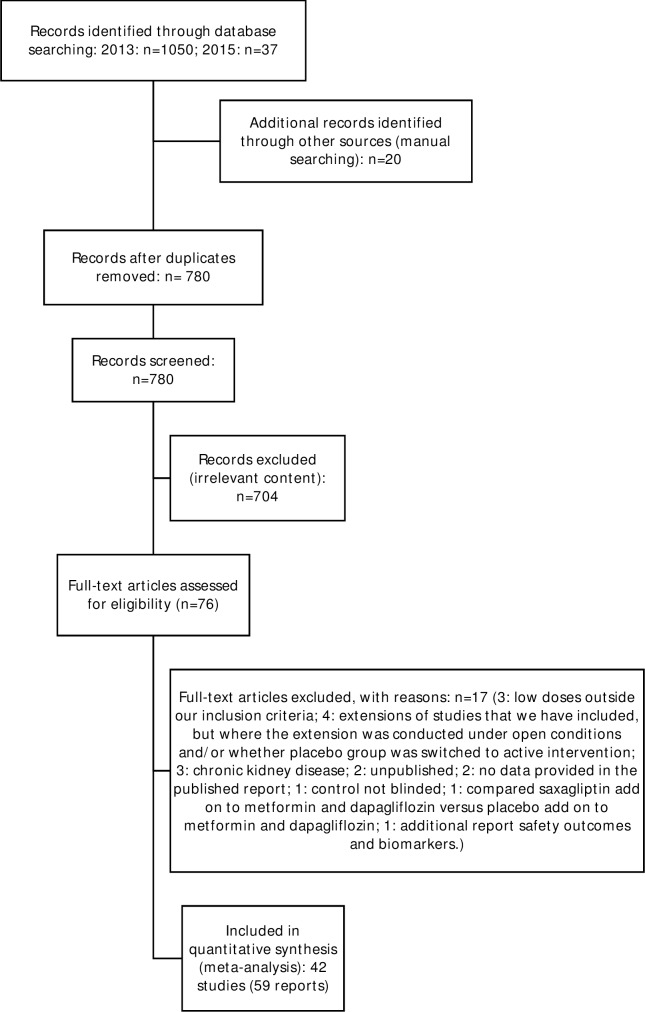
Flowchart for identification and selection of included trials. Preferred Reporting Items for Systematic Reviews and Meta-Analyses (PRISMA) flow chart.

We identified 1,087 potentially eligible records through our searches and included 42 RCTs described in 59 published reports (Fig 1). The total number of participants was 24,500 (S1 File). Thirty-four RCTs compared SGLT2-i versus placebo and 12 compared SGLT2-i versus OAD. Four RCTs were multi-arm, comparing SGLT2-i versus placebo and AD [17, 50–52].

Thirty-four RCTs compared SGLT2-i versus placebo. Seven RCTs evaluated canagliflozin 300 mg,[17, 50, 53–59] 17 evaluated dapagliflozin 10 mg,[12, 18, 19, 51, 60–79] and 10 evaluated empagliflozin 25 mg[13–16, 52, 80–88] (Table 1). Twelve RCTs compared SGLT2-i versus OAD (Table 1). Of these 12 trials, four compared canagliflozin versus glimepiride [89, 90] or sitagliptin[17, 50, 91] and four compared dapagliflozin versus metformin [51, 92], glipizide [93–95] or saxagliptin [96]. The remaining four studies compared empagliflozin versus linagliptin [97, 98], glimepiride [99, 100] or sitagliptin [52]. The maximum doses of metformin were 2000 mg [92] or 1500 mg [51]. The doses of the other OADs was 1 to 8 mg for glimepiride, 20 mg for glipizide, 100 mg for sitagliptin, 5 mg for saxagliptin and 5 mg for linagliptin.

**Table 1:** Characteristics of included randomised controlled trials comparing SGLT2-i versus placebo or other oral antidiabetic drugs (OAD).

Study ID	Intervention	Control	Co-intervention	Number of patients	Duration (weeks)	Age SGLT2-i	Age control	BMI SGLT2-i	BMI control	HbA1c SGLT2-i	HbA1c control
**Placebo controlled RCTs**											
Bode 2013[53, 54]	Canagliflozin 300 mg	Placebo	Pre-existing treatment	714	104*	63.4	63.2	31.5	31.8	7.7	7.8
Forst 2014[55]	Canagliflozin 300 mg	Placebo	Metformin, pioglitazone	344	26	57	58.3	32.8	32.5	7.9	8.0
Gonzalez 2013[50]	Canagliflozin 300 mg	Placebo	Metformin	1,284	26	55.3	55.3	31.4	31.1	7.9	8.0
Inagaki 2013[56]	Canagliflozin 300 mg	Placebo	None	383	12	57.1	57.7	25.9	26.4	8.2	8.0
Rosenstock 2012[17]	Canagliflozin 300 mg	Placebo	Metformin	451	12	52.3	53.3	31.6	30.6	7.7	7.8
Stenløf 2013[57, 58]	Canagliflozin 300 mg	Placebo	none	587	26	55.3	55.7	31.7	31.8	8	8
Wilding 2013[59]	Canagliflozin 300 mg	Placebo	Metformin, SU	469	78*	56.1	56.8	33.2	32.7	8.1	8.1
Bailey 2010[60–62]	Dapagliflozin 10 mg	Placebo	Metformin	546	102*	52.7	53.7	31.2	31.8	7.9	8.1
Bolinder 2012[63, 64, 72]	Dapagliflozin 10 mg	Placebo	Metformin	466	102*	60.6	60.8	32.1	31.7	7.2	7.2
Cefalu 2015[65]	Dapagliflozin 10 mg	Placebo	Insulin, metformin	922	52*	62.8	63	32.6	32.9	8.2	8.1
Ferrannini 2010[12]	Dapagliflozin 10 mg	Placebo	None	485	24	50.6	52.7	33.6	32.3	8.0	7.8
Jabbour 2014[66]	Dapagliflozin 10 mg	Placebo	Metformin, sitagliptin	451	24	54.8	55	-	-	7.9	8.0
Ji 2014[67]	Dapagliflozin 10 mg	Placebo	None	393	24	51.2	49.9	-	-	8.3	8.4
Kaku 2013[68]	Dapagliflozin 10 mg	Placebo	None	279	12	56.5	58.4	-	-	8.2	8.1
Kaku 2014[69]	Dapagliflozin 10 mg	Placebo	Not stated	261	24	57.5	60.4	26.1	25.2	7.5	7.5
Lambers Heerspink 2013[70]	Dapagliflozin 10 mg	Placebo	Metformin, SU	75	12	53.7	58	-	-	7.7	7.5
Leiter 2014[71]	Dapagliflozin 10 mg	Placebo	Pre-existing, treatment	964	52*	63.9	63.6	33	32.7	8.0	8.1
List 2009[51]	Dapagliflozin 10 mg	Placebo	None	389	12	54	53	31	32	8.0	7.9
Mathieu 2015[73]	Dapagliflozin 10 mg	Placebo	Saxagliptin + metformin	320	24	55.2	55	31.2	4.7	8.2	8.2
Matthaei 2015[74, 75]	Dapagliflozin 10 mg	Placebo	Metformin, SU	218	52*	61.1	60.9	31.9	32	8.1	8.2
Rosenstock 2012[76]	Dapagliflozin 10 mg	Placebo	Pioglitazone	420	48**	53.8	53.5	-	-	8.4	8.3
Strojek 2011[77, 78]	Dapagliflozin 10 mg	Placebo	Glimepiride	597	48*	58.9	60.3	-	-	8.1	8.2
Wilding 2009[79]	Dapagliflozin 10 mg	Placebo	Metformin, insulin, pioglitazone, rosiglitazone	71	12	55.7	58.4	35.5	34.8	8.4	8.4
Wilding 2012[18, 19]	Dapagliflozin 10 mg	Placebo	Insulin	108	48*	59.3	58.8	33.4	33.1	8.6	8.5
Ferrannini 2013[80, 81]	Empagliflozin 25 mg	Placebo	None	408	12	57	58	28.3	28.8	7.8	7.8
Häring 2013[13, 14]	Empagliflozin 25 mg	Placebo	Metformin, SU	669	76*	57.4	56.9	28.3	27.9	8.1	8.2
Haring 2014[82, 84]	Empagliflozin 25 mg	Placebo	Metformin	638	76*	55.6	55.5	29.7	28.7	7.9	7.9
Kadowaki 2014[83]	Empagliflozin 25 mg	Placebo	None	547	12	57.3	58.7	25.1	25.6	7.9	7.9
Kovacs 2014[15, 16]	Empagliflozin 25 mg	Placebo	Metformin, pioglitazone	499	76*	54.2	54.6	29.1	29.3	8.1	8.2
Roden 2013[52]	Empagliflozin 25 mg	Placebo	None	899	24	53.8	54.9	28.2	28.7	7.9	7.9
Rosenstock 2013[85]	Empagliflozin 25 mg	Placebo	Metformin	495	12	59	60	31.5	31.3	8.1	8.0
Rosenstock 2014[86]	Empagliflozin 25 mg	Placebo	Insulin +/- metformin	563	52	58	55.3	35	34.7	8.3	8.3
Rosenstock 2015[87]	Empagliflozin 25 mg	Placebo	Insulin +/- metformin and SU	494	78	59.9	58.1	32.7	31.8	8.1	8.3
Ross 2015[88]	Empagliflozin 25 mg	Placebo	Metformin	983	16	58.1	57.9	32.1	32	7.7	7.7
**RCTs with OAD control**											
Cefalu 2013 [89, 90]	Canagliflozin 300 mg	Glimepiride 8 mg	Metformin	1,452	104*	55.8	56.3	31.2	30.9	7.8	7.8
Gonzalez 2013[50]	Canagliflozin 300 mg	Sitagliptin 100 mg	Metformin	1,284	26	55.3	55.5	31.4	32	7.9	7.9
Rosenstock 2012[17]	Canagliflozin 300 mg	Sitagliptin 100mg	Metformin	451	12	52.3	51.7	31.6	31.6	7.7	7.6
Schernthaner 2013[91]	Canagliflozin 300 mg	Sitagliptin 100 mg	Metformin, SU	755	52*	56.6	56.7	31.5	31.7	8.1	8.1
Henry 2012[92]	Dapagliflozin 10 mg	Metformin 1500 mg	None	641	24	51.1	52.7	-	-	9.1	9.1
List 2009[51]	Dapagliflozin 10 mg	Metformin 2000 mg	None	389	12	54	54	31	32	8.0	7.9
Nauck 2011[93–95]	Dapagliflozin 10 mg	Glipizide 20 mg	Metformin	814	208	58	59	31.7	31.2	7.7	7.7
Rosenstock 2015[96]	Dapagliflozin 10 mg	Saxagliptin5 mg	Metformin	534	24	54	55	31.5	31.8	8.9	9.0
DeFronzo 2015[97]	Empagliflozin 25 mg	Linagliptin 5 mg	Metformin	899	52*	55.5	56.2	31.8	30.6	8.0	8.0
Lewin 2015[98]	Empagliflozin 25 mg	Linagliptin5 mg	None	686	52*	56	53.8	31.2	31.9	8.0	8.1
Ridderstråle 2014[99, 100]	Empagliflozin 25 mg	Glimepiride1 to 4 mg	Metformin	1,549	104*	56.2	55.7	30	30.3	7.9	7.9
Roden 2013[52]	Empagliflozin 25 mg	Sitagliptin 100 mg	None	677	24	53.8	55.1	28.2	28.2	7.9	7.9

BMI, body mass index (kg/m^2^); HbA1c, glycated haemoglobin A1c (%); SU, suphonylureas; RCTs, randomised controlled trials.

Thirty-one RCTs were multicentre and multinational carried out in USA, Europe and Asia and three RCTs were conducted Japan [68, 69, 83]. The duration of the RCTs ranged from 12 weeks [17, 51, 56, 68, 70, 79–81, 83, 85] to 102 [53, 54, 57, 60–64, 72, 90, 99, 100], or 104 weeks [53, 54], with the longest duration being 208 weeks [93–95].

### Excluded studies

We excluded 17 RCTs (S1 File) for the following reasons: the dose used in the RCTs did not meet our criteria, open label extension with optional cross-over of placebo, included patients with kidney disease, was not double blind or assessed the combination of SGLT2-i and OAD or insulin. We did not include any abstracts or RCTs published in other languages than English.

### Risk of bias

All RCTs had a low risk of bias in the assessment of randomisation (allocation sequence generation and concealment) and were double blind. One RCT was classified as unclear risk of attrition bias [74]. The published trial report stated that “Approximately 93% of the patients in each treatment arm completed the 24-week double-blind treatment period”. The description of the statistical analyses explained that patients were excluded from the analyses if they did not receive the intervention or did not have follow up assessments. We classified three RCTs as unclear or high risk of reporting bias. One RCT did not provide a clear description of secondary/exploratory outcome measures [51]. The second RCT [70] listed the glomerular filtration rate as the only primary outcome in the registered trial protocol, but in the trial publication, primary outcomes included renal function, blood pressure, and circulating plasma volume. The third RCT did not provide information about adverse events [55]. All RCTs were industry-funded and were classified as unclear risk of bias in the domain ‘other biases’. Accordingly, none of the trials had a low risk of bias in all domains.

### Change in HbA1c

**Fig 2:**
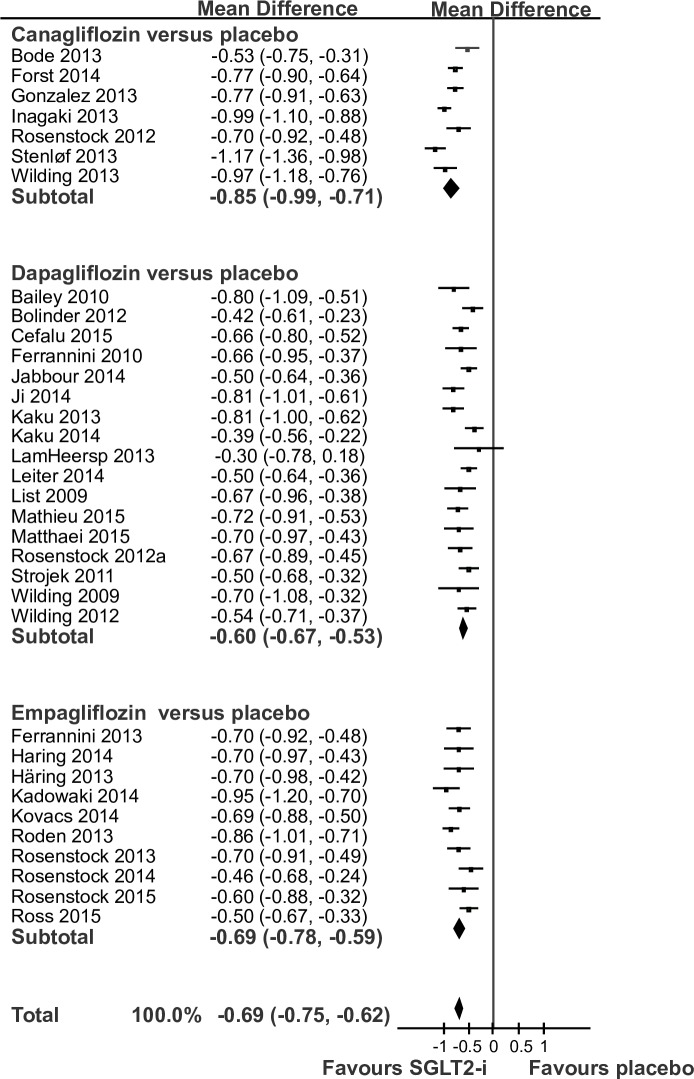
Change in glycated haemoglobin: forest plot of randomized controlled trials comparing sodium-glucose co-transporter 2 inhibitors (SGLT2-i) versus placebo. The plot shows subgroups of trials assessing the different SGLT2-i.

Random-effects meta-analysis of 34 RCTs with 9,154 patients showed that SGLT2-i were associated with a beneficial effect on HbA1c compared with placebo (MD -0.69%, CI -0.75 to -0.62%, Fig 2). Between study heterogeneity was detected (I^2^ = 75%) and we found evidence of small study effects in regression analysis (P = 0.015) and visual inspection of a funnel plot. In addition, subgroup analysis showed a clear difference between subgroups (test for subgroup differences P *=* 0.008). The largest effect size was seen for canagliflozin (-0.85%, -0.99 to -0.71%; Fig 2).

**Fig 3:**
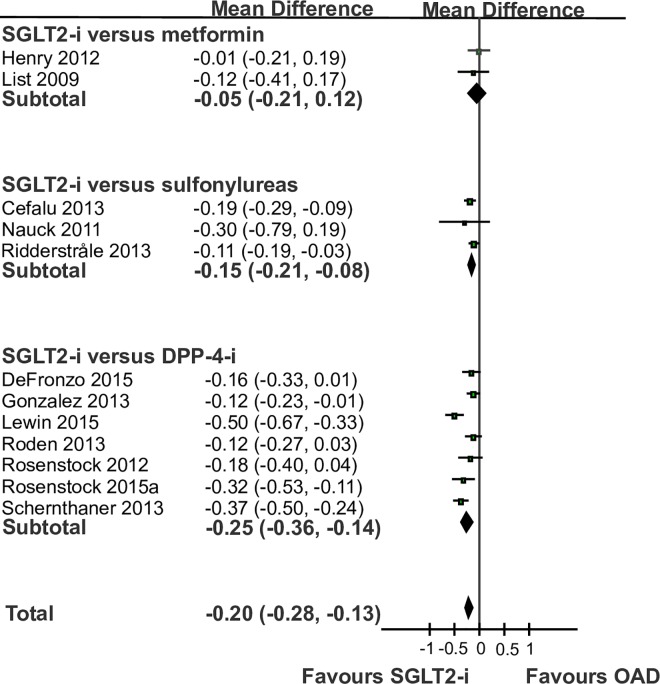
Change in glycated haemoglobin: forest plot of randomized controlled trials comparing sodium-glucose so-transporter 2 inhibitors (SGLT2-i) versus oral antidiabetic drugs (OAD). The plot shows subgroups of trials assessing the different OAD.

Analyses of 12 RCTs showed that SGLT2-i were associated with a larger reduction in HbA1c than OAD (-0.20%, -0.28–0.13%; Fig 3). There was between study heterogeneity, evidence of small study effects (P *=* 0.0385), and no difference between subgroups of trials stratified by the OAD (P *=* 0.11). We found no difference in HbA1c-reduction between SGLT2-i and metformin (-0.05%, 0.21 to 0.12%, Fig 3), but a larger HbA1c reducing effect of SGLT2-i compared with SU (-0.15%, -0.21 to -0.08%) and DPP-4-i (-0.25%, -0.36 to -0.14%).

### Serious adverse events

Only a few serious adverse events were recorded and no differences were seen between SGLT2-i versus placebo (RR 0.99, CI 0.87 to 1.12, 34 RCTs, 10,703 patients) or OAD (1.02, 0.78 to 1.34, 12 RCTs, 6,759 patients). Five patients randomized to SGLT2-i and six patients randomized to placebo reported severe hypoglycaemia (0.75, 0.23 to 2.43, n = 5,077 patients). In trials comparing SGLT2i versus SU, no patients versus three patients experienced a severe hypoglycaemic event (0.13, 0.02 to 0.73, n = 814). No cases of ketoacidosis were reported. In total, 32 of 3,201 patients allocated to SGLT2-i and 29 of 3,223 allocated to placebo developed cancers (1.04, 0.6 to 1.83; 19 RCTs). Only one case of bladder cancer was reported, in the placebo arm of a dapagliflozin study [71]. Six of 2,767 patients were diagnosed with breast cancer in the SGLT2-i arms compared with two of 2,789 patients in the placebo arms (1.73, 0.56 to 5.36; 18 RCTs). When analysing RCTs comparing SGLT2-i with other OAD, seven patients allocated to canagliflozin and three allocated to sitagliptin were diagnosed with other types of cancer than bladder or breast cancer (2.41, 0.69 to 8.37; 2 RCTs). One patient allocated to canagliflozin developed breast cancer [50] and none developed bladder cancer.

CVD events were recorded in 56 of 5,438 patients randomized to SGLT2-i versus 45 of 5,263 randomized to placebo (1.24, 0.86 to 1.81) or OAD (0.78, 0.27 to 2.32).

### Secondary outcomes

#### FPG

As shown in Table 2, analysis of 33 RCTs with 8,914 patients found that FPG levels were 0.9 mmol/L lower in the SGLT2-i arm compared with the placebo arm (-1.0 to -0.8 mmol/L). There was no small study effect (P *=* 0.122) and a difference between subgroups (P *=* 0.04). The largest effect size was seen for canagliflozin (Table 2).

**Table 2:** Number of included patients, mean difference and heterogeneity in meta-analyses of double blind, randomised controlled trials comparing SGLT2-i versus placebo.

SGLT2-i	Total n	Mean difference(confidence interval)	I^2^(Q)%	Subgroup differences
Fasting plasma glucose (mg/dL)	8,914	-28.1 (-31.1; -25.1)	79.1	P = 0.04
Body weight (kg)	9,612	-2.1 (-2.3; -2.0)	44.5	P < 0.01
Systolic blood pressure (mmHg)	9,336	-3.9 (-4.6; -3.3)	33.6	P = 0.03
Diastolic blood pressure (mmHg)	7,402	-2.0 (-2.4; -1.6)	6.3	P = 0.82
Heart rate (bpm)	4,587	-0.6 (-1.3; 0.0)	48.4	P = 0.04
HDL cholesterol (mmol/L)	4,698	0.05 (0.04; 0.07)	31.0	P = 0.03
Triglycerides (mmol/L)	4,704	-0.09 (-0.16; 0.02)	29.8	P < 0.01
LDL cholesterol (mmol/L)	5,431	0.09 (0.04; 0.14)	55.5	P < 0.01
Alanine aminotransferase (U/L)	3,719	-2.8 (-4.0; -1.7)	44.3	P = 0.59
Creatinine (μmol/L)	5,445	0.6 (0.1; 1.1)	11.3	P = 0.05
**Canagliflozin**	**Total n**	**MD (CI)**	**I**^**2**^**(Q)%**	
Fasting plasma glucose (mg/dL)	2,115	-34.0 (-40.4; -27.6)	77	
Body weight (kg)	2,117	-2.6 (-2.9; -2.3)	21	
Systolic blood pressure (mmHg)	2,208	-5.4 (-6.8; -4.0)	42	
Diastolic blood pressure (mmHg)	2,208	-2.1 (-2.8; -1.5)	0	
Heart rate (bpm)	1,336	-1.0 (-1.1; -0.9)	0	
HDL cholesterol (mmol/L)	2,088	0.07 (0.06; 0.09)	0	
Triglyceride (mmol/L)	2,094	-0.21 (-0.30; -0.12)	0	
LDL cholesterol (mmol/L)	2,086	0.19 (0.11; 0.26)	31	
Alanine aminotransferase (U/L)	1,229	-3.5 (-5.8; -1.2)	67	
Creatinine (μmol/L)	1,238	1.8 (0.7; 2.9)	13	
**Dapagliflozin**	**Total n**	**MD (CI)**	**I**^**2**^**(Q)%**	
Fasting plasma glucose (mg/dL)	3,844	-24.6 (-28.7; -20.4)	74	
Body weight (kg)	4,432	-2.0 (-2.2; -1.8)	24	
Systolic blood pressure (mmHg)	3,943	-3.5(-4.3; -2.7)	1	
Diastolic blood pressure (mmHg)	2,009	-2.1 (-2.9; -1.3)	8	
Heart rate (bpm)	2,148	-0.7 (-2.1; 0.7)	63	
HDL cholesterol (mmol/L)	175	0.09 (-0.03; 0.21)	NA	
Triglyceride (mmol/L)	175	0.00 (-0.12; 0.12)	NA	
LDL cholesterol (mmol/L)	175	-0.15 (-0.32; 0.02)	NA	
Alanine aminotransferase (U/L)	1,817	-2.1 (-3.8; -0.5)	30	
Creatinine (μmol/L)	2,335	0.3 (-0.4; 1.0)	0	
**Empagliflozin**	**Total n**	**MD (CI)**	**I**^**2**^**(Q)%**	
Fasting plasma glucose (mg/dL)	2,955	-29.5 (-33.1; -25.9)	60	
Body weight (kg)	3,063	-2.0 (-2.2; -1.7)	9	
Systolic blood pressure (mmHg)	3,185	-3.2 (-4.2; -2.3)	11	
Diastolic blood pressure (mmHg)	3,185	-1.9 (-2.5; -1.2)	31	
Heart rate (bpm)	1,103	0.5 (-0.7; 1.6)	0	
HDL cholesterol (mmol/L)	2,417	0.04 (0.02; 0.06)	27	
Triglyceride (mmol/L)	2,435	0.00 (-0.09; 0.08)	0	
LDL cholesterol (mmol/L)	3,173	0.06 (0.01; 0.10)	0	
Alanine aminotransferase (U/L)	673	-3.4 (-6.1; -0.6)	46	
Creatinine (μmol/L)	1,872	0.3 (-0.6; 1.1)	15	

HDL, high-density lipoprotein; LDL, low-density lipoprotein; SU, sulphonylureas; DPP-4-i, dipeptidyl peptidase 4 inhibitors; The difference between SGLT2-i was assessed using a test for subgroup differences (reported using P-values)

We found no difference between SGLT2-i and metformin [51, 92] or SU [57, 90, 93–95, 99, 100] but a beneficial effect compared with DPP-4-i (-1.0, 1.3 to 0.7 mmol/L, Table 3) [17, 50, 52, 91, 96–98]. The between trial heterogeneity was moderate to high in all analyses.

**Table 3:** Number of included patients, mean difference and heterogeneity in meta-analyses of double blind, randomised controlled trials comparing SGLT2-i versus oral antidiabetic drugs.

**SGLT2-i versus metformin**	**Total n**	**MD (CI)**	**I**^**2**^**(Q)%**
Fasting plasma glucose (mmol/L)	526	-0.3 (-0.5; 0.0)	54.7
Body weight (kg)	530	-1.3 (-1.8; -0.7)	0.0
Systolic blood pressure (mmHg)	467	-3.8 (-6.8; -0.9)	28.5
Diastolic blood pressure (mmHg)	467	-1.9 (-3.3; -0.6)	0.0
Heart rate (bpm)	467	-0.7 (-2.2; 0.8)	0.0
Alanine aminotransferase (U/L)	457	-3.6 (-6.4; -0.7)	0.0
Creatinine (μmol/L)	456	0.3 (-1.5; 2.1)	0.0
**SGLT2-i versus SU**	**Total n**	**MD (CI)**	**I**^**2**^**(Q)%**
Fasting plasma glucose (mmol/L)	2,664	-0.2 (-0.5; 0.1)	93.3
Body weight (kg)	2,811	-4.4 (-4.7; -4.1)	0.0
Systolic blood pressure (mmHg)	2,804	-5.0 (-6.0; -4.0)	18.3
Diastolic blood pressure (mmHg)	2,505	-2.5 (-3.1, -2.0)	0.0
HDL cholesterol (mmol/L)	2,478	0.10 (0.08; 0.12)	0.0
Triglyceride (mmol/L)	2,478	-0.06 (-0.15; 0.02)	0.0
LDL cholesterol (mmol/L)	2,477	0.16 (0.11; 0.21)	0.0
Creatinine (μmol/L)	1,500	-2.0 (-3.1; -0.9)	n/a
**SGLT2-i versus DPP-4-i**	**Total n**	**MD (CI)**	**I**^**2**^**(Q)%**
Fasting plasma glucose (mmol/L)	2,813	-0.6 (-0.7; -0.4)	76.6
Body weight (kg)	2,877	-2.5 (-2.6; -2.3)	0.0
Systolic blood pressure (mmHg)	2,884	-3.8 (-4.8; -2.7)	31.5
Diastolic blood pressure (mmHg)	2,884	-1.8 (-2.4; -1.2)	15.1
Heart rate (bpm)	1,995	-1.5 (-2.6; -0.4)	53.8
HDL cholesterol (mmol/L)	2,039	0.08 (0.06; 0.10)	0.0
Triglyceride (mmol/L)	2,047	-0.06 (-0.20; 0.09)	81.4
LDL cholesterol (mmol/L)	2,483	0.13 (0.07; 0.19)	0.0
Alanine aminotransferase (U/L)	1,571	-3.6 (-6.6; -0.6)	80.4
Creatinine (μmol/L)	2,150	-0.2 (-0.9; 0.6)	0.0

HDL, high-density lipoprotein; LDL, low-density lipoprotein; SU, sulphonylureas; DPP-4-i, dipeptidyl peptidase 4 inhibitors.

#### Bodyweight loss

SGLT2-i were associated with a loss of body weight compared with placebo (-2.1 kg, -2.3 to -2.0 kg). The effect was different in subgroups stratified by the type of SGLT2-i (P < 0.01) with the largest weight reduction associated with canagliflozin (Table 2). SGLT2-i also reduced the body weight compared to OAD (Table 3).

#### Blood pressure and heart rate

SGLT2-i reduced the systolic blood pressure compared with placebo (-3.9 mmHg, -4.6 to -3.3 mmHg), there were subgroup differences (P = 0.03), with the largest effect seen for canagliflozin (Table 2). SGLT2-i also reduced the systolic blood pressure compared with OAD (Table 3). A similar effect was seen in analyses of the diastolic blood pressure (Tables 2 and 3). The heart rate did not differ between patients allocated to SGLT2-i versus placebo (-0.6 bpm, -1.3 to 0.0 bpm) (Table 3). However, there was a difference between subgroups when compared with placebo (P *=* 0.04) and empagliflozin induced a modest increase in heart rate (Table 2). The heart rate in the SGLT2-i group was lower than in the DPP-4-i group (-1.50 bpm, 2.7 to 0.4 bpm).

#### Lipids

SGLT2-i was associated with increased HDL cholesterol compared with placebo (0.05 mmol/L, 0.04 to 0.07 mmol/L). A similar result was achieved for LDL cholesterol (0.09 mmol/L, 0.04 to 0.14 mmol/L), whereas triglyceride decreased (-0.09 mmol/L, -0.16 to -0.02 mmol/L). Subgroup analysis showed a difference between subgroups, with the largest effects seen for canagliflozin on HDL cholesterol, LDL cholesterol and triglycerides (Table 2). SGLT2-i increased HDL and LDL cholesterol, but did not reduce triglycerides compared to OAD (SU and DPP-4-i) (Table 3).

#### Liver function blood tests

Analyses of 18 RCTs with 3,719 patients found evidence that SGLT2-i reduced alanine aminotransferase levels compared with placebo (-2.8 U/L, CI -4.0 to -1.7 U/L) or OAD (Table 3).

#### Serum creatinine

STLG2-i were associated with a 0.60 μmol/L increase in creatinine compared with placebo (0.1 to 1.1 μmol/L) (Table 2). The largest increase was seen for canagliflozin. Analysis of SGLT2-i versus other OAD showed no difference between SGLT2-i and metformin or DPP-4-i (Table 3).

#### Non-serious adverse events

Compared with placebo, SGLT2-i were associated with an increased risk of UTI (1.14, 1.0 to 1.3) and GTI (4.34, 3.35 to 5.63). SGLT2-i were also associated with an increased risk of UTI compared with metformin (2.01, 1.01, 3.98), but not SU (1.05, 0.84 to 1.31) or DPP-4-i (0.89, 0.67 to 1.19). SGLT2-i were associated with an increased risk of GTI compared with metformin (4.48, 1.76 to 11.42), SU (5.41, 3.64 to 8.03) and DPP-4-i (3.69, 2.42 to 5.63; P < 0.00001).

An analysis of 33 RCTs with 10,440 patients found fewer episodes of non-severe hypoglycaemia in the placebo group compared to the SGLT2-i group (1.11, 1.03 to 1.2). Subgrou*p* analysis showed a difference between subgroups (*P =* 0.04). The largest risk of hypoglycaemia was seen for canagliflozin (1.53, 1.15 to 2.03). Dapagliflozin (1.07, 0.95 to 1.19) and empagliflozin (1.03, 0.9 to 1.19) did not increase the risk of non-severe hypoglycaemia. SGLT2-i were associated with a decreased risk of non-severe hypoglycaemia compared with SU (0.16, 0.11, 0.22), but not compared with metformin (0.5, 0.18 to 1.43) or DPP-4-i (1.00, 0.49 to 2.02). In the SGLT2-i group, more participants experienced drug-related adverse effects (1.45, 1.27 to 1.66) and discontinued treatment (1.28, 1.08 to 1.51) compared with placebo.

### Quality of the evidence

We gave evidence from RCT data a high quality rating, but downgraded it if there was unexplained clinically important heterogeneity, the study methodology had a risk of bias, the evidence was indirect, there was important uncertainty around the estimate of effect, or there was evidence for reporting bias. Therefore, it was possible for RCT data to have a very low quality of evidence if several of these concerns were present. Where we downgraded the evidence, it was mainly because there was risk of bias, small study effects, or considerable heterogeneity. Some outcomes had relatively few events (e.g. mortality) and wide CIs (imprecision). The results of many meta-analyses had moderate to high levels of statistical heterogeneity (inconsistency). The heterogeneity between the trials resulted from differences between the three SGLT2-i and in the outcome measures reported, the duration of follow up and the trials inclusion criteria. In the assessment of the primary outcomes, we downgraded the quality of the evidence for glycated haemoglobin in the analyses comparing SGLT2-I by two levels to low quality, due to heterogeneity and evidence of publication bias or other small study effects. We also downgraded the outcome serious adverse events and analyses comparing SGLT2-i versus OAD to moderate quality evidence due to uncertainty (wide confidence intervals) and heterogeneity, respectively.

## Discussion

The highest approved doses of canagliflozin, dapagliflozin and empagliflozin compared with placebo, were effective in reducing HbA1c in patients with type 2 diabetes. In spite of the large number of RCTs with a low risk of bias in several domains, we downgraded the evidence to low quality. Based on our assessment of publication bias and other smalls study effects, we found evidence of bias and therefore a risk that the analyses overestimate the intervention benefit. In the included RCTs, SGLT2-i had no discernible beneficial or harmful effects on serious adverse events including mortality, cancer, ketoacidosis, severe hypoglycaemia, bladder cancer, breast cancer or other cancer types. SGLT2-i also had no effect on CVD events, but SGLT2-i were associated a beneficial effect on CVD-associated risk factors including body weight, blood pressure and lipids (although elevations in LDL lipids may be a concern). As expected, SGLT2-i increased the risk of non-serious adverse events, including serum creatinine levels, UTI and GTI. Additional meta-analyses showed similar effects, when comparing SGLT2-i versus other OAD, but the analyses with active comparators included a smaller number of trials and patients. We also identified important potential limitations, which mainly included a high degree of inconsistency. The inconsistency is likely to reflect clinical heterogeneity in terms of the interventions, populations and follow-up times. Furthermore, selective reporting of outcomes (e.g. CVD, cancer etc.) may also bias the estimates. Therefore, it is possible that the true effect differs somewhat from the estimated effects.

We found statistically clear differences between SGLT2-i in subgroup analyses. The largest effect was seen for canagliflozin in the analyses of HbA1c and CVD-related risk factors. However, none of the trials compared the individual SGLT2-is and the results, therefore, remain exploratory. Thus, the lack of head-to-head comparisons between the SGLT2-i means that we cannot exclude the possibility that the difference between SGLT2-i reflect patient inclusion criteria rather than a true difference between intervention effects.

Patients with type 2 diabetes have a high risk of adverse CVD outcomes [101]. The effects of SGLT2-i on cardiovascular mortality and morbidity in patients with type 2 diabetes are unknown. In one study [21], empagliflozin was associated with a lower rate of cardiovascular events compared with placebo. Despite a sample size of more than 24,500 patients in this review, few RCTs reported CVD as an outcome. In our analyses of CVD events, we found no differences between SGLT2-i and placebo or OAD. We only found beneficial effect on outcomes that may be associated with a lower risk of CVD.

We found a beneficial effect of SGLT2-i on alanine aminotransferase, which is associated with non-alcoholic fatty liver disease in the early phase. Increasing evidence suggests that non-alcoholic fatty liver disease may increase the risk of CVD [102–104]. SGLT2-i decreased alanine aminotransferase both in comparison to placebo and OAD. While such improvements may be attributed solely to weight loss, rather than drug-specific effects [105] additional evidence is needed to determine the potential clinical implications of the findings.

We included creatinine, which may reflect dehydration due to the glycosuria. On SGLT2-i, approximately 500 ml of water after treatment is initiated [106]. The loss generally decreases during long term treatment. Increased serum creatinine may although reflect a worsening of kidney function which is predictive of CVD [107–109]. The largest increase in creatinine levels was found in RCTs evaluating canagliflozin. Whether this translates to an increased risk of CVD events in patients taking SGLT2-i over the long-term is unclear.

Recently, ketoacidosis has been reported as an adverse effect of SGLT2-i [110]. The RCTs in this review did not routinely report ketoacidosis as an outcome. Theoretically, there is a potential for developing ketoacidosis as a result of the insulin-independent glucose excretion combined with increased glucagon levels [111]. However, a recent large RCT [21] has found a low incidence of ketoacidosis (≤ 0.1%) and that the risk was similar in patients treated with empagliflozin and placebo.

SGLT2-i are widely studied and several reviews and meta-analyses have recently been published [34–36]. Compared to these studies our systematic review with meta-analysis has distinct differences in the dosages and outcomes that we address. Zaccardi et al. performed a network meta-analysis that focused on efficacy and safety of SGLT2-i [34]. In contrast to our meta-analysis, they included trials with several different doses of canagliflozin, dapagliflozin and empagliflozin and they reported fewer secondary outcomes than us (we also include e.g. ALT, Creatinine and heart rate). In another meta-analysis, Wu et al. examined the effects of SGLT2-i on cardiovascular events, death and major safety outcomes in adults with type 2 diabetes [35]. No beneficial effects of SGLT2-i were reported. We analysed both efficacy and safety data. In the network meta-analysis by Shyangdan et al., the primary aim was to compare the efficacy of SGLT2-i [36]. The investigators only included trials on SGLT2-i in monotherapy or as add on to metformin in patients with type 2 diabetes. Only a total of 10 trials were included and no data on adverse events were provided.

Future RCTs would ideally be long-lasting and large-scale comparing SGLT2-i with placebo or existing therapies. Such RCTs should additionally include reporting of serious adverse events such as CVD risk, ketoacidosis and severe hypoglycaemia, and monitoring of renal safety, with adequate follow-up (over one year), to establish the long-term consequences of SGLT2-i therapy.

## Conclusion

Based on our review we found evidence that clinically relevant doses i.e. the recommended daily target doses of SGLT2-i that are included in this review, during more than 12 weeks reduce HbA1c levels in patients with type 2 diabetes compared with placebo and other existing oral therapies. We planned to include high-quality RCTs with clinically relevant doses and sufficient follow up to generate an estimate based on the best available evidence. However, our analyses showed evidence of bias and heterogeneity. Likewise, the incidence of serious adverse events including mortality, CVD and cancer was not increased as a result of SGLT2-i, but reporting was inconsistent. Several CVD risk factors such as obesity, blood pressure and HDL cholesterol may be improved by SGLT2-i therapy, whereas the incidences of UTI and GTI are increased in the SGLT2-i groups. Additional evidence may therefore be needed to determine the benefit and safely of SGLT2-i. The RCTs included in our review were largely carried out in research hospital settings. Given the high prevalence of type 2 diabetes in the general population, RCTs conducted outside the hospital settings seem warranted.

## Supporting Information

S1 Fig Risk of bias across all studies. Low risk of bias: ‘+’ in green circle; unclear risk of bias ‘?’ in yellow circle; no studies were at high risk of bias in any domain.
(TIF)

S2 Fig Risk of bias summary graph. (TIF)

S1 File Data sources. Multiple publications which reported the same RCT were grouped into ‘studies’.
(PDF)

S2 File Study protocol PROSPERO CRD42014008960. (PDF)

S3 File PRISMA checklist. (PDF)

S1 Table Characteristics of included studies and risk of bias assessments. (PDF)

S2 Table Primary outcome effect sizes, all comparisons. (PDF)

S3 Table Characteristics of excluded studies. (PDF)

## References

[1] Kahn SE, Cooper ME, Del Prato S. Pathophysiology and treatment of type 2 diabetes: perspectives on the past, present, and future. Lancet. 2014;383(9922):1068–83. 10.1016/S0140-6736(13)62154-6 24315620 PMC4226760

[2] Gaede P, Vedel P, Larsen N, Jensen GVH, Parving HH, Pedersen O. Multifactorial intervention and cardiovascular disease in patients with type 2 diabetes. N Engl J Med. 2003;348:383–93. 10.1056/NEJMoa021778 12556541

[3] Stratton IM, Adler AI, Neil HA, Matthews DR, Manley SE, Cull CA, et al Association of glycaemia with macrovascular and microvascular complications of type 2 diabetes (UKPDS 35): prospective observational study. BMJ. 2000;321(7258):405–12. 10938048 10.1136/bmj.321.7258.405 PMC27454

[4] FDA. Briefing document NDA 204042 Invokana (canagliflozin) tablets. 2013.

[5] FDA. Briefing document NDA 202293: Dapagliflozin oral tablets, 5 and 10mg. 2013.

[6] FDA. Jardiance (empagliflozin) press release. 2014.

[7] EMA. Assessment report, canagliflozin EMA/718531/2013. 2013.

[8] EMA. Jardiance (empagliflozin): assessment report; procedure No. EMEA/H/C/002677/0000. 2014.

[9] EMA. Jardiance (empagliflozin): procedure No. EMEA/H/C/002677/0000 (Annex 1). 2014.

[10] EMA. European Medicines Agency Assessment Report: Dapagliflozin (Forxiga). 2015.

[11] Ferrannini E, Solini A. SGLT2 inhibition in diabetes mellitus: rationale and clinical prospects. Nat Rev Endocrinol. 2012;8(8):495–502. 10.1038/nrendo.2011.243 22310849

[12] Ferrannini E, Ramos SJ, Salsali A, Tang W, List JF. Dapagliflozin monotherapy in type 2 diabetic patients with inadequate glycemic control by diet and exercise: a randomized, double-blind, placebo-controlled, phase 3 trial. Diabetes Care. 2010;33(10):2217–24. 10.2337/dc10-0612 20566676 PMC2945163

[13] Haring HU, Merker L, Seewaldt-Becker E, Weimer M, Meinicke T, Woerle HJ, et al Empagliflozin as add-on to metformin plus sulfonylurea in patients with type 2 diabetes: a 24-week, randomized, double-blind, placebo-controlled trial. Diabetes Care. 2013;36(11):3396–404. 10.2337/dc12-2673 23963895 PMC3816918

[14] Haering HU, Merker L, Christiansen AV, Roux F, Salsali A, Kim G, et al Empagliflozin as add-on to metformin plus sulphonylurea in patients with type 2 diabetes. Diabetes Res Clin Pract. 2015;110(1):82–90. 10.1016/j.diabres.2015.05.044 26324220

[15] Kovacs CS, Seshiah V, Merker L, Christiansen AV, Roux F, Salsali A, et al Empagliflozin as add-on therapy to pioglitazone with or without metformin in patients with type 2 diabetes mellitus. Clin Ther. 2015;37(8):1773–88 e1. 10.1016/j.clinthera.2015.05.511 26138864

[16] Kovacs CS, Seshiah V, Swallow R, Jones R, Rattunde H, Woerle HJ, et al Empagliflozin improves glycaemic and weight control as add-on therapy to pioglitazone or pioglitazone plus metformin in patients with type 2 diabetes: a 24-week, randomized, placebo-controlled trial. Diabetes Obes Metab. 2014;16(2):147–58. 10.1111/dom.12188 23906415

[17] Rosenstock J, Aggarwal N, Polidori D, Zhao Y, Arbit D, Usiskin K, et al Dose-ranging effects of canagliflozin, a sodium-glucose cotransporter 2 inhibitor, as add-on to metformin in subjects with type 2 diabetes. Diabetes Care. 2012;35(6):1232–8. 10.2337/dc11-1926 22492586 PMC3357223

[18] Wilding JP, Woo V, Rohwedder K, Sugg J, Parikh S, Dapagliflozin 006 Study G. Dapagliflozin in patients with type 2 diabetes receiving high doses of insulin: efficacy and safety over 2 years. Diabetes Obes Metab. 2014;16(2):124–36. 10.1111/dom.12187 23911013

[19] Wilding JP, Woo V, Soler NG, Pahor A, Sugg J, Rohwedder K, et al Long-term efficacy of dapagliflozin in patients with type 2 diabetes mellitus receiving high doses of insulin: a randomized trial. Ann Intern Med. 2012;156(6):405–15. 10.7326/0003-4819-156-6-201203200-00003 22431673

[20] Inzucchi SE, Bergenstal RM, Buse JB, Diamant M, Ferrannini E, Nauck M, et al Management of hyperglycemia in type 2 diabetes, 2015: a patient-centered approach: update to a position statement of the American Diabetes Association and the European Association for the Study of Diabetes. Diabetes Care. 2015;38(1):140–9. 10.2337/dc14-2441 25538310

[21] Zinman B, Wanner C, Lachin JM, Fitchett D, Bluhmki E, Hantel S, et al Empagliflozin, cardiovascular outcomes, and mortality in type 2 diabetes. N Engl J Med. 2015;373(22):2117–28. 10.1056/NEJMoa1504720 26378978

[22] Neal B, Perkovic V, de Zeeuw D, Mahaffey KW, Fulcher G, Stein P, et al Rationale, design, and baseline characteristics of the Canagliflozin Cardiovascular Assessment Study (CANVAS)—a randomized placebo-controlled trial. Am Heart J. 2013;166(2):217–23 e11. 10.1016/j.ahj.2013.05.007 23895803

[23] Raz I, Wiviott S. Multicenter trial to evaluate the effect of dapagliflozin on the incidence of cardiovascular events (DECLARE-TIMI58): NCT01730534: AstraZeneca. 2016.

[24] Baker WL, Smyth LR, Riche DM, Bourret EM, Chamberlin KW, White WB. Effects of sodium-glucose co-transporter 2 inhibitors on blood pressure: a systematic review and meta-analysis. J Am Soc Hypertens. 2014;8(4):262–75 e9. 10.1016/j.jash.2014.01.007 24602971

[25] Berhan A, Barker A. Sodium glucose co-transport 2 inhibitors in the treatment of type 2 diabetes mellitus: a meta-analysis of randomized double-blind controlled trials. BMC Endocr Disord. 2013;13(1):58 10.1186/1472-6823-13-58 24341330 PMC3883465

[26] Goring S, Hawkins N, Wygant G, Roudaut M, Townsend R, Wood I, et al Dapagliflozin compared with other oral anti-diabetes treatments when added to metformin monotherapy: a systematic review and network meta-analysis. Diabetes Obes Metab. 2014;16(5):433–42. 10.1111/dom.12239 24237939

[27] Liakos A, Karagiannis T, Athanasiadou E, Sarigianni M, Mainou M, Papatheodorou K, et al Efficacy and safety of empagliflozin for type 2 diabetes: a systematic review and meta-analysis. Diabetes Obes Metab. 2014;16(10):984–93. 10.1111/dom.12307 24766495

[28] Musso G, Gambino R, Cassader M, Pagano G. A novel approach to control hyperglycemia in type 2 diabetes: sodium glucose co-transport (SGLT) inhibitors: systematic review and meta-analysis of randomized trials. Ann Med. 2012;44(4):375–93. 10.3109/07853890.2011.560181 21495788

[29] Singh AK, Singh R. Combination therapy of sodium-glucose co-transporter-2 inhibitors and dipeptidyl peptidase-4 inhibitors in type 2 diabetes: rationale and evidences. Expert Rev Clin Pharmacol. 2016;9(2):229–40. 10.1586/17512433.2016.1123616 26589238

[30] Sonesson C, Johansson PA, Johnsson E, Gause-Nilsson I. Cardiovascular effects of dapagliflozin in patients with type 2 diabetes and different risk categories: a meta-analysis. Cardiovasc Diabetol. 2016;15(1):37 10.1186/s12933-016-0356-y 26895767 PMC4761166

[31] Triplitt C, Solis-Herrera C, Cersosimo E, Abdul-Ghani M, Defronzo RA. Empagliflozin and linagliptin combination therapy for treatment of patients with type 2 diabetes mellitus. Expert Opin Pharmacother. 2015;16(18):2819–33. 10.1517/14656566.2015.1114098 26583910

[32] Vasilakou D, Karagiannis T, Athanasiadou E, Mainou M, Liakos A, Bekiari E, et al Sodium-glucose cotransporter 2 inhibitors for type 2 diabetes: a systematic review and meta-analysis. Ann Intern Med. 2013;159(4):262–74. 10.7326/0003-4819-159-4-201308200-00007 24026259

[33] Yoon J, Min SH, Hahn S, Cho YM. Indirect Comparison to Evaluate the Efficacy and Safety of Dipeptidyl Peptidase-4 Inhibitors (DPP4I) and Sodium-Glucose Cotransporter 2 Inhibitors (SGLT2I) Added to Insulin Therapy in Type 2 Diabetes. Value Health. 2015;18(7):A599 10.1016/j.jval.2015.09.2050 26533362

[34] Zaccardi F, Webb DR, Htike ZZ, Youssef D, Khunti K, Davies MJ. Efficacy and safety of sodium-glucose co-transporter-2 inhibitors in type 2 diabetes mellitus: systematic review and network meta-analysis. Diabetes Obes Metab. 2016;18(8):783–94. 10.1111/dom.12670 27059700

[35] Wu JH, Foote C, Blomster J, Toyama T, Perkovic V, Sundstrom J, et al Effects of sodium-glucose cotransporter-2 inhibitors on cardiovascular events, death, and major safety outcomes in adults with type 2 diabetes: a systematic review and meta-analysis. Lancet Diabetes Endocrinol. 2016;4(5):411–9. 10.1016/S2213-8587(16)00052-8 27009625

[36] Shyangdan DS, Uthman OA, Waugh N. SGLT-2 receptor inhibitors for treating patients with type 2 diabetes mellitus: a systematic review and network meta-analysis. BMJ Open. 2016;6(2). 10.1136/bmjopen-2015-009417 26911584 PMC4769433

[37] Holman RR, Paul SK, Bethel MA, Matthews DR, Neil HA. 10-year follow-up of intensive glucose control in type 2 diabetes. N Engl J Med. 2008;359(15):1577–89. 10.1056/NEJMoa0806470 18784090

[38] Duckworth W, Abraira C, Moritz T, Reda D, Emanuele N, Reaven PD, et al Glucose control and vascular complications in veterans with type 2 diabetes. N Engl J Med. 2009;360(2):129–39. 10.1056/NEJMoa0808431 19092145

[39] Patel A, MacMahon S, Chalmers J, Neal B, Billot L, Woodward M, et al Intensive blood glucose control and vascular outcomes in patients with type 2 diabetes. N Engl J Med. 2008;358(24):2560–72. 10.1056/NEJMoa0802987 18539916

[40] Storgaard H, Gluud LL, Christensen M, Knop FK, Vilsboll T. The effects of sodium-glucose co-transporter 2 inhibitors in patients with type 2 diabetes: protocol for a systematic review with meta-analysis of randomised trials. BMJ Open. 2014;4(8):e005378 10.1136/bmjopen-2014-005378 25232561 PMC4139650

[41] Moher D, Liberati A, Tetzlaff J, Altman DG, Group P. Preferred reporting items for systematic reviews and meta-analyses: the PRISMA statement. BMJ. 2009;339. PMC3090117 21603045

[42] Childers K. Additional data: HDL and triglyceride absolute changes of ALAT, LDL, and serum creatinine. Overall adverse events (all types) tables by System Organ Class (SOC), other adverse events including fractures: Janssen Pharmaceuticals Inc, a pharmaceutical company of Johnson & Johnson. 2014.

[43] Lund S. Additional data: LDL, ALAT, serum creatinine, heart rate, systolic and diastolic blood pressure. Adverse events fractures, all cancers, bladder and breast cancer: Boehringer Ingelheim Pharma GmbH & Co. 2014.

[44] Nahrebne K. Additional data CSRs: LDL given in percentage change from baseline, ALAT, serum creatinine, heart rate, systolic and diastolic blood pressure. Overall adverse events (all types) tables by System Organ Class (SOC), other adverse events including fractures: AstraZeneca Pharmaceuticals. 2014.

[45] The YODA-project, Yale University (http://yoda.yale.edu/) [Internet]. 2014.

[46] Higgins JPT, Green S. Cochrane Handbook for Systematic Reviews of Interventions Version 5.1.0 [updated March 2011]. The Cochrane Collaboration, 2011.

[47] Guyatt G, Oxman AD, Akl EA, Kunz R, Vist G, Brozek J, et al GRADE guidelines: 1. Introduction-GRADE evidence profiles and summary of findings tables. J Clin Epidemiol. 2011;64(4):383–94. 10.1016/j.jclinepi.2010.04.026 21195583

[48] Schünemann H, Brozek J, Guyatt G, Oxman A. GRADE handbook for grading quality of evidence and strength of recommendations. Updated October 2013. The GRADE Working Group, 2013.

[49] Review Manager (RevMan) [Computer program]. 5.3.5 ed. Copenhagen: The Nordic Cochrane Centre The Cochrane Collaboration; 2014.

[50] Lavalle-Gonzalez FJ, Januszewicz A, Davidson J, Tong C, Qiu R, Canovatchel W, et al Efficacy and safety of canagliflozin compared with placebo and sitagliptin in patients with type 2 diabetes on background metformin monotherapy: a randomised trial. Diabetologia. 2013;56(12):2582–92. 10.1007/s00125-013-3039-1 24026211 PMC3825495

[51] List JF, Woo V, Morales E, Tang W, Fiedorek FT. Sodium-glucose cotransport inhibition with dapagliflozin in type 2 diabetes. Diabetes Care. 2009;32(4):650–7. 10.2337/dc08-1863 19114612 PMC2660449

[52] Roden M, Weng J, Eilbracht J, Delafont B, Kim G, Woerle HJ, et al Empagliflozin monotherapy with sitagliptin as an active comparator in patients with type 2 diabetes: a randomised, double-blind, placebo-controlled, phase 3 trial. Lancet Diabetes Endocrinol. 2013;1(3):208–19. 10.1016/s2213-8587(13)70084-6 24622369

[53] Bode B, Stenlof K, Sullivan D, Fung A, Usiskin K. Efficacy and safety of canagliflozin treatment in older subjects with type 2 diabetes mellitus: a randomized trial. Hosp Pract (1995). 2013;41(2):72–84. 10.3810/hp.2013.04.1020 23680739

[54] Bode B, Stenlof K, Harris S, Sullivan D, Fung A, Usiskin K, et al Long-term efficacy and safety of canagliflozin over 104 weeks in patients aged 55–80 years with type 2 diabetes. Diabetes Obes Metab. 2015;17(3):294–303. 10.1111/dom.12428 25495720

[55] Forst T, Guthrie R, Goldenberg R, Yee J, Vijapurkar U, Meininger G, et al Efficacy and safety of canagliflozin over 52 weeks in patients with type 2 diabetes on background metformin and pioglitazone. Diabetes Obes Metab. 2014;16(5):467–77. 10.1111/dom.12273 24528605 PMC4237547

[56] Inagaki N, Kondo K, Yoshinari T, Maruyama N, Susuta Y, Kuki H. Efficacy and safety of canagliflozin in Japanese patients with type 2 diabetes: a randomized, double-blind, placebo-controlled, 12-week study. Diabetes Obes Metab. 2013;15(12):1136–45. 10.1111/dom.12149 23782594 PMC3906835

[57] Stenlof K, Cefalu WT, Kim KA, Alba M, Usiskin K, Tong C, et al Efficacy and safety of canagliflozin monotherapy in subjects with type 2 diabetes mellitus inadequately controlled with diet and exercise. Diabetes Obes Metab. 2013;15(4):372–82. 10.1111/dom.12054 23279307 PMC3593184

[58] Stenlof K, Cefalu WT, Kim KA, Jodar E, Alba M, Edwards R, et al Long-term efficacy and safety of canagliflozin monotherapy in patients with type 2 diabetes inadequately controlled with diet and exercise: findings from the 52-week CANTATA-M study. Curr Med Res Opin. 2014;30(2):163–75. 10.1185/03007995.2013.850066 24073995

[59] Wilding JP, Charpentier G, Hollander P, Gonzalez-Galvez G, Mathieu C, Vercruysse F, et al Efficacy and safety of canagliflozin in patients with type 2 diabetes mellitus inadequately controlled with metformin and sulphonylurea: a randomised trial. Int J Clin Pract. 2013;67(12):1267–82. 10.1111/ijcp.12322 24118688 PMC4282288

[60] Bailey CJ, Gross JL, Pieters A, Bastien A, List JF. Effect of dapagliflozin in patients with type 2 diabetes who have inadequate glycaemic control with metformin: a randomised, double-blind, placebo-controlled trial. Lancet. 2010;375(9733):2223–33. 10.1016/s0140-6736(10)60407-2 20609968

[61] Bailey CJ, Gross JL, Hennicken D, Iqbal N, Mansfield TA, List JF. Dapagliflozin add-on to metformin in type 2 diabetes inadequately controlled with metformin: a randomized, double-blind, placebo-controlled 102-week trial. BMC Med. 2013;11(1):43 10.1186/1741-7015-11-43 23425012 PMC3606470

[62] Bailey CJ, Gross JL, Hennicken D, Iqbal N, Mansfield TA, List JF. Correction: Dapagliflozin add-on to metformin in type 2 diabetes inadequately controlled with metformin: a randomized, double-blind, placebo-controlled 102-week trial. BMC Med. 2013;11:193-. 10.1186/1741-7015-11-43 PMC3606470 23425012

[63] Bolinder J, Ljunggren O, Kullberg J, Johansson L, Wilding J, Langkilde AM, et al Effects of dapagliflozin on body weight, total fat mass, and regional adipose tissue distribution in patients with type 2 diabetes mellitus with inadequate glycemic control on metformin. J Clin Endocrinol Metab. 2012;97(3):1020–31. 10.1210/jc.2011-2260 22238392

[64] Bolinder J, Ljunggren O, Johansson L, Wilding J, Langkilde AM, Sjostrom CD, et al Dapagliflozin maintains glycaemic control while reducing weight and body fat mass over 2 years in patients with type 2 diabetes mellitus inadequately controlled on metformin. Diabetes Obes Metab. 2014;16(2):159–69. 10.1111/dom.12189 23906445

[65] Cefalu WT, Leiter LA, de Bruin TW, Gause-Nilsson I, Sugg J, Parikh SJ. Dapagliflozin's Effects on Glycemia and Cardiovascular Risk Factors in High-Risk Patients With Type 2 Diabetes: A 24-Week, Multicenter, Randomized, Double-Blind, Placebo-Controlled Study With a 28-Week Extension. Diabetes Care. 2015;38(7):1218–27. 10.2337/dc14-0315 25852208 PMC4831907

[66] Jabbour SA, Hardy E, Sugg J, Parikh S, Study G. Dapagliflozin is effective as add-on therapy to sitagliptin with or without metformin: a 24-week, multicenter, randomized, double-blind, placebo-controlled study. Diabetes Care. 2014;37(3):740–50. 10.2337/dc13-0467 24144654

[67] Ji L, Ma J, Li H, Mansfield TA, T'Joen C L, Iqbal N, et al Dapagliflozin as monotherapy in drug-naive Asian patients with type 2 diabetes mellitus: a randomized, blinded, prospective phase III study. Clin Ther. 2014;36(1):84–100 e9. 10.1016/j.clinthera.2013.11.002 24378206

[68] Kaku K, Inoue S, Matsuoka O, Kiyosue A, Azuma H, Hayashi N, et al Efficacy and safety of dapagliflozin as a monotherapy for type 2 diabetes mellitus in Japanese patients with inadequate glycaemic control: a phase II multicentre, randomized, double-blind, placebo-controlled trial. Diabetes Obes Metab. 2013;15(5):432–40. 10.1111/dom.12047 23194084

[69] Kaku K, Kiyosue A, Inoue S, Ueda N, Tokudome T, Yang J, et al Efficacy and safety of dapagliflozin monotherapy in Japanese patients with type 2 diabetes inadequately controlled by diet and exercise. Diabetes Obes Metab. 2014;16(11):1102–10. 10.1111/dom.12325 24909293

[70] Lambers Heerspink HJ, de Zeeuw D, Wie L, Leslie B, List J. Dapagliflozin a glucose-regulating drug with diuretic properties in subjects with type 2 diabetes. Diabetes Obes Metab. 2013;15(9):853–62. 10.1111/dom.12127 23668478 PMC3906841

[71] Leiter LA, Cefalu WT, de Bruin TW, Gause-Nilsson I, Sugg J, Parikh SJ. Dapagliflozin added to usual care in individuals with type 2 diabetes mellitus with preexisting cardiovascular disease: a 24-week, multicenter, randomized, double-blind, placebo-controlled study with a 28-week extension. J Am Geriatr Soc. 2014;62(7):1252–62. 10.1111/jgs.12881 24890683

[72] Ljunggren O, Bolinder J, Johansson L, Wilding J, Langkilde AM, Sjostrom CD, et al Dapagliflozin has no effect on markers of bone formation and resorption or bone mineral density in patients with inadequately controlled type 2 diabetes mellitus on metformin. Diabetes Obes Metab. 2012;14(11):990–9. 10.1111/j.1463-1326.2012.01630.x 22651373

[73] Mathieu C, Ranetti AE, Li D, Ekholm E, Cook W, Hirshberg B, et al Randomized, double-blind, phase 3 trial of triple therapy with dapagliflozin add-on to saxagliptin plus metformin in type 2 diabetes. Diabetes Care. 2015;38(11):2009–17. 10.2337/dc15-0779 26246458

[74] Matthaei S, Bowering K, Rohwedder K, Grohl A, Parikh S. Dapagliflozin improves glycemic control and reduces body weight as add-on therapy to metformin plus sulfonylurea: a 24-week randomized, double-blind clinical trial. Diabetes Care. 2015;38(3):365–72. 10.2337/dc14-0666 25592197

[75] Matthaei S, Bowering K, Rohwedder K, Sugg J, Parikh S, Johnsson E, et al Durability and tolerability of dapagliflozin over 52 weeks as add-on to metformin and sulphonylurea in type 2 diabetes. Diabetes Obes Metab. 2015;17(11):1075–84. 10.1111/dom.12543 26212528

[76] Rosenstock J, Vico M, Wei L, Salsali A, List JF. Effects of dapagliflozin, an SGLT2 inhibitor, on HbA(1c), body weight, and hypoglycemia risk in patients with type 2 diabetes inadequately controlled on pioglitazone monotherapy. Diabetes Care. 2012;35(7):1473–8. 10.2337/dc11-1693 22446170 PMC3379599

[77] Strojek K, Yoon KH, Hruba V, Elze M, Langkilde AM, Parikh S. Effect of dapagliflozin in patients with type 2 diabetes who have inadequate glycaemic control with glimepiride: a randomized, 24-week, double-blind, placebo-controlled trial. Diabetes Obes Metab. 2011;13(10):928–38. 10.1111/j.1463-1326.2011.01434.x 21672123

[78] Strojek K, Yoon KH, Hruba V, Sugg J, Langkilde AM, Parikh S. Dapagliflozin added to glimepiride in patients with type 2 diabetes mellitus sustains glycemic control and weight loss over 48 weeks: a randomized, double-blind, parallel-group, placebo-controlled trial. Diabetes Ther. 2014;5(1):267–83. 10.1007/s13300-014-0072-0 24920277 PMC4065289

[79] Wilding JP, Norwood P, T'Joen C, Bastien A, List JF, Fiedorek FT. A study of dapagliflozin in patients with type 2 diabetes receiving high doses of insulin plus insulin sensitizers: applicability of a novel insulin-independent treatment. Diabetes Care. 2009;32(9):1656–62. 10.2337/dc09-0517 19528367 PMC2732143

[80] Ferrannini E, Berk A, Hantel S, Pinnetti S, Hach T, Woerle HJ, et al Long-term safety and efficacy of empagliflozin, sitagliptin, and metformin: an active-controlled, parallel-group, randomized, 78-week open-label extension study in patients with type 2 diabetes. Diabetes Care. 2013;36(12):4015–21. 10.2337/dc13-0663 24186878 PMC3836134

[81] Ferrannini E, Seman L, Seewaldt-Becker E, Hantel S, Pinnetti S, Woerle HJ. A Phase IIb, randomized, placebo-controlled study of the SGLT2 inhibitor empagliflozin in patients with type 2 diabetes. Diabetes Obes Metab. 2013;15(8):721–8. 10.1111/dom.12081 23398530

[82] Haring HU, Merker L, Seewaldt-Becker E, Weimer M, Meinicke T, Broedl UC, et al Empagliflozin as add-on to metformin in patients with type 2 diabetes: a 24-week, randomized, double-blind, placebo-controlled trial. Diabetes Care. 2014;37(6):1650–9. 10.2337/dc13-2105 24722494

[83] Kadowaki T, Haneda M, Inagaki N, Terauchi Y, Taniguchi A, Koiwai K, et al Empagliflozin monotherapy in Japanese patients with type 2 diabetes mellitus: a randomized, 12-week, double-blind, placebo-controlled, phase II trial. Adv Ther. 2014;31(6):621–38. 10.1007/s12325-014-0126-8 24958326

[84] Merker L, Haring HU, Christiansen AV, Roux F, Salsali A, Kim G, et al Empagliflozin as add-on to metformin in people with Type 2 diabetes. Diabet Med. 2015;32(12):1555–67. 10.1111/dme.12814 26031566

[85] Rosenstock J, Seman LJ, Jelaska A, Hantel S, Pinnetti S, Hach T, et al Efficacy and safety of empagliflozin, a sodium glucose cotransporter 2 (SGLT2) inhibitor, as add-on to metformin in type 2 diabetes with mild hyperglycaemia. Diabetes Obes Metab. 2013;15(12):1154–60. 10.1111/dom.12185 23906374

[86] Rosenstock J, Jelaska A, Frappin G, Salsali A, Kim G, Woerle HJ, et al Improved glucose control with weight loss, lower insulin doses, and no increased hypoglycemia with empagliflozin added to titrated multiple daily injections of insulin in obese inadequately controlled type 2 diabetes. Diabetes Care. 2014;37(7):1815–23. 10.2337/dc13-3055 24929430

[87] Rosenstock J, Jelaska A, Zeller C, Kim G, Broedl UC, Woerle HJ, et al Impact of empagliflozin added on to basal insulin in type 2 diabetes inadequately controlled on basal insulin: a 78-week randomized, double-blind, placebo-controlled trial. Diabetes Obes Metab. 2015;17(10):936–48. 10.1111/dom.12503 26040302 PMC5034797

[88] Ross S, Thamer C, Cescutti J, Meinicke T, Woerle HJ, Broedl UC. Efficacy and safety of empagliflozin twice daily versus once daily in patients with type 2 diabetes inadequately controlled on metformin: a 16-week, randomized, placebo-controlled trial. Diabetes Obes Metab. 2015;17(7):699–702. 10.1111/dom.12469 25827441

[89] Cefalu WT, Leiter LA, Yoon KH, Arias P, Niskanen L, Xie J, et al Efficacy and safety of canagliflozin versus glimepiride in patients with type 2 diabetes inadequately controlled with metformin (CANTATA-SU): 52 week results from a randomised, double-blind, phase 3 non-inferiority trial. Lancet. 2013;382(9896):941–50. 10.1016/S0140-6736(13)60683-2 23850055

[90] Leiter LA, Yoon KH, Arias P, Langslet G, Xie J, Balis DA, et al Canagliflozin provides durable glycemic improvements and body weight reduction over 104 weeks versus glimepiride in patients with type 2 diabetes on metformin: a randomized, double-blind, phase 3 study. Diabetes Care. 2015;38(3):355–64. 10.2337/dc13-2762 25205142

[91] Schernthaner G, Gross JL, Rosenstock J, Guarisco M, Fu M, Yee J, et al Canagliflozin compared with sitagliptin for patients with type 2 diabetes who do not have adequate glycemic control with metformin plus sulfonylurea: a 52-week randomized trial. Diabetes Care. 2013;36(9):2508–15. 10.2337/dc12-2491 23564919 PMC3747923

[92] Henry RR, Murray AV, Marmolejo MH, Hennicken D, Ptaszynska A, List JF. Dapagliflozin, metformin XR, or both: initial pharmacotherapy for type 2 diabetes, a randomised controlled trial. Int J Clin Pract. 2012;66(5):446–56. 10.1111/j.1742-1241.2012.02911.x 22413962

[93] Del Prato S, Nauck M, Duran-Garcia S, Maffei L, Rohwedder K, Theuerkauf A et al Long-term glycaemic response and tolerability of dapagliflozin verus a sulphonylurea as add-on therapy to metformin in patients with type 2 diabetes: 4-year data. Diabetes Obes Metab. 2015;17(6):581–90. 10.1111/dom.12459 25735400

[94] Nauck MA, Del Prato S, Meier JJ, Duran-Garcia S, Rohwedder K, Elze M, et al Dapagliflozin versus glipizide as add-on therapy in patients with type 2 diabetes who have inadequate glycemic control with metformin: a randomized, 52-week, double-blind, active-controlled noninferiority trial. Diabetes Care. 2011;34(9):2015–22. 10.2337/dc11-0606 21816980 PMC3161265

[95] Nauck MA, Del Prato S, Duran-Garcia S, Rohwedder K, Langkilde AM, Sugg J, et al Durability of glycaemic efficacy over 2 years with dapagliflozin versus glipizide as add-on therapies in patients whose type 2 diabetes mellitus is inadequately controlled with metformin. Diabetes Obes Metab. 2014;16(11):1111–20. 10.1111/dom.12327 24919526

[96] Rosenstock J, Hansen L, Zee P, Li Y, Cook W, Hirshberg B, et al Dual add-on therapy in type 2 diabetes poorly controlled with metformin monotherapy: a randomized double-blind trial of saxagliptin plus dapagliflozin addition versus single addition of saxagliptin or dapagliflozin to metformin. Diabetes Care. 2015;38(3):376–83. 10.2337/dc14-1142 25352655

[97] DeFronzo RA, Lewin A, Patel S, Liu D, Kaste R, Woerle HJ, et al Combination of empagliflozin and linagliptin as second-line therapy in subjects with type 2 diabetes inadequately controlled on metformin. Diabetes Care. 2015;38(3):384–93. 10.2337/dc14-2364 25583754

[98] Lewin A, DeFronzo RA, Patel S, Liu D, Kaste R, Woerle HJ, et al Initial combination of empagliflozin and linagliptin in subjects with type 2 diabetes. Diabetes Care. 2015;38(3):394–402. 10.2337/dc14-2365 25633662

[99] Ridderstrale M, Svaerd R, Zeller C, Kim G, Woerle HJ, Broedl UC. Rationale, design and baseline characteristics of a 4-year (208-week) phase III trial of empagliflozin, an SGLT2 inhibitor, versus glimepiride as add-on to metformin in patients with type 2 diabetes mellitus with insufficient glycemic control. Cardiovasc Diabetol. 2013;12:129-. 10.1186/1475-2840-12-129 24007456 PMC3844307

[100] Ridderstråle M, Andersen KR, Zeller C, Kim G, Woerle HJ, Broedl UC. Comparison of empagliflozin and glimepiride as add-on to metformin in patients with type 2 diabetes: a 104-week randomised, active-controlled, double-blind, phase 3 trial. Lancet Diabetes Endocrinol. 2014;2:691–700. 10.1016/S2213-8587(14)70120-2 24948511

[101] Laakso M. Cardiovascular disease in type 2 diabetes from population to man to mechanisms: the Kelly West Award Lecture 2008. Diabetes Care. 2010;33(2):442–9. 10.2337/dc09-0749 20103560 PMC2809299

[102] Ahmed MH, Husain NE, Almobarak AO. Nonalcoholic Fatty liver disease and risk of diabetes and cardiovascular disease: what is important for primary care physicians? J Family Med Prim Care. 2015;4(1):45–52. 10.4103/2249-4863.152252 25810989 PMC4367006

[103] Liu H, Lu HY. Nonalcoholic fatty liver disease and cardiovascular disease. World J Gastroenterol. 2014;20(26):8407–15. 10.3748/wjg.v20.i26.8407 25024598 PMC4093693

[104] Yilmaz Y. Liver function tests: Association with cardiovascular outcomes. World J Hepatol. 2010;2(4):143–5. 10.4254/wjh.v2.i4.143 21160986 PMC2999279

[105] Leiter LA, Forst T, Polidori D, Balis DA, Xie J, Sha S. Effect of canagliflozin on liver function tests in patients with type 2 diabetes. Diabetes Metab. 2016;42(1):25–32. 10.1016/j.diabet.2015.10.003 26575250

[106] Kalra S. Sodium glucose co-transporter-2 (SGLT2) inhibitors: A review of their basic and clinical pharmacology. Diabetes Ther. 2014;5(2):355–66. 10.1007/s13300-014-0089-4 25424969 PMC4269649

[107] Jose P, Skali H, Anavekar N, Tomson C, Krumholz HM, Rouleau JL, et al Increase in creatinine and cardiovascular risk in patients with systolic dysfunction after myocardial infarction. J Am Soc Nephrol. 2006;17(10):2886–91. 10.1681/ASN.2006010063 16928807

[108] Damman K, Navis G, Voors AA, Asselbergs FW, Smilde TD, Cleland JG, et al Worsening renal function and prognosis in heart failure: systematic review and meta-analysis. J Card Fail. 2007;13(8):599–608. 10.1016/j.cardfail.2007.04.008 17923350

[109] Sarnak M, Levey A, Schoolwerth A, Coresh J, Culleton B, Hamm L, et al Kidney disease as a risk factor for development of cardiovascular disease a statement from the American Heart Association Councils on kidney in cardiovascular disease, high blood pressure research, clinical cardiology, and epidemiology and prevention. Circulation. 2003;108(17):2154–69. 14581387 10.1161/01.CIR.0000095676.90936.80

[110] Storgaard H, Bagger JI, Knop FK, Vilsboll T, Rungby J. Diabetic Ketoacidosis in a Patient with Type 2 Diabetes After Initiation of Sodium-Glucose Cotransporter 2 Inhibitor Treatment. Basic Clin Pharmacol Toxicol. 2016;118(2):168–70. 10.1111/bcpt.12457 26291182

[111] Ferrannini E, Muscelli E, Frascerra S, Baldi S, Mari A, Heise T, et al Metabolic response to sodium-glucose cotransporter 2 inhibition in type 2 diabetic patients. J Clin Invest. 2014;124(2):499–508. 10.1172/jci72227 24463454 PMC3904627

