# Supplementary figures and images for: Benefits and Harms of Sodium-Glucose Co-Transporter 2 Inhibitors in Patients with Type 2 Diabetes: A Systematic Review and Meta-Analysis
Source: PLoS One. 2016 Nov 11;11(11):e0166125. doi: 10.1371/journal.pone.0166125 (PMC5106000; doi:10.1371/journal.pone.0166125)

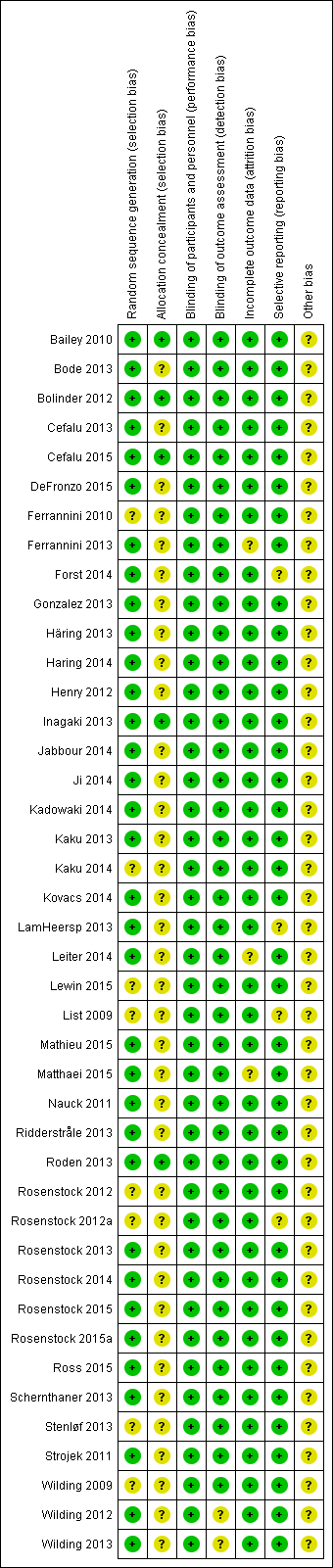

Supplement: S1 Fig — Low risk of bias: ‘+’ in green circle; unclear risk of bias ‘?’ in yellow circle; no studies were at high risk of bias in any domain. (TIF) [file pone.0166125.s001.tif]

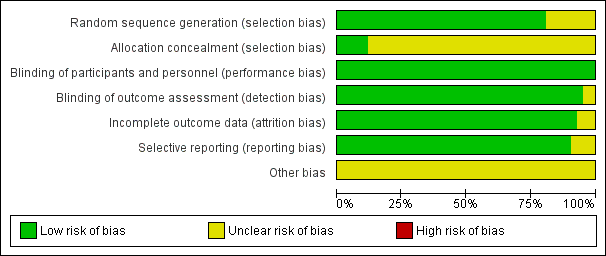

Supplement: S2 Fig — (TIF) [file pone.0166125.s002.tif]
