# Supplementary material for: Benefits and Harms of Sodium-Glucose Co-Transporter 2 Inhibitors in Patients with Type 2 Diabetes: A Systematic Review and Meta-Analysis
Source: PLoS One. 2016 Nov 11;11(11):e0166125. doi: 10.1371/journal.pone.0166125 (PMC5106000; doi:10.1371/journal.pone.0166125)
Supplement: S1 File — Multiple publications which reported the same RCT were grouped into ‘studies’. (PDF) [file pone.0166125.s003.pdf]

S1 Appendix. Data sources: multiple publications which reported the same RCT study were grouped into 'studies'

*Indicates primary study

Included studies

Bailey 2010

Bailey CJ, Gross JL, Hennicken D, Iqbal N, Mansfield TA, List JF. Correction: Dapagliflozin add-on to metformin in type 2 diabetes inadequately controlled with metformin: a randomized, double-blind, placebo-controlled 102-week trial. BMC medicine 2013;11:193.

Bailey CJ, Gross JL, Hennicken D, Iqbal N, Mansfield TA, List JF. Dapagliflozin add-on to metformin in type 2 diabetes inadequately controlled with metformin: a randomized, double-blind, placebo-controlled 102-week trial. BMC medicine 2013;11:741-7015. [Other: 1]

* Bailey CJ, Gross JL, Pieters A, Bastien A, List JF. Effect of dapagliflozin in patients with type 2 diabetes who have inadequate glycaemic control with metformin: a randomised, double-blind, placebo-controlled trial. Lancet 2010;375(9733):2223-33. [Other: 1474-547X]

Kyle Nahrebne on behalf of AstraZeneca Pharmaceuticals. Additional data CSRs: LDL given in percentage change from baseline, ALAT, serum creatinine, heart rate, systolic and diastolic blood pressure. Overall adverse events (all types) tables by System Organ Class (SOC), other adverse events including fractures. Email communication on July 7 2014.

Bode 2013

Bode B, Stenlöf K, Sullivan D, Fung A, Usiskin K. Efficacy and safety of canagliflozin treatment in older subjects with type 2 diabetes mellitus: a randomized trial. Hospital practice 2013;41(2):72-84. [DOI: 10.3810/hp.2013.04.1020]

* Bode B, Stenlof K, Harris S, Sullivan D, Fung A, Usiskin K, et al. Long-term efficacy and safety of canagliflozin over 104 weeks in patients aged 55-80 years with type 2 diabetes. Diabetes, obesity & metabolism 2015;17(3):294-303. [PubMed: 25495720]

Karla Childers on behalf of Janssen Pharmaceuticals, Inc, a pharmaceutical company of Johnson & Johnson. Additional data: HDL and triglyceride absolute changes of ALAT, LDL, and serum creatinine. Overall adverse events (all types) tables by System Organ Class (SOC), other adverse events including fractures. The YODA-project Email communication on December 12 2014.

Bolinder 2012

* Bolinder J, Ljunggren Ö, Kullberg J, Johansson L, Wilding J, Langkilde AM, et al. Effects of dapagliflozin on body weight, total fat mass, and regional adipose tissue distribution in

patients with type 2 diabetes mellitus with inadequate glycemic control on metformin. The Journal of clinical endocrinology and metabolism 2012;97(3):1020-31. [Other: 1945-7197]

Bolinder J, Ljunggren O, Johansson L, Wilding J, Langkilde AM, Sjöström CD, et al. Dapagliflozin maintains glycaemic control while reducing weight and body fat mass over 2 years in patients with type 2 diabetes mellitus inadequately controlled on metformin. Diabetes, obesity & metabolism 2013. [Other: 1463-1326]

Ljunggren O, Bolinder J, Johansson L, Wilding J, Langkilde AM, Sjostrom CD, et al. Dapagliflozin has no effect on markers of bone formation and resorption or bone mineral density in patients with inadequately controlled type 2 diabetes mellitus on metformin. Diabetes, obesity & metabolism 2012;14(11):990-9. [PubMed: 22651373]

Kyle Nahrebne on behalf of AstraZeneca Pharmaceuticals. Additional data CSRs: LDL given in percentage change from baseline, ALAT, serum creatinine, heart rate, systolic and diastolic blood pressure. Overall adverse events (all types) tables by System Organ Class (SOC), other adverse events including fractures. Email communication on July 7 2014.

Cefalu 2013

* Cefalu WT, Leiter LA, Yoon K-H, Arias P, Niskanen L, Xie J, et al. Efficacy and safety of canagliflozin versus glimepiride in patients with type 2 diabetes inadequately controlled with metformin (CANTATA-SU): 52 week results from a randomised, double-blind, phase 3 non-inferiority trial. Lancet 2013;382(9896):941-50. [Other: 1474-547X]

Leiter LA, Yoon KH, Arias P, Langslet G, Xie J, Balis DA, et al. Canagliflozin provides durable glycemic improvements and body weight reduction over 104 weeks versus glimepiride in patients with type 2 diabetes on metformin: a randomized, double-blind, phase 3 study. Diabetes care 2015;38(3):355-64. [PubMed: 25205142]

Karla Childers on behalf of Janssen Pharmaceuticals, Inc, a pharmaceutical company of Johnson & Johnson. Additional data: HDL and triglyceride absolute changes of ALAT, LDL, and serum creatinine. Overall adverse events (all types) tables by System Organ Class (SOC), other adverse events including fractures. The YODA-project Email communication on December 12 2014.

Cefalu 2015

* Cefalu W T, Leiter L A, de Bruin T W, Gause-Nilsson I, Sugg J, Parikh S J. Dapagliflozin's effects on glycemia and cardiovascular risk factors in high-risk patients with type 2 diabetes: a 24-week, multicenter, randomized, double-blind, placebo-controlled study with a 28-week extension. Diabetes Care 2015;38:1218-27. [DOI: 10.2337/dc14-0315]

DeFronzo 2015

DeFronzo RA, Lewin A, Patel S, Liu D, Kaste R, Woerle HJ, et al. Combination of empagliflozin and linagliptin as second-line therapy in subjects with type 2 diabetes inadequately controlled on metformin. Diabetes care 2015;38(3):384-93. [PubMed: 25583754]

Ferrannini 2010

* Ferrannini E, Ramos SJ, Salsali A, Tang W, List JF. Dapagliflozin monotherapy in type 2 diabetic patients with inadequate glycemic control by diet and exercise: a randomized, double-blind, placebo-controlled, phase 3 trial. Diabetes care 2010;33(10):2217-24. [Other: 1935-5548]

Kyle Nahrebne on behalf of AstraZeneca Pharmaceuticals. Additional data CSRs: LDL given in percentage change from baseline, ALAT, serum creatinine, heart rate, systolic and diastolic blood pressure. Overall adverse events (all types) tables by System Organ Class (SOC), other adverse events including fractures. Email communication on July 7 2014.

Ferrannini 2013

Ferrannini E, Berk A, Hantel S, Pinnetti S, Hach T, Woerle HJ, et al. Long-term safety and efficacy of empagliflozin, sitagliptin, and metformin: an active-controlled, parallel-group, randomized, 78-week open-label extension study in patients with type 2 diabetes. Diabetes care 2013;36(12):4015-21. [PubMed: 24186878]

* Ferrannini E, Seman L, Seewaldt-Becker E, Hantel S, Pinnetti S, Woerle HJ. A Phase IIb, randomized, placebo-controlled study of the SGLT2 inhibitor empagliflozin in patients with type 2 diabetes. Diabetes, obesity & metabolism 2013;15(8):721-8. [PubMed: 23398530]

Søren Lund on behalf of Boehringer Ingelheim Pharma GmbH & Co. Additional data: LDL, ALAT, serum creatinine, heart rate, systolic and diastolic blood pressure. Adverse events fractures, all cancers, bladder and breast cancer. Email communication on December 5 2014.

Forst 2014

* Forst T, Guthrie R, Goldenberg R, Yee J, Vijapurkar U, Meininger G, et al. Efficacy and safety of canagliflozin over 52 weeks in patients with type 2 diabetes on background metformin and pioglitazone. Diabetes, obesity & metabolism 2014;16(5):467-77. [Other: 1463-1326]

Karla Childers on behalf of Janssen Pharmaceuticals, Inc, a pharmaceutical company of Johnson & Johnson. Additional data: HDL and triglyceride absolute changes of ALAT, LDL, and serum creatinine. Overall adverse events (all types) tables by System Organ Class (SOC), other adverse events including fractures. The YODA-project Email communication on December 12 2014.

Gonzalez 2013

* Lavalle-González FJ, Januszewicz A, Davidson J, Tong C, Qiu R, Canovatchel W, et al. Efficacy and safety of canagliflozin compared with placebo and sitagliptin in patients with type 2 diabetes on background metformin monotherapy: a randomised trial. Diabetologia 2013. [Other: 1432-0428]

Karla Childers on behalf of Janssen Pharmaceuticals, Inc, a pharmaceutical company of Johnson & Johnson. Additional data: HDL and triglyceride absolute changes of ALAT, LDL, and serum creatinine. Overall adverse events (all types) tables by System Organ Class (SOC), other adverse events including fractures. The YODA-project Email communication on December 12 2014.

Häring 2013

* Häring HU, Merker L, Seewaldt-Becker E, Weimer M, Meinicke T, Woerle HJ, et al. Empagliflozin as add-on to metformin plus sulfonylurea in patients with type 2 diabetes: a 24-week, randomized, double-blind, placebo-controlled trial. Diabetes care 2013;36(11):3396-404. [Other: 1935-5548]

Haering HU, Merker L, Christiansen AV, Roux F, Salsali A, Kim G, et al. Empagliflozin as add-on to metformin plus sulphonylurea in patients with type 2 diabetes. Diabetes Research and Clinical Practice 2015;110(1):82-90. [DOI: 10.1016/j.diabres.2015.05.044]

Søren Lund on behalf of Boehringer Ingelheim Pharma GmbH & Co. Additional data: LDL, ALAT, serum creatinine, heart rate, systolic and diastolic blood pressure. Adverse events fractures, all cancers, bladder and breast cancer. Email communication on December 5 2014.

Haring 2014

* Haring HU, Merker L, Seewaldt-Becker E, Weimer M, Meinicke T, Broedl U C, et al. Empagliflozin as add-on to metformin in patients with type 2 diabetes: a 24-week, randomized, double-blind, placebo-controlled trial. Diabetes Care 2014;37:1650-9.

Merker L, Haring H U, Christiansen A V, Roux F, Salsali A, Kim G, et al. Empagliflozin as add-on to metformin in people with Type 2 diabetes. Diabetic Medicine 2015;12:1555-1567.

Henry 2012

* Henry RR, Murray AV, Marmolejo MH, Hennicken D, Ptaszynska A, List JF. Dapagliflozin, metformin XR, or both: initial pharmacotherapy for type 2 diabetes, a randomised controlled trial. International journal of clinical practice 2012;66(5):446-56. [Other: 1742-1241]

Kyle Nahrebne on behalf of AstraZeneca Pharmaceuticals. Additional data CSRs: LDL given in percentage change from baseline, ALAT, serum creatinine, heart rate, systolic and diastolic blood pressure. Overall adverse events (all types) tables by System Organ Class (SOC), other adverse events including fractures. Email communication on July 7 2014.

Inagaki 2013

Inagaki N, Kondo K, Yoshinari T, Maruyama N, Susuta Y, Kuki H. Efficacy and safety of canagliflozin in Japanese patients with type 2 diabetes: a randomized, double-blind,

placebo-controlled, 12-week study. Diabetes, obesity & metabolism 2013;15(11):1501-15. [Other: 1463-1326]

Jabbour 2014

* Jabbour SA, Hardy E, Sugg J, Parikh S, for the Study G. Dapagliflozin is effective as add-on therapy to sitagliptin with or without metformin: a 24-week, multicenter, randomized, double-blind, placebo-controlled study. Diabetes care 2014. [Other: 1935-5548]

Kyle Nahrebne on behalf of AstraZeneca Pharmaceuticals. Additional data CSRs: LDL given in percentage change from baseline, ALAT, serum creatinine, heart rate, systolic and diastolic blood pressure. Overall adverse events (all types) tables by System Organ Class (SOC), other adverse events including fractures. Email communication on July 7 2014.

Ji 2014

Ji L, Ma J, Li H, Mansfield TA, T'Joen CL, Iqbal N, et al. Dapagliflozin as monotherapy in drug-naive Asian patients with type 2 diabetes mellitus: a randomized, blinded, prospective phase III study. Clinical therapeutics 2014;36(1):84-100.e9. [Other: 1879-114X]

Kyle Nahrebne on behalf of AstraZeneca Pharmaceuticals. Additional data CSRs: LDL given in percentage change from baseline, ALAT, serum creatinine, heart rate, systolic and diastolic blood pressure. Overall adverse events (all types) tables by System Organ Class (SOC), other adverse events including fractures. Email communication on July 7 2014.

Kadowaki 2014

Kadowaki T, Haneda M, Inagaki N, Terauchi Y, Taniguchi A, Koiwai K, et al. Empagliflozin monotherapy in Japanese patients with type 2 diabetes mellitus: a randomized, 12-week, double-blind, placebo-controlled, phase II trial. Advances in therapy 2014;31(6):621-38. [PubMed: 24958326]

Kaku 2013

* Kaku K, Inoue S, Matsuoka O, Kiyosue A, Azuma H, Hayashi N, et al. Efficacy and safety of dapagliflozin as a monotherapy for type 2 diabetes mellitus in Japanese patients with inadequate glycaemic control: a phase II multicentre, randomized, double-blind, placebo-controlled trial. Diabetes, obesity & metabolism 2013;15(5):432-40. [Other: 1463-1326]

Kyle Nahrebne on behalf of AstraZeneca Pharmaceuticals. Additional data CSRs: LDL given in percentage change from baseline, ALAT, serum creatinine, heart rate, systolic and diastolic blood pressure. Overall adverse events (all types) tables by System Organ Class (SOC), other adverse events including fractures. Email communication on July 7 2014.

Kaku 2014

* Kaku K, Kiyosue A, Inoue S, Ueda N, Tokudome T, Yang J, et al. Efficacy and safety of dapagliflozin monotherapy in Japanese patients with type 2 diabetes inadequately

controlled by diet and exercise. Diabetes, obesity & metabolism 2014;16(11):1102-10. [PubMed: 24909293]

Kovacs 2014

Kovacs C S, Seshiah V, Merker L, Christiansen A V, Roux F, Salsali A, et al. Empagliflozin as add-on therapy to pioglitazone with or without metformin in patients with type 2 diabetes mellitus. Clinical Therapeutics 2015;37:1773-1788 e1. [DOI: 10.1016/j.clinthera.2015.05.511]

* Kovacs CS, Seshiah V, Swallow R, Jones R, Rattunde H, Woerle HJ, et al. Empagliflozin improves glycaemic and weight control as add-on therapy to pioglitazone or pioglitazone plus metformin in patients with type 2 diabetes: a 24-week, randomized, placebo-controlled trial. Diabetes, obesity & metabolism 2014;16(2):147-58. [Other: 1463-1326]

Lambers Heerspink 2013

* Lambers Heerspink HJ, de Zeeuw D, Wie L, Leslie B, List J. Dapagliflozin a glucose-regulating drug with diuretic properties in subjects with type 2 diabetes. Diabetes, obesity & metabolism 2013;15(9):853-62. [PubMed: 23668478]

Kyle Nahrebne on behalf of AstraZeneca Pharmaceuticals. Additional data CSRs: LDL given in percentage change from baseline, ALAT, serum creatinine, heart rate, systolic and diastolic blood pressure. Overall adverse events (all types) tables by System Organ Class (SOC), other adverse events including fractures. Email communication on July 7 2014.

Leiter 2014

Leiter LA, Cefalu WT, de Bruin TW, Gause-Nilsson I, Sugg J, Parikh SJ. Dapagliflozin added to usual care in individuals with type 2 diabetes mellitus with preexisting cardiovascular disease: a 24-week, multicenter, randomized, double-blind, placebo-controlled study with a 28-week extension. Journal of the American Geriatrics Society 2014;62(7):1252-62. [PubMed: 24890683]

Lewin 2015

Lewin A, DeFronzo RA, Patel S, Liu D, Kaste R, Woerle HJ, et al. Initial combination of empagliflozin and linagliptin in subjects with type 2 diabetes. Diabetes care 2015;38(3):394-402. [PubMed: 25633662]

List 2009

* List JF, Woo V, Morales E, Tang W, Fiedorek FT. Sodium-glucose cotransport inhibition with dapagliflozin in type 2 diabetes. Diabetes care 2009;32(4):650-7. [DOI: 10.2337/dc08-1863]

Kyle Nahrebne on behalf of AstraZeneca Pharmaceuticals. Additional data CSRs: LDL given in percentage change from baseline, ALAT, serum creatinine, heart rate, systolic and diastolic

blood pressure. Overall adverse events (all types) tables by System Organ Class (SOC), other adverse events including fractures. Email communication on July 7 2014.

Mathieu 2015

Mathieu C, Ranetti A E, Li D, Ekholm E, Cook W, Hirshberg B, et al. A randomized, double-blind, phase 3 trial of triple therapy with dapagliflozin add-on to saxagliptin plus metformin in type 2 diabetes. Diabetes Care 2015;38(11):2009-17. [DOI: 10.2337/dc15-0779]

Matthaei 2015

* Matthaei S, Bowering K, Rohwedder K, Grohl A, Parikh S. Dapagliflozin improves glycemic control and reduces body weight as add-on therapy to metformin plus sulfonylurea: a 24-week randomized, double-blind clinical trial. Diabetes care 2015;38(3):365-72. [PubMed: 25592197]

Matthaei S, Bowering K, Rohwedder K, Sugg J, Parikh S, Johnsson E, et al. Durability and tolerability of dapagliflozin over 52 weeks as add-on to metformin and sulphonylurea in type 2 diabetes. Diabetes, obesity and metabolism 2015;17:1075-84. [DOI: 10.1111/dom.12543]

Nauck 2011

Del Prato S, Nauck M, Duran-Garcia S, Maffei L, Rohwedder K, Theuerkauf A et al. Long-term glycaemic response and tolerability of dapagliflozin verus a sulphonylurea as add-on therapy to metformin in patients with type 2 diabetes: 4-year data. Diabetes obesity and metabolism 2015;17(6):581-90. [DOI: doi: 10.1111/dom.12459]

Nauck MA, Del Prato S, Duran-Garcia S, Rohwedder K, Langkilde AM, Sugg J, et al. Durability of glycaemic efficacy over 2 years with dapagliflozin versus glipizide as add-on therapies in patients whose type 2 diabetes mellitus is inadequately controlled with metformin. Diabetes, obesity & metabolism 2014;16(11):1111-20. [PubMed: 24919526]

* Nauck MA, Del Prato S, Meier JJ, Durán-García S, Rohwedder K, Elze M, et al. Dapagliflozin versus glipizide as add-on therapy in patients with type 2 diabetes who have inadequate glycemic control with metformin: a randomized, 52-week, double-blind, active-controlled noninferiority trial. Diabetes care 2011;34(9):2015-22. [Other: 1935-5548]

Kyle Nahrebne on behalf of AstraZeneca Pharmaceuticals. Additional data CSRs: LDL given in percentage change from baseline, ALAT, serum creatinine, heart rate, systolic and diastolic blood pressure. Overall adverse events (all types) tables by System Organ Class (SOC), other adverse events including fractures. Email communication on July 7 2014.

Ridderstråle 2013

Ridderstråle M, Andersen KR, Zeller C, Kim G, Woerle HJ, Broedl UC. Comparison of empagliflozin and glimepiride as add-on to metformin in patients with type 2 diabetes: a 104-week randomised, active-controlled, double-blind, phase 3 trial. The Lancet Diabetes & Endocrinology 2014;2(9):691-700.

* Ridderstrale M, Svaerd R, Zeller C, Kim G, Woerle HJ, Broedl UC. Rationale, design and baseline characteristics of a 4-year (208-week) phase III trial of empagliflozin, an SGLT2 inhibitor, versus glimepiride as add-on to metformin in patients with type 2 diabetes mellitus with insufficient glycemic control. Cardiovascular diabetology 2013;12:129. [PubMed: 24007456]

Roden 2013

* Roden M, Weng J, Eilbracht J, Delafont B, Kim G, Woerle HJ, et al. Empagliflozin monotherapy with sitagliptin as an active comparator in patients with type 2 diabetes: a randomised, double-blind, placebo-controlled, phase 3 trial. The lancet. Diabetes & endocrinology 2013;1(3):208-19. [Other: 2213-8595]

Søren Lund on behalf of Boehringer Ingelheim Pharma GmbH & Co. Additional data: LDL, ALAT, serum creatinine, heart rate, systolic and diastolic blood pressure. Adverse events fractures, all cancers, bladder and breast cancer. Email communication on December 5 2014.

Rosenstock 2012

* Rosenstock J, Aggarwal N, Polidori D, Zhao Y, Arbit D, Usiskin K, et al. Dose-ranging effects of canagliflozin, a sodium-glucose cotransporter 2 inhibitor, as add-on to metformin in subjects with type 2 diabetes. Diabetes care 2012;35(6):1232-8. [Other: 1935-5548]

Karla Childers on behalf of Janssen Pharmaceuticals, Inc, a pharmaceutical company of Johnson & Johnson. Additional data: HDL and triglyceride absolute changes of ALAT, LDL, and serum creatinine. Overall adverse events (all types) tables by System Organ Class (SOC), other adverse events including fractures. The YODA-project Email communication on December 12 2014.

Rosenstock 2012a

Rosenstock J, Vico M, Wei L, Salsali A, List JF. Effects of dapagliflozin, an SGLT2 inhibitor, on HbA(1c), body weight, and hypoglycemia risk in patients with type 2 diabetes inadequately controlled on pioglitazone monotherapy. Diabetes care 2012;35(7):1473-8. [Other: 1935-5548]

Kyle Nahrebne on behalf of AstraZeneca Pharmaceuticals. Additional data CSRs: LDL given in percentage change from baseline, ALAT, serum creatinine, heart rate, systolic and diastolic blood pressure. Overall adverse events (all types) tables by System Organ Class (SOC), other adverse events including fractures. Email communication on July 7 2014.

Rosenstock 2013

* Rosenstock J, Seman LJ, Jelaska A, Hantel S, Pinnetti S, Hach T, et al. Efficacy and safety of empagliflozin, a sodium glucose cotransporter 2 (SGLT2) inhibitor, as add-on to metformin in type 2 diabetes with mild hyperglycaemia. Diabetes, obesity & metabolism 2013. [Other: 1463-1326]

Søren Lund on behalf of Boehringer Ingelheim Pharma GmbH & Co. Additional data: LDL, ALAT, serum creatinine, heart rate, systolic and diastolic blood pressure. Adverse events fractures, all cancers, bladder and breast cancer. Email communication on December 5 2014.

Rosenstock 2014

Rosenstock J, Jelaska A, Frappin G, Salsali A, Kim G, Woerle HJ, et al. Improved glucose control with weight loss, lower insulin doses, and no increased hypoglycemia with empagliflozin added to titrated multiple daily injections of insulin in obese inadequately controlled type 2 diabetes. Diabetes care 2014;37(7):1815-23. [PubMed: 24929430]

Rosenstock 2015

Rosenstock J, Jelaska A, Zeller C, Kim G, Broedl U C, Woerle H J, et al. Impact of empagliflozin added on to basal insulin in type 2 diabetes inadequately controlled on basal insulin: a 78-week randomized, double-blind, placebo-controlled trial. Diabetes, obesity & metabolism 2015;17(10):936-48. [DOI: 10.1111/dom.12503]

Rosenstock 2015a

Rosenstock J, Hansen L, Zee P, Li Y, Cook W, Hirshberg B, et al. Dual add-on therapy in type 2 diabetes poorly controlled with metformin monotherapy: a randomized double-blind trial of saxagliptin plus dapagliflozin addition versus single addition of saxagliptin or dapagliflozin to metformin. Diabetes care 2015;38(3):376-83. [PubMed: 25352655]

Ross 2015

Ross S, Thamer C, Cescutti J, Meinicke T, Woerle H J, Broedl U C. Efficacy and safety of empagliflozin twice daily versus once daily in patients with type 2 diabetes inadequately controlled on metformin: a 16-week, randomized, placebo-controlled trial. Diabetes, obesity & metabolism 2015;17:699-702.

Schernthaner 2013

Schernthaner G, Gross JL, Rosenstock J, Guarisco M, Fu M, Yee J, et al. Canagliflozin compared with sitagliptin for patients with type 2 diabetes who do not have adequate glycemic control with metformin plus sulfonylurea: a 52-week randomized trial. Diabetes care 2013;36(9):2508-15. [Other: 1935-5548]

Karla Childers on behalf of Janssen Pharmaceuticals, Inc, a pharmaceutical company of Johnson & Johnson. Additional data: HDL and triglyceride absolute changes of ALAT, LDL, and serum creatinine. Overall adverse events (all types) tables by System Organ Class (SOC), other adverse events including fractures. The YODA-project Email communication on December 12 2014.

Stenløf 2013

* Stenlöf K, Cefalu WT, Kim KA, Alba M, Usiskin K, Tong C, et al. Efficacy and safety of canagliflozin monotherapy in subjects with type 2 diabetes mellitus inadequately controlled with diet and exercise. Diabetes, obesity & metabolism 2013;15(4):372-82. [Other: 1463-1326]

Stenlof K, Cefalu WT, Kim KA, Jodar E, Alba M, Edwards R, et al. Long-term efficacy and safety of canagliflozin monotherapy in patients with type 2 diabetes inadequately controlled with diet and exercise: findings from the 52-week CANTATA-M study. Current medical research and opinion 2014;30(2):163-75. [PubMed: 24073995]

Karla Childers on behalf of Janssen Pharmaceuticals, Inc, a pharmaceutical company of Johnson & Johnson. Additional data: HDL and triglyceride absolute changes of ALAT, LDL, and serum creatinine. Overall adverse events (all types) tables by System Organ Class (SOC), other adverse events including fractures. The YODA-project Email communication on December 12 2014.

Strojek 2011

Strojek K, Yoon KH, Hruba V, Elze M, Langkilde AM, Parikh S. Effect of dapagliflozin in patients with type 2 diabetes who have inadequate glycaemic control with glimepiride: a randomized, 24-week, double-blind, placebo-controlled trial. Diabetes, obesity & metabolism 2011;13(10):928-38. [Other: 1463-1326]

* Strojek K, Yoon KH, Hruba V, Sugg J, Langkilde AM, Parikh S. Dapagliflozin added to glimepiride in patients with type 2 diabetes mellitus sustains glycemic control and weight loss over 48 weeks: a randomized, double-blind, parallel-group, placebo-controlled trial. Diabetes therapy: research, treatment and education of diabetes and related disorders 2014;5(1):267-83. [PubMed: 24920277]

Wilding 2009

* Wilding JPH, Norwood P, T'Joen C, Bastien A, List JF, Fiedorek FT. A study of dapagliflozin in patients with type 2 diabetes receiving high doses of insulin plus insulin sensitizers: applicability of a novel insulin-independent treatment. Diabetes care 2009;32(9):1656-62. [Other: 1935-5548]

Kyle Nahrebne on behalf of AstraZeneca Pharmaceuticals. Additional data CSRs: LDL given in percentage change from baseline, ALAT, serum creatinine, heart rate, systolic and diastolic blood pressure. Overall adverse events (all types) tables by System Organ Class (SOC), other adverse events including fractures. Email communication on July 7 2014.

Wilding 2012

Wilding JP, Woo V, Rohwedder K, Sugg J, Parikh S. Dapagliflozin in patients with type 2 diabetes receiving high doses of insulin: efficacy and safety over 2 years. Diabetes, obesity & metabolism 2014;16(2):124-36. [1463-1326: (Electronic)]

* Wilding JPH, Woo V, Soler NG, Pahor A, Sugg J, Rohwedder K, et al. Long-term efficacy of dapagliflozin in patients with type 2 diabetes mellitus receiving high doses of insulin: a randomized trial. Annals of internal medicine 2012;156(6):405-15. [Other: 1539-3704]

Kyle Nahrebne on behalf of AstraZeneca Pharmaceuticals. Additional data CSRs: LDL given in percentage change from baseline, ALAT, serum creatinine, heart rate, systolic and diastolic blood pressure. Overall adverse events (all types) tables by System Organ Class (SOC), other adverse events including fractures. Email communication on July 7 2014.

Wilding 2013

* Wilding JPH, Charpentier G, Hollander P, González-Gálvez G, Mathieu C, Vercruysse F, et al. Efficacy and safety of canagliflozin in patients with type 2 diabetes mellitus inadequately controlled with metformin and sulphonylurea: a randomised trial. International journal of clinical practice 2013. [Other: 1742-1241]

Karla Childers on behalf of Janssen Pharmaceuticals, Inc, a pharmaceutical company of Johnson & Johnson. Additional data: HDL and triglyceride absolute changes of ALAT, LDL, and serum creatinine. Overall adverse events (all types) tables by System Organ Class (SOC), other adverse events including fractures. The YODA-project Email communication on December 12 2014.

Excluded studies

Araki 2015

Araki E, Tanizawa Y, Tanaka Y, Taniguchi A, Koiwai K, Kim G, et al. Long-term treatment with empagliflozin as add-on to oral antidiabetes therapy in Japanese patients with type 2 diabetes mellitus. Diabetes, obesity & metabolism 2015;17:665-74.

Bailey 2012

Bailey CJ, Iqbal N, T'Joen C, List JF. Dapagliflozin monotherapy in drug-naive patients with diabetes: a randomized-controlled trial of low-dose range. Diabetes, obesity & metabolism

2012;14(10):951-9. [1463-1326: (Electronic)]

Bailey 2015

Bailey CJ, Morales Villegas EC, Woo V, Tang W, Ptaszynska A, List JF. Efficacy and safety of dapagliflozin monotherapy in people with Type 2 diabetes: a randomized double-blind placebo-controlled 102-week trial. Diabetic medicine: a journal of the British Diabetic Association 2015;32(4):531-41. [Other: NCT00528372]

Barnett 2014

Barnett AH, Mithal A, Manassie J, Jones R, Rattunde H, Woerle HJ, et al. Efficacy and safety of empagliflozin added to existing antidiabetes treatment in patients with type 2 diabetes

and chronic kidney disease: a randomised, double-blind, placebo-controlled trial. The Lancet. Diabetes & endocrinology 2014;2(5):369-84. [2213-8595: (Electronic)]

Ferrannini 2013b

Ferrannini E, Berk A, Hantel S, Pinnetti S, Hach T, Woerle HJ, et al. Long-term safety and efficacy of empagliflozin, sitagliptin, and metformin: an active-controlled, parallel-group, randomized, 78-week open-label extension study in patients with type 2 diabetes. Diabetes care 2013;36(12):4015-21. [PubMed: 24186878]

Fulcher 2015

Fulcher G, Matthews D R, Perkovic V, de Zeeuw D, Mahaffey K W, Weiss R, et al. Efficacy and safety of canagliflozin used in conjunction with sulfonylurea in patients with Type 2 Diabetes Mellitus: a randomized, controlled trial. Diabetes therapy: research, treatment and education of diabetes and related disorders 2015;6:289-302.

Inagaki 2014

Inagaki N, Kondo K, Yoshinari T, Takahashi N, Susuta Y, Kuki H. Efficacy and safety of canagliflozin monotherapy in Japanese patients with type 2 diabetes inadequately controlled with diet and exercise: a 24-week, randomized, double-blind, placebo-controlled, Phase III study. Expert opinion on pharmacotherapy 2014;15(11):1501-15. [1744-7666: (Electronic)]

Kadowaki 2015

Kadowaki Takashi, Haneda Masakazu, Inagaki Nobuya, Terauchi Yasuo, Taniguchi Atsushi, Koiwai Kazuki, et al. Efficacy and safety of empagliflozin monotherapy for 52 weeks in japanese patients with type 2 diabetes: a randomized, double-blind, parallel-group study. Advances in Therapy 2015;32:306-18.

Kohan 2013

Kohan DE, Fioretto P, Tang W, List JF. Long-term study of patients with type 2 diabetes and moderate renal impairment shows that dapagliflozin reduces weight and blood pressure but does not improve glycemic control. Kidney international 2013. [Other: 1523-1755]

Ljunggren 2012

Ljunggren O, Bolinder J, Johansson L, Wilding J, Langkilde AM, Sjostrom CD, et al. Dapagliflozin has no effect on markers of bone formation and resorption or bone mineral density in patients with inadequately controlled type 2 diabetes mellitus on metformin. Diabetes, obesity & metabolism 2012;14(11):990-9. [PubMed: 22651373]

Matthaei 2015a

Matthaei S, Catrinoiu D, Celinski A, Ekholm E, Cook W, Hirshberg B, et al. A randomized, double-blind trial of triple therapy with saxagliptin add-on to dapagliflozin plus metformin in patients with type 2 diabetes. Diabetes Care 2015. [DOI: DOI: 10.2337/dc15-0811]

NCT01294423

* Astra Zeneca. A 24-week randomised, double-blind, parallel-group, multi-centre, placebo-controlled phase III trial to evaluate the efficacy and safety of dapagliflozin as monotherapy in Japanese subjects with type 2 diabetes who have inadequate glycemic control with diet and exercise [Evaluate efficacy and safety in Japanese subjects with type 2 diabetes mellitus]. Astra Zeneca 2013 D1692C00006 Accessed September 2015.

Neal 2015

Neal B, Perkovic V, de Zeeuw D, Mahaffey KW, Fulcher G, Ways K, et al. Efficacy and safety of canagliflozin, an inhibitor of sodium-glucose cotransporter 2, when used in conjunction with insulin therapy in patients with type 2 diabetes. Diabetes care 2015;38(3):403-11. [PubMed: 25468945]

Schumm 2015

Astra Zeneca. A 16-week, multicentre, randomised, double-blind, placebo-controlled phase iii study to evaluate the safety and efficacy of dapagliflozin 2.5 mg bid, 5 mg bid and 10 mg qd versus placebo in patients with type 2 diabetes who are inadequately controlled on metformin-ir monotherapy. NCT01217892 Astra Zenenca 2012 D1691C00003 accessed September 2015.

* Schumm-Draeger PM, Burgess L, Korányi L, Hruba V, Hamer-Maansson JE, de Bruin TW. Twice-daily dapagliflozin co-administered with metformin in type 2 diabetes: a 16-week randomized, placebo-controlled clinical trial. Diabetes, obesity & metabolism 2015;17(1):42-51. [DOI: 10.1111/dom.12387]

Stenløf 2014

Stenlof K, Cefalu WT, Kim KA, Jodar E, Alba M, Edwards R, et al. Long-term efficacy and safety of canagliflozin monotherapy in patients with type 2 diabetes inadequately controlled with diet and exercise: findings from the 52-week CANTATA-M study. Current medical research and opinion 2014;30(2):163-75. [PubMed: 24073995]

Wilding 2014

Wilding JP, Woo V, Rohwedder K, Sugg J, Parikh S. Dapagliflozin in patients with type 2 diabetes receiving high doses of insulin: efficacy and safety over 2 years. Diabetes Obes Metab 2014;16(2):124-36. [1463-1326: (Electronic)]

Yale 2013

Yale JF, Bakris G, Cariou B, Yue D, David-Neto E, Xi L, et al. Efficacy and safety of canagliflozin in subjects with type 2 diabetes and chronic kidney disease. Diabetes, obesity & metabolism 2013;15(5):463-73. [Other: 1463-1326]
