# Supplementary material for: Benefits and Harms of Sodium-Glucose Co-Transporter 2 Inhibitors in Patients with Type 2 Diabetes: A Systematic Review and Meta-Analysis
Source: PLoS One. 2016 Nov 11;11(11):e0166125. doi: 10.1371/journal.pone.0166125 (PMC5106000; doi:10.1371/journal.pone.0166125)
Supplement: S3 File — (PDF) [file pone.0166125.s005.pdf]

# PRISMA 2009 Checklist

| Section/topic | # | Checklist item | Reported on page # |
|---|---|---|---|
| **TITLE** | | | |
| Title | 1 | Identify the report as a systematic review, meta-analysis, or both. | Title: Benefits and harms of sodium-glucose co-transporter 2 inhibitors in patients with type 2 diabetes: a systematic review and meta-analysis<br><br>page 1 |
| **ABSTRACT** | | | |
| Structured summary | 2 | Provide a structured summary including, as applicable: background; objectives; data sources; study eligibility criteria, participants, and interventions; study appraisal and synthesis methods; results; limitations; conclusions and implications of key findings; systematic review registration number. | Abstract,<br>PROSPERO CRD42014008960<br>page 2 |
| **INTRODUCTION** | | | |
| Rationale | 3 | Describe the rationale for the review in the context of what is already known. | Introduction,<br>page 4-5 |
| Objectives | 4 | Provide an explicit statement of questions being addressed with reference to participants, interventions, comparisons, outcomes, and study design (PICOS). | Introduction page 4-5<br>Full PICO statement of inclusion criteria in Methods, 'Trial eligibility and selection' page 6 |
| **METHODS** | | | |
| Protocol and registration | 5 | Indicate if a review protocol exists, if and where it can be accessed (e.g., Web address), and, if available, provide registration information including registration number. | (PROSPERO CRD42014008960).<br>Storgaard H, Gluud LL, Christensen M, Knop FK, Vilsboll T. The effects of sodium-glucose co-transporter 2 inhibitors in patients with type 2 diabetes: protocol for a systematic review with meta-analysis of randomised trials. BMJ Open. 2014;4(8):e005378. PubMed PMID: 25232561; PubMed Central PMCID: PMC4139650. |
| Eligibility criteria | 6 | Specify study characteristics (e.g., PICOS, length of follow-up) and report characteristics (e.g., years considered, language, publication status) used as criteria for eligibility, giving rationale. | Methods 'trial eligibility and selection'<br><br>"Published, English language RCTs, conducted in adult patients (at least 18 years of age) and lasted for at least 12 weeks to permit assessment the effect of SGLT2-i on HbA1c. The intervention comparisons comprised SGLT2-i (dapagliflozin, canagliflozin and empagliflozin) versus placebo, or other oral antidiabetic drugs (OAD) at doses that are currently |

| | | | |
|---|---|---|---|
| | | | recommended by FDA and/or EMA as a maximum daily dose i.e. canagliflozin 300 mg; dapagliflozin 10 mg; empagliflozin 25 mg. Co-interventions with other antidiabetic agents were allowed if administered to the intervention and control groups).<br><br>We excluded studies which involved participants with impaired kidney function, as reduced glomerular filtration rate reduces the glucosuria and thereby the glucose-lowering effect of the SGLT2-i."<br><br>Page 6 |
| Information sources | 7 | Describe all information sources (e.g., databases with dates of coverage, contact with study authors to identify additional studies) in the search and date last searched. | Methods<br>Search methods for identification of studies<br>Cochrane Library, MEDLINE, EMBASE, the Science Citation Index and the WHO Trial Search Database<br>We obtained additional data on e.g. heart rate, ALT and lipids from the study investigators, the manufacturers and the YODA-project (details listed in S1 Appendix). Page 6 |
| Search | 8 | Present full electronic search strategy for at least one database, including any limits used, such that it could be repeated. | Page 6 |
| Study selection | 9 | State the process for selecting studies (i.e., screening, eligibility, included in systematic review, and, if applicable, included in the meta-analysis). | Trial selection was carried out by two review authors who independently reviewed the search results and selected trials for inclusion, with involvement of a third review author (CB or TV) if necessary to resolve disagreements.<br>Page 7 |
| Data collection process | 10 | Describe method of data extraction from reports (e.g., piloted forms, independently, in duplicate) and any processes for obtaining and confirming data from investigators. | Trial characteristics were recorded and two authors (HS and MG) identified outcomes from each included study and extracted outcome data into extraction forms (Excel spreadsheets); consensus was reached through discussion.<br><br>Page 7<br><br>We obtained additional data on e.g. heart rate, ALT and lipids from the study investigators, the manufacturers and the YODA-project |
| Data items | 11 | List and define all variables for which data were sought (e.g., PICOS, funding sources) and any assumptions and simplifications made. | All variables, for all included studies provided in the S1 Table 'Characteristics of Included Studies'<br>For trials presenting data from more than one treatment period (e.g., 26 and 52 weeks), data from the longest treatment period were used.<br>Trial characteristics (including methods, participants, interventions, study outcomes, |

| | | | |
|---|---|---|---|
| | | | potential risks of bias and funding source)<br>Page 7-8 |
| Risk of bias in individual studies | 12 | Describe methods used for assessing risk of bias of individual studies (including specification of whether this was done at the study or outcome level), and how this information is to be used in any data synthesis. | Cochrane Collaboration's risk of bias assessment tool used.<br><br>Page 8 |
| Summary measures | 13 | State the principal summary measures (e.g., risk ratio, difference in means). | For continuous outcomes, we estimated the mean differences (MDs) between groups. We present dichotomous outcomes data as risk ratios (RRs).<br>Page 8 |
| Synthesis of results | 14 | Describe the methods of handling data and combining results of studies, if done, including measures of consistency (e.g., $I^2$) for each meta-analysis. | Methods<br><br>'Data synthesis'<br><br>We undertook meta-analyses (intention-to-treat analyses including all patients randomized were performed where possible) in RevMan using the Mantel-Haenszel test with a random effects model, unless stated otherwise, and present these with 95% confidence intervals (CI) and standard deviations. For continuous outcomes, we estimated the mean differences (MDs) between groups. We present dichotomous outcomes data as risk ratios (RRs). In all cases, if the calculated effect size was statistically significant (P value < 0.05), we state whether the result favored the intervention group or the control condition. For effect sizes of MD, values greater than 0.70 have been treated as large; values between 0.40 and 0.70 as moderate; and values less than 0.40 but greater than 0.10 as small.<br><br>Differences between subgroups were reported using tests for subgroup differences expressed as P values. $I^2$ values were was used as a measure of heterogeneity and are reported they exceeded 30%.<br><br>Page 8 |



| Section/topic | # | Checklist item | Reported on page # |
|---|---|---|---|
| Risk of bias across studies | 15 | Specify any assessment of risk of bias that may affect the cumulative evidence (e.g., publication bias, selective reporting within studies). | The bias risk assessment followed the using the Cochrane Collaboration's risk of bias assessment tool . In each domain, studies were given a rating of low, unclear or high risk. We used the Grades of Recommendation, Assessment, Development and Evaluation (GRADE) system to describe the quality of the evidence and the strength of recommendation, 'high' to |

| | | | |
|---|---|---|---|
| | | | 'very low'.<br><br>Page 8 |
| Additional analyses | 16 | Describe methods of additional analyses (e.g., sensitivity or subgroup analyses, meta-regression), if done, indicating which were pre-specified. | We conducted subgroup analysis on the basis of SGLT2-i type (canagliflozin, dapagliflozin, empagliflozin), and on the basis of OAD type (metformin, SU, DPP4-i).<br>Page 8 |
| **RESULTS** | | | |
| Study selection | 17 | Give numbers of studies screened, assessed for eligibility, and included in the review, with reasons for exclusions at each stage, ideally with a flow diagram. | "We identified 1050 records in total through our electronic database searches up to October 2013. An update search in October 2015 found 37 potential new studies; 25 were discarded as they did not meet the inclusion criteria. Thus we have included 42 studies (59 of reports of studies) (Fig. 1) in this review comprising 24,500 randomized participants in total (please see S1 Appendix.docx). The studies compared different doses of SGLT2-i versus placebo or another other oral-antidiabetic drug. Four studies were multi-arm, comparing SGLT2-i versus placebo and versus OAD" Page 9 and PRISMA flowchart figure 1 |
| Study characteristics | 18 | For each study, present characteristics for which data were extracted (e.g., study size, PICOS, follow-up period) and provide the citations. | Summarised in results 'description of studies' page 9-10<br>Table 1, and in S1 table. |
| Risk of bias within studies | 19 | Present data on risk of bias of each study and, if available, any outcome level assessment (see item 12). | Page 14-15 and S1 figure, S2 figure |
| Results of individual studies | 20 | For all outcomes considered (benefits or harms), present, for each study: (a) simple summary data for each intervention group (b) effect estimates and confidence intervals, ideally with a forest plot. | Results, page 11-14<br>Figure 2 and 3, Tables 2, 3 and S2 Table |
| Synthesis of results | 21 | Present results of each meta-analysis done, including confidence intervals and measures of consistency. | Results, page 11-14<br>Figure 2 and Fig 3.<br>Tables 2, 3 and S2 Table |
| Risk of bias across studies | 22 | Present results of any assessment of risk of bias across studies (see Item 15). | Two supplementary items are provided in addition to the main text. Figure S1 and figure S2. |
| Additional analysis | 23 | Give results of additional analyses, if done (e.g., sensitivity or subgroup analyses, meta-regression [see Item 16]). | See subgroup analyses, presented with primary and secondary outcome findings. |
| **DISCUSSION** | | | |

| | | | |
|---|---|---|---|
| Summary of evidence | 24 | Summarize the main findings including the strength of evidence for each main outcome; consider their relevance to key groups (e.g., healthcare providers, users, and policy makers). | Discussion, page 15<br>conclusions page 19<br>"The highest approved doses of canagliflozin, dapagliflozin and empagliflozin compared with placebo, were effective in reducing HbA1c in patients with type 2 diabetes. In spite of the large number of RCTs with a low risk of bias in several domains, we downgraded the evidence to low quality. Based on our assessment of publication bias and other smalls study effects, we found evidence of bias and therefore a risk that the analyses overestimate the intervention benefit. In the included RCTs, SGLT2-i had no discernible beneficial or harmful effects on serious adverse events including mortality, cancer, ketoacidosis, severe hypoglycaemia, bladder cancer, breast cancer or other cancer types. SGLT2-i also had no effect on CVD events, but SGLT2-i were associated a beneficial effect on CVD-associated risk factors including body weight, blood pressure and lipids (although elevations in LDL lipids may be a concern). As expected, SGLT2-i increased the risk of non-serious adverse events, including serum creatinine levels, UTI and GTI. Additional meta-analyses showed similar effects, when comparing SGLT2-i versus other OAD, but the analyses with active comparators included a smaller number of trials and patients. We also identified important potential limitations, which mainly included a high degree of inconsistency. The inconsistency is likely to reflect clinical heterogeneity in terms of the interventions, populations and follow-up times. Furthermore, selective reporting of outcomes (e.g. CVD, cancer etc.) may also bias the estimates. Therefore, it is possible that the true effect differs somewhat from the estimated effects". |
| Limitations | 25 | Discuss limitations at study and outcome level (e.g., risk of bias), and at review-level (e.g., incomplete retrieval of identified research, reporting bias). | Discussion page 16 "However, none of the trials compared the individual SGLT2-is and the results, therefore, remain exploratory. Thus, the lack of head-to-head comparisons between the SGLT2-i means that we cannot exclude the possibility that the difference between SGLT2-i reflect patient inclusion criteria rather than a true difference between intervention effects." |
| Conclusions | 26 | Provide a general interpretation of the results in the context of other evidence, and implications for future research. | Discussion page 18<br>Future research: discussion "Future RCTs would ideally be long-lasting and large-scale comparing SGLT2-i with placebo or existing therapies. Such RCTs should additionally include reporting of serious adverse events such as CVD risk, renal safety, ketoacidosis and severe hypoglycaemia with adequate follow-up (over one year), to establish the long-term consequences of SGLT2-i therapy." Page 17-18 |
| **FUNDING** | | | |
| Funding | 27 | Describe sources of funding for the systematic review and other support (e.g., supply of data); role of funders for the systematic review. | Declarations of interest<br>"Funding: The research did not receive specific grant from any funding agency in the public, commercial or not-for-profit sectors. No sponsor was involved in study design, and no sponsor had authority in collection, management, analysis and interpretation of data." |

| | | | |
|---|---|---|---|
| | | | Page 19 |

*From:* Moher D, Liberati A, Tetzlaff J, Altman DG, The PRISMA Group (2009). Preferred Reporting Items for Systematic Reviews and Meta-Analyses: The PRISMA Statement. PLoS Med 6(6): e1000097. doi:10.1371/journal.pmed1000097

For more information, visit: **www.prisma-statement.org**.
