# Supplementary material for: Benefits and Harms of Sodium-Glucose Co-Transporter 2 Inhibitors in Patients with Type 2 Diabetes: A Systematic Review and Meta-Analysis
Source: PLoS One. 2016 Nov 11;11(11):e0166125. doi: 10.1371/journal.pone.0166125 (PMC5106000; doi:10.1371/journal.pone.0166125)
Supplement: S1 Table — (PDF) [file pone.0166125.s006.pdf]

S1 Table. Characteristics of included studies

Bailey 2010

**Methods**	24-week phase 3, multicenter, randomized, placebo-controlled, double-blind, parallel-group trial in 80 sites (30 in the USA, 21 in Canada, 11 in Argentina, ten in Mexico, and eight in Brazil).
**Participants**	546 initially randomized: dapagliflozin 2·5 mg n=137; dapagliflozin 5 mg n= 137; dapagliflozin 10 mg n=135; placebo n=137.
**Interventions**	Dapagliflozin 2·5 mg; dapagliflozin 5 mg; dapagliflozin 5 mg n=135; placebo n=137.<br>Randomly assigned (in a 1:1:1:1 ratio) by the IVRS to double-blinded groups of once-daily dapagliflozin 2·5 mg, 5 mg, or 10 mg, or matching placebo given orally before the morning meal for 24 weeks.<br>Add on: metformin open-label 500 mg metformin, diet and exercise counselling.
**Outcomes**	Trial extended to 102 weeks, outcome data derived from 102 weeks.<br>**Primary outcomes of the trial**<br>change in HbA1c levels from baseline at week 102.<br>**Secondary outcomes of the trial**<br>change from baseline in fasting plasma glucose (FPG) level<br>percent change from baseline in body weight change and decreases in bodyweight of 5% or more.<br>changes in fasting plasma glucose concentration and total bodyweight at week 24,<br>change in fasting plasma glucose concentration at week 1,<br>the proportion of patients achieving a therapeutic glycemic response (HbA1c <7% at week 24)<br>lipids; total cholesterol, LDL cholesterol.<br>**Adverse events and safety assessments**<br>adverse events (AEs) were reported throughout the study,<br>urinary tract infection, genital infection.<br>Serious adverse events defined as:<br>fatal, life threatening,

	required admission to hospital,<br>prolonged an existing hospital stay,<br>resulted in persistent or significant disability or incapacity,<br>cancer or a congenital anomaly,<br>resulted in development of drug dependency or drug abuse,<br>medical event that jeopardized the patient or required intervention to prevent a serious outcome.
**Inclusion criteria**	men and women,<br>T2DM,<br>aged 18–77 years,<br>with inadequate glycemic control (HbA1c levels 7–10%),<br>C-peptide concentration 0·34 nmol/L or more,<br>body-mass index 45 kg/m² or less,<br>taking a stable dose of metformin (≥1500 mg per day) for at least 8 weeks before enrolment.
**Exclusion criteria**	serum creatinine 133 µmol/L or more for men or 124 µmol/L or more for women (consistent with metformin labelling),<br>urine albumin/creatinine ratio more than 203·4 mg/mmol,<br>aspartate aminotransferase or alanine aminotransferase more than three times the upper limit of normal,<br>creatine kinase more than three times the upper limit of normal,<br>symptoms of poorly controlled diabetes (including marked polyuria and polydipsia with >10% weight loss during the 3 months before enrolment),<br>clinically significant renal, hepatic, hematological, oncological, endocrine, psychiatric, or rheumatic disease; recent cardiovascular event (within 6 months) or New York Heart Association class III or IV congestive heart failure,<br>systolic blood pressure 180 mm Hg or more or diastolic blood pressure 110 mm Hg or more.
**Notes**	ClinicalTrials.gov identifier: NCT00528879<br><br>Background treatment metformin. 2010, 24 weeks. Bailey 2013 is a 78-week extension of Bailey 2010. We use outcome data from 102 weeks in our analyses.

Risk of bias table

**Bias**	**Authors' judgement**	**Support for judgement**
Random sequence	Low risk	Random numbers by central interactive voice response

generation (selection bias)		system. Site was assigned a block of random patient treatment assignments when calling to randomize the site's first patient.
Allocation concealment (selection bias)	Low risk	Randomization schedules were computer-generated by the sponsor and stored in a secure location.
Blinding of participants and personnel (performance bias)	Low risk	The patients, investigators, and sponsor personnel were blinded to treatment allocation and HbA1c and urinary glucose concentrations. The film-coated placebo and active tablets were similar in color, shape, size, texture, and taste.
Blinding of outcome assessment (detection bias)	Low risk	The patients, investigators, and sponsor personnel were blinded to treatment allocation and HbA1c and urinary glucose concentrations. The film-coated placebo and active tablets were similar in color, shape, size, texture, and taste.
Incomplete outcome data (attrition bias)	Low risk	483/546 completed. Data set comprised n=534: i.e. all randomized patients who received at least one dose of double-blind study medication and who had both a baseline and at<br>least one post-baseline measurement. Last observation carried forward, drops and losses to follow up all accounted for.
Selective reporting (reporting bias)	Low risk	All clinically relevant outcomes were defined and reported.
Other bias	Unclear risk	The sponsors of the study were involved in study design, data collection, data review, and data analysis, and were responsible for gathering all data from investigation centers for the clinical database. The report was prepared by the authors, with contributions from the sponsor Bristol-Myers Squibb.

Bode 2013

**Methods**	26 week (extended to 104 weeks), randomized, double-blind, placebo-controlled, phase 3 study in 90 study centers in 17 countries
**Participants**	714 initially randomized: canagliflozin 100mg n=241; canagliflozin 300mg n=236; placebo n=237.

**Interventions**	Canagliflozin 300mg or 300mg versus placebo for up to 104 weeks.<br><br>Participants were randomized in a I:I:I ratio to canagliflozin 100 mg or 300 mg or placebo once daily before the first meal of the day as add-on therapy to their ongoing stable T2DM treatment regimen.<br><br>Add on: metformin, alfaglucosidase, DPP-4, GLP1-a, insulin, pioglitazone, SU.
**Outcomes**	**Primary outcomes of the trial**<br><br>change in HbA1c levels from baseline to week 26, outcome data in our analyses are derived from the 104-week time point (extension study).<br><br>**Secondary outcomes of the trial**<br><br>change from baseline in fasting plasma glucose (FPG) level,<br>systolic blood pressure (BP),<br>percent change from baseline in body weight,<br>triglyceride levels,<br>high-density lipoprotein cholesterol (HDL-C) level,<br>proportion reaching HbA1c levels < 7.0%.<br><br>**Adverse events and safety assessments**<br><br>adverse events,<br>safety laboratory tests,<br>vital sign measurements,<br>physical examinations and 12-lead electrocardiograms,<br>osmotic diuresis and volume depletion,<br>urinary tract infections (UTIs),<br>genital mycotic infections.
**Inclusion criteria**	men and women<br>T2DM<br>aged 55 to 80 years, (older adults)<br>BMI between 20 and 40 kg/m ^{2},<br>FPG level < 270 mg/dL (15.0 mmol/L) at start of the single-blind, placebo run-in period),<br>fasting fingerstick blood glucose level 2: 110 mg/dL (6.1 mmol/L) and < 270 mg/dL (15.0 mmol/L) at baseline.<br>with inadequate glycemic control (HbA1c levels ≥7.0%)<br>on no blood glucose-lowering agent;<br>or on a stable regimen of blood glucose- lowering agent(s) as monotherapy or combination therapy (including metformin, sulfonylurea, dipeptidyl peptidase-4 (DPP-4) inhibitor, a-

	glucosidase inhibitor, glucagon-like peptide-I (GLP-I) agonist, or insulin for 2: 12 weeks prior to screening) or pioglitazone for 2: 6 months prior to screening)) used in accordance with local prescribing information.
**Exclusion criteria**	type I diabetes mellitus; repeated FPG level 2: 270 mg/dL (15.0 mmol/L) during the pretreatment phase,<br>history of myocardial infarction, unstable angina, revascularization procedure, or cerebrovascular accident within 3 months before screening; history of New York Heart Association Class II I- IV cardiac disease,<br>uncontrolled hypertension;<br>eGFR <50 mL/min/1.73 m^2<br>if on metformin therapy, excluded if serum creatinine levels ≥1.4 mg/dL (124 µmol/L for men and ≥1.3 mg/dL (115 µmol/L for women or any contraindication to the use of metformin (including low eGFR).
**Notes**	ClinicalTrials.gov identifier: NCT01106651<br><br>Followed by a 78-week, placebo-controlled, double-blind extension period and a 30-day follow-up period (Bode 2015), data from which is included in our analyses.

Risk of bias table

**Bias**	**Authors' judgement**	**Support for judgement**
Random sequence generation (selection bias)	Low risk	Randomization was performed using an interactive voice response system/interactive web response system with a computer-generated randomization schedule prepared by the sponsor before the study. Randomization carried out in permuted blocks of 6 stratified based on T score of lumbar spine.
Allocation concealment (selection bias)	Unclear risk	Not described but implied as randomization was performed using an interactive voice response system/interactive web response system with a computer-generated randomization schedule prepared by the sponsor before the study.
Blinding of participants and personnel	Low risk	Participants, investigators, and local sponsor personnel were to remain blinded to treatment assignment until the final database lock.

(performance bias)		(To maintain blinding after randomization, HbA1c and FPG levels were masked to the study centers unless these values met prespecified glycemic criteria for the initiation of rescue medication or after glycemic rescue medication was started).
Blinding of outcome assessment (detection bias)	Low risk	Participants, investigators, and local sponsor personnel were to remain blinded to treatment assignment until the final database lock.
Incomplete outcome data (attrition bias)	Low risk	Completers at 26 weeks: 197/237 placebo, 226/241 100mg canagliflozin, 209/236 300mg canagliflozin. Completers at 78 weeks: 158/237 placebo, 184/241 100mg canagliflozin, 178/236 300mg canagliflozin.<br><br>Primary and secondary efficacy analyses were performed using the modified intent-to-treat (m ITT) population, which consisted of all randomized participants who took at least one dose of double-blind study drug (n=714). Drop outs and losses to follow up accounted for.<br><br>Study report describes some limitations, in that placebo participants may have dropped out due to lack of glycemic control and too few >75 years participants were included in the trial.
Selective reporting (reporting bias)	Low risk	All major and clinically relevant outcomes were reported. Minor discrepancies between outcome data in main study reports and supplementary. Comment: not though to introduce a source of bias.
Other bias	Unclear risk	Data-analysis and editorial support was provided by pharmaceutical company support, in addition to trial being carried out by pharmaceutical company.

Bolinder 2012

**Methods**	24-week, multicenter, randomized, parallel-group, double-blind, placebo-controlled phase 3 study at 40 sites in 5 countries (Bulgaria, Czech Republic, Hungary, Poland, and Sweden).
**Participants**	446 initially randomized (314 to main assessment, 132 to sub study): dapagliflozin 10mg n=91; placebo n=91.

**Interventions**	Patients were randomized in a 1:1 ratio to double-blind treatment with either dapagliflozin 10 mg or placebo for 24 weeks extended to 102 weeks.<br><br>Add on: metformin.<br><br>Rescue therapy permitted.
**Outcomes**	At 24 weeks:<br><br>**Primary outcomes of the trial**<br><br>change from baseline at week 24 in total body weight (TBW)<br><br>**Secondary outcomes of the trial**<br><br>change from baseline in waist circumference,<br>total FM as measured by DXA<br>proportion of patients achieving a body weight reduction of at least 5% at week 24.<br>change from baseline 24 for total LM as measured by DXA,<br>glycemic variables<br>HbA1c<br>FPG,<br>adipose tissue markers adiponectin and leptin,<br>seated systolic and diastolic blood pressure.<br>MR assessments were conducted in 80 in the sub study: change from baseline at week 24 for visceral adipose tissue (VAT) volume, adipose tissue (SAT) volume, and hepatic lipid (HL) content.<br><br>**Adverse events and safety assessments**<br><br>hypoglycemic events,<br>laboratory tests,<br>electrocardiographic and physical examinations, and vital signs<br>genital infection<br>urinary tract infection (UTI)<br>renal impairment, renal failure, kidney stones,<br>hypotension/dehydration/hypovolemia<br>change from baseline in total BMD (grams per square centimeter)
**Inclusion criteria**	men and women,<br>T2DM,<br>women aged 55–75 years who were postmenopausal for a period of at least 5 years (or to have had an oophorectomy) for at least 5 years prior,) or men aged 30–75 years<br>hemoglobin A1c (HbA1c) 6.5–8.5%,

	fasting plasma glucose (FPG) less than or equal to 240 mg/dl (≤13.2 mmol/l); body mass index (BMI) of 25 kg/m2 or higher,<br>body weight no higher than 120 kg (due to limitations imposed by DXA equipment),<br>treatment exclusively with metformin at a stable dose of at least 1500 mg/d for at least 12 weeks before enrolment.
**Exclusion criteria**	**Exclusion criteria**<br><br>men under 30 years and perimenopausal women<br>poor glycemic control (HbA1c 8.5%)<br>BMD T-scores less than −2.0 at lumbar spine, femoral neck or total hip regions at the baseline DXA measurement<br>receiving treatments known to significantly influence bone metabolism (e.g. bisphosphonates, calcitonin, corticosteroids or hormone replacement therapy) were excluded.
**Notes**	ClinicalTrials.gov identifier: NCT00855166<br><br>Bolinder 2012 is 78-week extension of Bolinder 2012.

Risk of bias table

**Bias**	**Authors' judgement**	**Support for judgement**
Random sequence generation (selection bias)	Low risk	Patients were allocated to study treatments according to a predefined computer-generated randomization scheme provided by AstraZeneca. Balanced block sizes of four to ensure approximately equal numbers of patients across the treatment groups and within each stratum (in two strata, men and women).
Allocation concealment (selection bias)	Low risk	No patients, investigators, or personnel at AstraZeneca or Bristol-Myers Squibb had access to the randomization codes during the 24-wk double-blind treatment period.
Blinding of participants and personnel (performance bias)	Low risk	Patients and investigators were blinded to study treatment. All investigational products (dapagliflozin 10 mg and matching placebo) were identical in appearance, smell, and taste and packaged into identical bottles.
Blinding of outcome assessment	Low risk	No patients, investigators, or personnel at AstraZeneca or Bristol-Myers Squibb had access to the randomization codes during the 24-wk double-blind treatment period.

(detection bias)		
Incomplete outcome data (attrition bias)	Low risk	169/182 completed.<br><br>Two analysis sets were defined: the safety analysis set, consisting of all patients who received at least one dose of investigational product, and the full analysis set, consisting of all randomized patients who received at least one dose of investigational product and who had both a baseline and at least one post-baseline efficacy value for at least one efficacy variable.<br><br>Primary, key secondary, and exploratory endpoints were analyzed using the full analysis set.<br><br>For glycemic variables, observations after initiation of rescue therapy were excluded from the analysis, with these and other missing values for glycemic and non-glycemic variables at week 24 replaced using the last observation carried forward (LOCF) method.<br><br>All drop outs and losses to follow up accounted for.
Selective reporting (reporting bias)	Low risk	Besides the major prespecified outcomes, a number of exploratory endpoints were assessed (but these were clinically relevant).
Other bias	Unclear risk	This study was sponsored by AstraZeneca and Bristol-Myers Squibb. Some of the trialists are employees of Bristol-Myers Squibb.

Cefalu 2013

**Methods**	52-week, randomized, double-blind, active-controlled, phase 3 non-inferiority trial at 157 centers in 19 countries with double-blind extension to 104 weeks
**Participants**	1452 initially randomized: canagliflozin 100mg n=483; canagliflozin 300mg n= 485; glimepiride n=482.
**Interventions**	Canagliflozin 100 mg or 300 mg, versus glimepiride (up-titrated to 6 mg or 8 mg per day), (1:1:1) orally once daily for 104 weeks.<br><br>Add on: insulin, metformin
**Outcomes**	Outcomes assessed at week 104.

	**Primary outcomes of the trial**<br><br>change in HbA1c from baseline to week 52 and week 104, with a non-inferiority margin of 0·3% for the comparison of each canagliflozin dose with glimepiride.<br><br>**Secondary outcomes of the trial**<br><br>proportion of participants achieving HbA1c <7·0% or 6·5%,<br>change in fasting plasma glucose<br>systolic and diastolic blood pressure<br>percentage change in fasting plasma lipids, including HDL cholesterol, triglycerides, LDL cholesterol, non-HDL cholesterol, and ratio of LDL cholesterol to HDL cholesterol.<br><br>**Adverse events and safety assessments**<br><br>safety with adverse event reports,<br>laboratory tests,<br>vital sign measurements, physical examinations,<br>self-monitored blood glucose,<br>12-lead electro cardiograms,<br>genital mycotic infections,<br>urinary tract infections.
**Inclusion criteria**	men and women with T2DM,<br>aged 18 to 80 years<br>with glycated hemoglobin A1c (HbA1c) of 7·0–9·5% on stable metformin<br>receiving stable metformin therapy (≥2000 mg per day or ≥1500 mg per day if unable to tolerate a higher dose) for at least 10 weeks.
**Exclusion criteria**	a history of more than one severe hypoglycemic episode (within 6 months)<br>repeated measurements of fasting plasma glucose or fasting self-monitored blood glucose, or both, of 15·0 mmol/L or more during the pretreatment phase,<br>EGFR <55 mL/min/1·73 m^2 (or <60 mL/min/1·73 m^2 if based on restriction of metformin use in local label),<br>serum creatinine concentrations of 124 µmol/L or more for men and 115 µmol/L or more for women,<br>on thiazolidinedione within 16 weeks before screening.
**Notes**	ClinicalTrials.gov identifier: NCT00968812

	The study consisted of a 2 week, single-blind, placebo run-in period and a 52 week, double-blind, core treatment period, followed by a 52 week, double-blind, extension period included in our analyses (Leiter 2015).

Risk of bias table

Bias	Authors' judgement	Support for judgement
Random sequence generation (selection bias)	Low risk	Participants were then randomly assigned, in a 1:1:1 ratio, by an interactive voice or web response system. The sponsor prepared the computer-generated randomization schedule before the study. Randomization was balanced with the use of permuted blocks of three patients per block and stratified by whether the patient was taking a stable, protocol specified dose of metformin before screening versus whether they had either undergone metformin dose adjustment or discontinued use of a second anti-hyperglycemic drug, or both, and by country.
Allocation concealment (selection bias)	Unclear risk	Not described but randomization was performed using an interactive voice response system.
Blinding of participants and personnel (performance bias)	Low risk	Double blind placebo. After randomization, HbA1c and fasting plasma glucose values were masked to staff at the study centers unless values met glycemic rescue criteria (and were subsequently provided unmasked). Patients, study investigators, and local sponsor personnel were masked to treatment assignment until final database lock. To maintain masked treatment, study drug was supplied in levels (levels one to five) to allow for masked increases and decreases of glimepiride throughout the double-blind treatment period.
Blinding of outcome assessment (detection bias)	Low risk	Double blind placebo. HbA1c and fasting plasma glucose values were masked to staff at the study centers unless values met glycemic rescue criteria (and were subsequently provided unmasked). Patients, study investigators, and local sponsor personnel were masked to treatment assignment until final database lock.
Incomplete	Low risk	1161/1452 completed. Drop outs and losses to follow up

outcome data (attrition bias)		accounted for. Analyses performed using intention to treat carried forward.
Selective reporting (reporting bias)	Low risk	All clinically relevant outcomes are defined and reported.
Other bias	Unclear risk	The sponsor of the study had a role in study design and conduct; data collection, analysis, and interpretation; and writing of the article.

Cefalu 2015

**Methods**	52-week, multicenter, randomized, parallel-group, double-blind, multicenter, placebo-controlled study in 5 counties/continents (Europe, Asia, USA, Canada, and Argentina).
**Participants**	922 initially randomized (to main assessment, to sub study): dapagliflozin 10mg n=455; placebo n=459.
**Interventions**	Patients were randomized in a 1:1 ratio to double-blind treatment with either dapagliflozin 10 mg or placebo for 24 weeks extended to 52 weeks.<br>Add on: pre-existing stable background treatment (e.g. insulin, metformin) other than rosiglitazone.<br>Rescue therapy permitted.
**Outcomes**	At 52 weeks:<br>**Primary outcomes of the trial**<br>mean change in HbA1c from baseline to week 24<br>the proportion of responders achieving a three-item end point of combined clinical benefit at week 24 (an absolute drop from baseline in HbA1c of ≥0.5% (5.5 mmol/mol), a relative drop of ≥3% for total BW, and an absolute drop of ≥3 mmHg from baseline in seated SBP.<br>**Secondary outcomes of the trial**<br>mean change in seated SBP from baseline (at weeks 8 and 24),<br>mean percent body weight<br>mean change in body weight from baseline;<br>proportion with baseline BMI of ≥27 kg/m2 with a ≥5% reduction in BW.<br>mean change in seated diastolic blood pressure (DBP);

	proportion with seated SBP of <130 mmHg in the group of patients with a baseline seated SBP of ≥130 mmHg;<br>mean change in HbA1c in patients with a baseline HbA1c mean change in body weight from baseline ≥8.0% (64mmol/mol) and an HbA1c≥9.0% (75 mmol/mol);<br>proportion achieving an HbA1c <7.0% (53 mmol/mol);<br>mean change in FPG at weeks 1 and 24;<br>proportion rescued for failing to maintain FPG/HbA1c below the prespecified rescue criteria at weeks 4, 8, 16, 24 and 52;<br>proportion of patients achieving a reduction in HbA1c of ≥0.5% (5.5 mmol/mol);<br>proportion achieving a reduction in seated SBP from baseline of ≥3 or ≥5 mmHg;<br>mean change in calculated average daily insulin dose in patients treated with insulin at baseline.<br><br>**Adverse events and safety assessments** (all patients who received one or more doses of randomized study medication and who provided safety records over the 52 weeks)<br><br>all safety and tolerability events including:<br>CV events,<br>laboratory values,<br>electrocardiogram results,<br>vital signs,<br>hypoglycemic events,<br>calculated creatinine clearance,<br>estimated glomerular filtration rate (eGFR),<br>physical examination findings.
**Inclusion criteria**	Men aged ≥45 years or women ≥50 years (not of childbearing potential) with type 2 diabetes,<br>on monotherapy or dual combination therapy with oral antidiabetic drugs (OADs), insulin therapy in combination with OADs, or insulin monotherapy on a daily basis for 8 weeks, were stable for at least 4 weeks before enrolment, and showed inadequate glycemic control (7.2% (55mmol/mol) ≤HbA1c ≤10.5% (91 mmol/mol)).<br>cerebrovascular disease, and hypertension eligible if treatment uninterruptedly on a daily basis in the last 4 weeks before enrolment.<br>hypertension was defined as prior physician-made diagnosis of essential hypertension, i.e., before screening, or treatment with two or more antihypertensive agents (diuretics, β-blockers, angiotensin-converting enzyme inhibitors, angiotensin receptor blockers, or calcium channel antagonists), with one of the agents

	started for lowering blood pressure (BP), or treatment with one antihypertensive agent and a past physician recording of a BP exceeding 130/80 mmHg.<br>cardiovascular (CV) disease was defined as coronary heart disease, coronary artery stenosis >50%, stroke or transient ischemic attack, or peripheral artery disease treated with revascularization (amputation was not accepted).
**Exclusion criteria**	**Exclusion criteria**<br><br>type 1 diabetes mellitus,<br>more than three oral antidiabetic medications,<br>fasting plasma glucose (FPG) >15 mmol/l at randomization,<br>diabetic ketoacidosis,<br>recent CV event,<br>systolic BP (SBP) ≥165 mmHg, diastolic BP (DBP) ≥100 mmHg,<br>congestive heart failure (CHF)<br>calculated creatinine clearance <60 ml/min,<br>severe hepatic insufficiency and/or significant abnormal liver function.
**Notes**	24-week trial with 28-week extension, therefore 52-week outcome data are used in our analyses (a 52-week long extension study is ongoing).<br><br>ClinicalTrials.gov identifier NCT01031680

Risk of bias table

**Bias**	**Authors' judgement**	**Support for judgement**
Random sequence generation (selection bias)	Low risk	Participants were assigned a unique enrolment number using Interactive Web Response System (IWRS) or Interactive Voice Response System (IVRS) at visit 1 (Enrollment Visit). Participants were randomized to the treatment groups using the method of randomly permuted blocks with a block size of 4 after evaluation for all inclusion/exclusion criteria. Participants were stratified into one of the eight age-by-insulin use-by-time from most recent qualifying CV event strata<br>according to age group (<65 years vs. ≥65 years at enrolment), use of insulin (No versus Yes at randomization), and time from most recent qualifying CV

		event (more than 1 year vs. 1 year<br>or less [i.e., within 12 months] before enrolment).<br>Participants were given medication with the bottle number allocated by the IWRS/IVRS.
Allocation concealment (selection bias)	Low risk	Centralized randomization system used. Participants were given medication with the bottle number allocated by the IWRS/IVRS.
Blinding of participants and personnel (performance bias)	Low risk	Double blind placebo.
Blinding of outcome assessment (detection bias)	Low risk	Double blind placebo.
Incomplete outcome data (attrition bias)	Low risk	807/922 completed. All drop outs and losses to follow up accounted for.<br>The last observation carried forward approach was used for all variables at 24 weeks. ITT analysis employed.
Selective reporting (reporting bias)	Low risk	All clinically relevant outcomes were defined and reported.
Other bias	Unclear risk	This study was sponsored by Bristol-Myers Squibb and AstraZeneca. Medical writing assistance was funded by AstraZeneca. Some of the investigators are employees of Astra Zeneca.

DeFronzo 2015

**Methods**	52 week, phase 3, randomized, double blind, parallel-group study in 197 centers in 22 countries.
**Participants**	686 initially randomized, empagliflozin 25 mg/linagliptin 5mg (n = 137); empagliflozin 10 mg/linagliptin 5mg (n = 136); empagliflozin 25 mg (n = 141); empagliflozin 10 mg (n = 140); or linagliptin 5 mg (n = 132) as add-on to metformin.
**Interventions**	Randomized (1:1:1:1:1) to receive empagliflozin 25 mg/linagliptin 5 mg, empagliflozin 10 mg/linagliptin 5 mg FDC tablet, empagliflozin

	25 mg, empagliflozin 10 mg, or linagliptin 5 mg for 52 weeks.<br><br>Add on: metformin.<br><br>Rescue initiated if a subject had blood glucose >240 mg/dL.
**Outcomes**	**Primary outcomes of the trial**<br><br>change from baseline in HbA1c at week 24.<br><br>**Secondary outcomes of the trial**<br><br>change from baseline in FPG at week 24,<br>change from baseline in body weight at week 24,<br>proportion of subjects with baseline,<br>HbA1c ≥7% (≥53 mmol/mol) who had HbA1c <7% (<53 mmol/mol) at week 24.<br>change from baseline in HbA1c at week 24 in subgroups of subjects with HbA1c ≥8.5 and <8.5% at baseline,<br>change from baseline in HbA1c,<br>FPG,<br>weight,<br>systolic blood pressure (SBP), and diastolic blood pressure (DBP) at week 52,<br>proportion of subjects with baseline HbA1c ≥7% (≥53 mmol/mol) who had HbA1c <7% (<53 mmol/mol) at week 52.<br><br>**Adverse events and safety assessments**<br><br>vital signs,<br>clinical laboratory parameters,<br>all events with an onset after the first dose and up to 7 days after the last dose of study medication.<br>Confirmed hypoglycemic AEs<br>urinary tract infection (UTI),<br>genital infection,<br>volume depletion,<br>hypersensitivity reactions,<br>pancreatitis.
**Inclusion criteria**	men and women<br>aged >18 years<br>BMI >45 kg/m^2<br>HbA1c >7 to ≤10.5% (>53 to ≤91 mmol/mol) at screening<br>with metformin immediate release (≥1,500 mg/day, maximum tolerated dose, or maximum dose according to local label) at an unchanged dose for ≥12 weeks prior to randomization

	on a diet and exercise regimen.
**Exclusion criteria**	uncontrolled hyperglycemia (glucose level >240 mg/dL after an overnight fast,<br>treatment with any antidiabetes drug except metformin within 12 weeks prior to randomization<br>eGFR <60 mL/min/1.73 m2 using the Modification of Diet in Renal Disease (MDRD) equation,<br>acute coronary syndrome,<br>stroke, or transient ischemic attack within 3 months prior to consent,<br>bariatric surgery in the last 2 years,<br>investigational drug intake within 1 month prior to consent,<br>treatment with anti-obesity drugs within 3 months prior to consent.
**Notes**	ClinicalTrials.gov identifier: NCT01422876<br><br>DeFronzo 2015 participants randomized to receive empagliflozin for 24 weeks, and this was extended to 52 weeks within the trial, however the primary endpoint of change in HbA1c was measured at 24 weeks. The 52-week data are included in our analyses.

Risk of bias table

**Bias**	**Authors' judgement**	**Support for judgement**
Random sequence generation (selection bias)	Low risk	Interactive voice and web response system. "Randomization was performed using a third-party interactive voice and web response system" (stratified by HbA1c at screening and region (Europe, Asia, North America, and South America).
Allocation concealment (selection bias)	Unclear risk	Not described in detail but implied in computer generated random system.
Blinding of participants and personnel (performance bias)	Low risk	Double blind.
Blinding of outcome assessment	Low risk	Double blind.

(detection bias)		
Incomplete outcome data (attrition bias)	Low risk	674/686 completed (full analysis set; FAS). A last observation carried forward (LOCF) approach was used to impute missing continuous efficacy data.
Selective reporting (reporting bias)	Low risk	All clinically relevant outcomes are defined and reported.
Other bias	Unclear risk	Medical writing assistance, supported financially by Boehringer Ingelheim. This study was funded by Boehringer Ingelheim and Eli Lilly and Company.

Ferrannini 2010

**Methods**	24-week randomized parallel-group, double-blind, placebo-controlled phase 3 trial at 85 sites in the U.S., Canada, Mexico, and Russia.
**Participants**	485 initially randomized, 274 joined main cohort: 2.5 mg n=65; 5 mg n=64; or 10 mg n=70; dapagliflozin or placebo n=75.
**Interventions**	Randomly assigned equally once-daily placebo or 2.5, 5, or 10 mg dapagliflozin, administered once daily either in the morning for 24 weeks.<br>Rescue medication permitted.<br>Add on: diet/exercise counselling given.
**Outcomes**	**Primary outcomes of the trial**<br>change from baseline in A1C at week 24<br>**Secondary outcomes of the trial**<br>change from baseline at week 24 in FPG<br>body weight.<br>**Adverse events and safety assessments**<br>vital signs, laboratory measurements,<br>and adverse events<br>urinary tract infections (UTIs)<br>genital infections<br>self-monitored blood glucose daily for unusually high or low blood

	glucose event or any symptoms suggestive of hypoglycemia.
**Inclusion criteria**	men and women<br>T2DM<br>aged 18–77 years<br>BMI ≤45 kg/m^2<br>fasting Cpeptide ≥1.0 ng/ml<br>A1C 7.0–10%<br>treatment naive
**Exclusion criteria**	history of type 1<br>diabetes, serum creatinine ≥133 µmol/l (men) or ≥124 µmol/l (women),<br>urine albumin-to-creatinine ratio >200 mg/mmol,<br>aspartate transaminase and/or alanine transaminase >3 times the upper limits of normal,<br>creatine kinase ≥3 times the upper limit of normal,<br>symptoms of severely uncontrolled diabetes (including marked polyuria and polydipsia with >10% weight loss during the last 3 months before enrolment)<br>significant disease: renal hepatic, hematological, oncological, endocrine, psychiatric, rheumatic diseases,<br>a cardiovascular event (including New York Heart Association class III/IV congestive heart failure) within 6 months of enrolment,<br>severe uncontrolled blood pressure (systolic blood pressure ≥180 mmHg and/or diastolic blood pressure ≥110 mmHg).
**Notes**	Dapagliflozin 10 mg given in the morning vs placebo. Participants with high A1c put on open label rescue medicine - not included in our analyses. Results from main cohort only used in our analyses.<br><br>ClinicalTrials.gov identifier: NCT00528372

Risk of bias table

Bias	Authors' judgement	Support for judgement
Random sequence generation (selection bias)	Unclear risk	Randomly assigned.
Allocation concealment (selection bias)	Unclear risk	Not mentioned.
Blinding of participants	Low risk	Double blind placebo.

and personnel (performance bias)		
Blinding of outcome assessment (detection bias)	Low risk	Double blind placebo.
Incomplete outcome data (attrition bias)	Low risk	232/274 completed. All drop outs and losses to follow up accounted for.
Selective reporting (reporting bias)	Low risk	All clinical relevant data are defined and reported.
Other bias	Unclear risk	This study was funded by Bristol-Myers Squibb and AstraZeneca. A Bristol-Myers Squibb, employee provided writing and editorial support.

Ferrannini 2013

**Methods**	12-week Phase IIb, randomized, double-blind, placebo-controlled trial at 75 centers in 13 countries.
**Participants**	408 initially randomized: placebo n=82; 5mg empagliflozin n=81; 10 mg empagliflozin n=81; 25 mg empagliflozin n=82; open-label metformin n=80.
**Interventions**	Empagliflozin (5mg empagliflozin; 10 mg empagliflozin; 25 mg empagliflozin; versus placebo oral dose once daily, double-blind.<br><br>Add on: none, other than open label metformin arm 1000mg daily dose of metformin (500mg twice daily with meals) for the first 4 weeks. If fasted plasma glucose values remained >6.1 mmol/l, the metformin dose was increased to 1000 mg twice daily or up to the maximum tolerated dose.
**Outcomes**	**Primary outcomes of the trial**<br><br>change in HbA1c from baseline to week 12.<br><br>**Secondary outcomes of the trial**<br><br>change in FPG from baseline to week 12,<br>change in HbA1c from baseline over time,<br>proportion of patients who achieved an HbA1c ≤7.0% after 12 weeks of treatment,<br>proportion of patients who achieved an HbA1c lowering of ≥0.5%

	after 12 weeks of treatment, change in body weight from baseline to week 12 and PK of empagliflozin.<br><br>**Adverse events and safety assessments**<br><br>incidence and intensity of AEs,<br>withdrawal because of AEs,<br>clinically relevant new or worsening findings in physical examination and vital signs (blood pressure and pulse rate) reported as AEs,<br>clinically relevant new or worsening findings in 12-lead ECGs reported as AEs and clinical laboratory assessments.
**Inclusion criteria**	men and women<br>aged ≥18 and ≤79 years<br>T2DM<br>treatment naive (no antidiabetic medication for ≥10 weeks prior to screening) or on one antidiabetic drug (except thiazolidinediones, glucagonlike peptide-1 (GLP-1) analogues or insulin) at a stable dose for ≥10 weeks prior to screening.<br>HbA1c ≥6.5 to ≤9.0% for patients treated with one other antidiabetic drug, or HbA1c >7.0 to ≤10.0% for treatment-naive patients. At start of placebo run-in, HbA1c was required to be >7.0 to ≤10.0%.<br>BMI ≤40 kg/m^2
**Exclusion criteria**	myocardial infarction, stroke or transient ischemic attack ≤6months prior to informed consent,<br>impaired hepatic function, renal insufficiency or impaired renal function defined as calculated creatinine clearance <0.84 ml/s/m2 or serum creatinine levels ≥132.6 μ mol/l for men and ≥123.8 mol/l for women,<br>unstable or acute congestive heart failure,<br>acute or chronic acidosis,<br>disease of central nervous system, psychiatric disorders or clinically relevant neurologic disorders,<br>chronic or clinically relevant acute infections,<br>current or chronic urogenital tract infection,<br>dehydration,<br>history of clinically relevant allergy/hypersensitivity, intolerance to metformin,<br>hereditary galactose intolerance,<br>treatment with thiazolidinediones, GLP-1 analogues or insulin ≤3months prior to informed consent,<br>treatment with anti-obesity drugs,<br>current treatment with systemic steroids,

	alcohol abuse,<br>treatment with an investigational drug ≤2 months prior to informed consent,<br>pregnancy, breastfeeding, not practising an acceptable method of birth control (female patients only).
**Notes**	Empagliflozin vs placebo, background treatment one other OAD (-glitazones, GLP-1analoges or insulin). Open label metformin arm not included.<br><br>A 78-week extension of the study, EMPA Ferrannini 2013b, is an open trial, placebo switched to active, data not included in these analyses.<br><br>ClinicalTrials.gov Identifier: NCT00789035

Risk of bias table

**Bias**	**Authors' judgement**	**Support for judgement**
Random sequence generation (selection bias)	Low risk	Interactive voice and web response system, stratified by country and by number of previous antidiabetic medications.
Allocation concealment (selection bias)	Unclear risk	Not described in detail but implied in computer generated random system.
Blinding of participants and personnel (performance bias)	Low risk	Double blind.
Blinding of outcome assessment (detection bias)	Low risk	Double blind.
Incomplete outcome data (attrition bias)	Unclear risk	406/408 who were treated were included in the analysis. Two participants were randomized but not treated; the treated set consisted of all randomized patients who received at least one dose of the study drug. No participant flow chart was provided. Reasons for dropouts and losses to follow up were not provided.<br><br>Comment: insufficient information, therefore judged as

		unclear.
Selective reporting (reporting bias)	Low risk	All clinically relevant outcomes are defined and reported.
Other bias	Unclear risk	Medical writing assistance, supported financially by Boehringer Ingelheim. Some of the trialists were employees of Boehringer Ingelheim.

Forst 2014

**Methods**	26 week randomized, double-blind, placebo-controlled phase 3 trial conducted at 74 centers in 11 countries.
**Participants**	344 initially randomized, canagliflozin 100mg n=113; canagliflozin 300mg n= 114; placebo n=115.
**Interventions**	Canagliflozin 100 or 300 mg versus placebo (oral dose) for 26 weeks.<br>Add on: standard counselling on diet and exercise, metformin, pioglitazone
**Outcomes**	**Primary outcomes of the trial**<br>efficacy end-point change from baseline in HbA1c at week 26.<br>**Secondary outcomes of the trial**<br>proportion of patients reaching HbA1c <7.0%,<br>change from baseline in FPG,<br>systolic BP<br>fasting index of β-cell function, HomeostasisModel Assessment (HOMA2-%B),<br>percent change from baseline in body weight, high-density lipoprotein cholesterol (HDL-C) and triglycerides.<br>**Adverse events and safety assessments**<br>Adverse event (AE) reports,<br>safety laboratory tests,<br>vital sign measurements,<br>physical examinations,<br>SMBG and 12-lead electrocardiograms.<br>urinary tract infections (UTIs),<br>genital mycotic infections,<br>specific analyses were performed for AEs related to osmotic

	diuresis and volume depletion,<br>hypoglycemia episodes.
**Inclusion criteria**	men and women<br>with T2DM<br>aged ≥18 and ≤80 years,<br>inadequate glycemic control (HbA1c ≥7.0% (53 mmol/mol) to ≤10.5% (91 mmol/mol)) on stable metformin therapy (2,000 mg/day (or 1,500 mg/day if unable to tolerate higher dose)) for 8 weeks<br>fasting plasma glucose (FPG) <15 mmol/l at week 2<br>fasting fingerstick glucose ≥6.1 mmol/l and <15 mmol/l on day 1.
**Exclusion criteria**	FPG and/or fasting self-monitored blood glucose (SMBG) ≥15.0 mmol/l (270 mg/dl) during the pretreatment phase;<br>history of type 1 diabetes, cardiovascular disease (including myocardial infarction,<br>unstable angina, revascularization procedure or cerebrovascular accident) within 3 months prior to screening,<br>uncontrolled hypertension;<br>ongoing eating disorder or 5% change in body weight within 12 weeks;<br>eGFR <55 ml/min/1.73m^2 (or <60 ml/min/1.73m2 if based upon restriction of metformin use in local label) or<br>serum creatinine ≥124 µmol/l for men and ≥115 µmol/l for women.
**Notes**	Only core-period included in this meta-analysis as placebo group was changed to sitagliptin.<br><br>ClinicalTrials.gov identifier: NCT01106690<br><br>Extension study of 26 weeks, placebo changed to sitagliptin for another 26 weeks. We only use data from the first 26-week double-blinded period. Patients and study center and local sponsor personnel remained blinded throughout the extension period.

Risk of bias table

**Bias**	**Authors' judgement**	**Support for judgement**
Random sequence generation (selection bias)	Low risk	Interactive voice response system or interactive web response system. Randomization was balanced using permuted blocks of six patients per block and stratified according to:(i) whether a patient entered the AHA

		adjustment period and (ii) dose of pioglitazone at randomization.
Allocation concealment (selection bias)	Unclear risk	Not described but implied in details of randomization which used an interactive voice response system or interactive web response system.
Blinding of participants and personnel (performance bias)	Low risk	Double blind "patients received single-blind placebo capsules matching study drug once daily"
Blinding of outcome assessment (detection bias)	Low risk	Double blind "After completion of the core treatment period, the database was locked and the study was unblinded by the sponsor for regulatory filing. Patients and study center and local sponsor personnel remained blinded throughout the extension period."
Incomplete outcome data (attrition bias)	Low risk	296/344 completed the 26 week 'core' period.<br>Efficacy endpoints at week 26 were assessed using the modified intention-to-treat (mITT) population. Drop outs and losses to follow up accounted for.
Selective reporting (reporting bias)	Unclear risk	No safety data for week 26.
Other bias	Unclear risk	This study was sponsored by Janssen Research & Development, LLC. Editorial support was provided by Janssen Global Services LLC.

Gonzalez 2013

**Methods**	26 week randomized, double-blind, four-arm, parallel group, Phase 3 study was conducted at 169 centers in 22 countries.
**Participants**	Initially randomized n= 1284: canagliflozin 100mg n=368; canagliflozin 300mg n= 367; sitagliflozin n=366; placebo n= 183.
**Interventions**	Canagliflozin 100 mg or 300 mg, sitagliptin 100 mg or placebo (2:2:2:1) once daily for 26 weeks (oral dose)<br>Add on: metformin monotherapy.<br>Glycemic rescue therapy with glimepiride permitted.

**Outcomes**	**Primary outcomes of the trial**<br><br>reduction of HbA1c from baseline to week 26.<br><br>**Secondary outcomes of the trial**<br><br>proportion of participants reaching HbA1c <7.0% (53 mmol/mol),<br>change in FPG,<br>2h postprandial glucose (PPG)<br>systolic BP<br>percent change in body weight,<br>triacylglycerol (i.e. triglycerides) and HDL-cholesterol,<br>all participants underwent a mixed-meal tolerance test (MMTT)<br><br>**Adverse effects**<br><br>AE reports,<br>safety laboratory tests,<br>vital sign measurements,<br>physical examinations,<br>SMBG and 12-lead electrocardiograms.<br>urinary tract infections (UTIs)<br>genital mycotic infections.<br>documented episodes of hypoglycemia.
**Inclusion criteria**	men and women<br>with T2DM<br>aged ≥18 and ≤ 80 years,<br>inadequate glycemic control (HbA1c ≥7.0% (53 mmol/mol) and ≤10.5% (91 mmol/mol))<br>on stable metformin therapy (≥2,000 mg/day (or ≥1,500 mg/day if unable to tolerate higher dose)) for 8 weeks<br>fasting plasma glucose (FPG) <15 mmol/l at week 2<br>fasting fingerstick glucose ≥6.1 mmol/l and <15 mmol/l on day 1.
**Exclusion criteria**	repeated FPG and/or fasting self-monitored blood glucose (SMBG) ≥15.0 mmol/l during the pretreatment phase,<br>history of type 1 diabetes,<br>cardiovascular disease (including myocardial infarction, unstable angina, revascularization procedure or cerebrovascular accident) in the 3 months before screening or uncontrolled hypertension,<br>treatment with a peroxisome proliferator-activated receptor γ agonist, insulin, another SGLT2 inhibitor or any other AHA (except metformin as monotherapy or in combination with a sulfonylurea) in the 12 weeks before screening,<br>eGFR <55 ml min^{-1} (1.73 $m^2)^{-1}$ (or <60 ml min−1 (1.73 $m2)^{-1}$ if

	based upon restriction in local label) or serum creatinine ≥124 µmol/l (men) or ≥115 µmol/l (women).
**Notes**	ClinicalTrials.gov identifier: NCT01106677<br>Study continued for 26 weeks after initial 26-week period, placebo group switched to sitagliflozin (second period not included in our analyses).

Risk of bias table

**Bias**	**Authors' judgement**	**Support for judgement**
Random sequence generation (selection bias)	Low risk	The computer-generated randomization schedule was prepared by the sponsor before the study.
Allocation concealment (selection bias)	Unclear risk	The computer-generated randomization schedule was prepared by the sponsor before the study.
Blinding of participants and personnel (performance bias)	Low risk	After randomization, HbA1c and FPG values were masked to the study centers unless they met glycemic rescue criteria.
Blinding of outcome assessment (detection bias)	Low risk	After completion of period I (first 26 weeks), the database was locked and the study was unblinded by the sponsor for regulatory filing;
Incomplete outcome data (attrition bias)	Low risk	1119/1284 completed the first 26-week period.<br>All drop outs and losses to follow up accounted for.<br>Primary efficacy analysis was performed in the modified intent-to-treat (mITT) population (randomized participants who received 1 dose of study drug) using a last observation carried forward (LOCF) approach.<br>Primary efficacy analyses were performed in the mITT population according to randomized treatment assignment using LOCF to impute missing data; for participants who received rescue therapy, the last post-baseline value before rescue was used.

Selective reporting (reporting bias)	Low risk	All major clinically relevant outcomes were reported
Other bias	Unclear risk	This study was supported by Janssen Research & Development, LLC who contributed to clinical management, data review and preparation of the study report.

Haring 2014

**Methods**	76-week, randomized, double-blind, placebo-controlled, parallel-group study in 148 centers across 12 countries
**Participants**	638 initially randomized: (empagliflozin 10 mg, n = 217; empagliflozin 25 mg, n = 214; placebo, n = 207)
**Interventions**	Randomized (1:1:1) to receive once-daily empagliflozin 10 mg, empagliflozin 25 mg, or placebo.<br>Add on: metformin<br>Rescue mediation permitted in case of hyperglycemia.
**Outcomes**	**Primary outcomes of the trial**<br>change from baseline in HbA1c at week 24.<br>**Secondary outcomes of the trial**<br>change from baseline to week 24 in body weight,<br>weighted mean daily glucose (MDG) level using an 8-point blood glucose profile,<br>percentage of patients with baseline HbA1c level ≥7.0% (≥53 mmol/mol) who had an HbA1c level <7% (<53 mmol/mol) at week 24,<br>changes from baseline to week 24 in fasting plasma glucose (FPG),<br>waist circumference,<br>systolic BP (SBP),<br>diastolic BP (DBP),<br>percentage of patients with >5% reduction in body weight at week 24;<br>percentage of patients with uncontrolled BP at baseline who had controlled BP (SBP <130 and DBP <80 mmHg) at week 24<br>use of rescue medication,<br>change from baseline in 2-h postprandial glucose (PPG) was assessed in a subset of patients based on a meal tolerance test

	(MTT) at baseline and week 24.<br><br>**Adverse events and safety assessments**<br><br>vital signs,<br>clinical laboratory parameters,<br>12-lead electrocardiogram findings,<br>adverse events (all),<br>all events with an onset after the first dose of trial medication up to a period of 7 days after the last dose,<br>confirmed hypoglycemic AEs (plasma glucose ≤3.9 mmol/L and/or requiring assistance),<br>suspected urinary tract infection (UTI),<br>genital infection.
**Inclusion criteria**	adults aged ≥18 years,<br>T2DM,<br>BMI ≤45kg/m2) with inadequately controlled type 2 diabetes (HbA1c ≥7% to ≤10% (≥53 to ≤86 mmol/mol)) despite undergoing a diet and exercise program and a stable immediate release metformin regimen (unchanged for ≥12 weeks prior to randomization).
**Exclusion criteria**	uncontrolled hyperglycemia (glucose level >13.3 mmol/L) after an overnight fast confirmed by a second measurement, acute coronary syndrome,<br>stroke, or transient ischemic attack within 3 months prior to informed consent,<br>indication of liver disease (alanine aminotransferase, alkaline aminotransferase, or alkaline phosphatase levels more than three times the upper limit of normal)<br>impaired kidney function (estimated eGFR <30 mL/min/1.73 m2) during screening or run-in<br>contraindications to metformin according to the local label<br>bariatric surgery or other gastrointestinal surgeries that induce chronic malabsorption<br>medical history of cancer (except for basal cell carcinoma) or treatment for cancer within the last 5 years<br>blood dyscrasias or any disorders causing hemolysis or unstable erythrocytes,<br>treatment with anti-obesity drugs 3 months prior to consent,<br>use of any treatment at screening leading to unstable body weight<br>treatment with systemic steroids at the time of consent<br>change in the dosage of thyroid hormones within 6 weeks prior to consent,<br>alcohol or drug abuse within 3 months of consent

	investigational drug intake in another trial within 30 days prior to the current trial.
**Notes**	ClinicalTrials.gov identifier: NCT01159600<br><br>Haring 2014 includes data from Merker 2015, a double-blind extension trial for ≥ 52 weeks i.e. 76 weeks in total.

Risk of bias table

Bias	Authors' judgement	Support for judgement
Random sequence generation (selection bias)	Low risk	Randomization was performed using a third-party interactive voice and web response system, and was stratified by HbA1c level and eGFR.
Allocation concealment (selection bias)	Unclear risk	Not described, but implied in the computer-generated stratified randomization sequence technique.
Blinding of participants and personnel (performance bias)	Low risk	Double-blind.
Blinding of outcome assessment (detection bias)	Low risk	Double-blind.
Incomplete outcome data (attrition bias)	Low risk	591/638 completed. One patient assigned to receive empagliflozin 25 mg was not treated The FAS comprised 637 patients, who were treated with one or more doses of study drug and who had a baseline HbA1c value.
Selective reporting (reporting bias)	Low risk	All clinically relevant outcomes are defined and reported.
Other bias	Unclear risk	This research was supported by Boehringer Ingelheim and Eli Lilly. Medical writing assistance was supported by Boehringer Ingelheim. Some of the trialists are employees of Boehringer Ingelheim.

Henry 2012

**Methods**	24-week, randomized, double-blind three-arm active-controlled studies conducted at 131 sites in 4 locations, North America, Latin America, Europe and Asia.
**Participants**	641 initially randomized: dapagliflozin 10 mg plus metformin n=211; dapagliflozin 10 mg plus placebo n=219; dapagliflozin 10 mg metformin plus placebo n=208
**Interventions**	Randomly assigned (1:1:1). Dapagliflozin 10 mg plus metformin XR (combination), dapagliflozin 10 mg plus placebo (dapagliflozin), and metformin XR plus placebo (metformin).<br>Drug administration occurred with the evening meal.<br>Add on: diet and exercise counselling.<br>Rescue medication permitted.
**Outcomes**	**Primary outcomes of the trial**<br>HbA1c change from baseline at week 24.<br>**Secondary outcomes of the trial**<br>change from baseline at week 24 in fasting plasma glucose,<br>proportion of patients achieving a therapeutic glycemic response (HbA1c <7%),<br>HbA1c for patients with baseline HbA1c ≥9%,<br>total body weight,<br>proportion discontinued or rescued for failing to achieve prespecified glycemic targets based on prespecified rescue criteria,<br>non-inferiority of dapagliflozin<br>10 mg to metformin XR for changes in fasting<br>plasma glucose (0.83 mmol ⁄ l margin) and HbA1c (0.35% margin);<br>if non-inferiority was demonstrated<br>superiority of dapagliflozin 10 mg was tested,<br>difference in weight reduction with dapagliflozin 10 mg vs. metformin<br>**Adverse events and safety assessments**<br>vital signs,<br>laboratory measurements<br>adverse events<br>vulvovaginitis, balanitis and related genital infection.

**Inclusion criteria**	men and women<br>T2DM uncontrolled by diet and exercise<br>18–77 years,<br>hemoglobin A1c (HbA1c) 7.5–12%,<br>body mass index ≤45 kg ⁄ m^2,<br>C-peptide concentration ≥ 0.33 nmol ⁄ l
**Exclusion criteria**	serum creatinine ≥132.60 μmol ⁄ l (men) or ≥123.76 μmol ⁄ l (women) consistent with metformin labelling,<br>urine albumin:creatinine ratio > 1800 mg⁄ g,<br>serum aspartate transaminase or alanine,<br>transaminase >3 times upper limit of normal (ULN),<br>creatine kinase >3 times ULN,<br>history of diabetes insipidus,<br>symptoms of poorly controlled diabetes (including marked polyuria and polydipsia with > 10% weight loss during 3 months before enrolment),<br>clinically significant renal, hepatic, hematological, oncological, endocrine, psychiatric or rheumatic disease,<br>a cardiovascular event within 6 months or New York Heart Association Class III or IV congestive heart failure<br>systolic blood pressure ≥180 or diastolic blood pressure ≥110 mmHg.
**Notes**	Three arms, metformin + placebo vs. dapagliflozin + placebo included. Dapa+ metformin not included in our analyses.<br><br>ClinicalTrials.gov identifier: Study 2 (NCT00859898), only data from study 2 (10mg dapagliflozin) are included in our analyses.

Risk of bias table

**Bias**	**Authors' judgement**	**Support for judgement**
Random sequence generation (selection bias)	Low risk	Random numbers (stratified by site, in blocks of three, and randomly assigned (1:1:1) to double-blinded groups of combination therapy, dapagliflozin monotherapy and metformin monotherapy by IVRS.)
Allocation concealment (selection bias)	Unclear risk	Not stated, but implied by randomization using a central interactive voice response system.
Blinding of participants and	Low risk	Double-blind.

personnel (performance bias)		
Blinding of outcome assessment (detection bias)	Low risk	Double-blind.
Incomplete outcome data (attrition bias)	Low risk	552/641completed. Primary efficacy data were derived from randomized patients with ≥1 dose of double-blind medication, and with baseline and ≥1 post baseline measurements. Last observation carried forward. All drop outs and losses to follow up accounted for.
Selective reporting (reporting bias)	Low risk	All clinically relevant outcomes are defined and reported.
Other bias	Unclear risk	These studies were funded by Bristol-Myers Squibb and AstraZeneca. Employees of Bristol-Myers Squibb provided writing and editorial assistance.

Häring 2013

**Methods**	76-week, randomized, double-blind, placebo-controlled trial in 148 centers in 12 countries
**Participants**	669 initially randomized: empagliflozin 10 mg n=226, empagliflozin 25 mg n=218; or placebo n=225.
**Interventions**	Randomized (1:1:1) to receive once-daily oral dose empagliflozin 10 mg, empagliflozin 25 mg, versus placebo therapy to metformin (≥1,500 mg/day or maximum tolerated dose or maximum dose according to local label)<br>Add on: metformin, sulfonylurea (greater than or equal to half the maximum recommended dose, or the maximum tolerated dose, or the maximum dose according to local label).
**Outcomes**	**Primary outcomes of the trial**<br>change from baseline in HbA1c at week 24.<br>**Secondary outcomes of the trial**<br>change from baseline to week 24 in body weight and<br>mean daily glucose (MDG) using an 8-point blood glucose profile.<br>percentage with baseline HbA1c ≥7.0% who had HbA1c <7% at

	week 24; change from baseline in fasting plasma glucose (FPG),<br>waist circumference,<br>systolic and diastolic blood pressure (SBP and DBP)<br>percentage of patients with .5% reduction in body weight at week 24<br>use of rescue medication.<br>change from baseline in 2h postprandial glucose (PPG) was assessed in a subset of patients based on a meal tolerance test (MTT) performed<br><br>**Adverse events and safety assessments**<br><br>vital signs,<br>clinical laboratory parameters,<br>12-lead electrocardiogram,<br>confirmed hypoglycemic AEs (plasma glucose ≤3.9 mmol/L and/or requiring assistance)<br>urinary tract infection (UTI) and genital infection.
**Inclusion criteria**	aged ≥18 years<br>BMI ≤45 kg/m^2<br>inadequately controlled type 2 diabetes (HbA1c ≥7 to ≤10%)<br>despite a diet and exercise program<br>on stable metformin immediate release plus a sulfonylurea for at least 12 weeks prior
**Exclusion criteria**	**Exclusion criteria**<br><br>uncontrolled hyperglycemia (glucose level >13.3 mmol/L) after an overnight fast, confirmed by a second measurement),<br>acute coronary syndrome, stroke or transient ischemic attack within 3 months prior to consent,<br>indication of liver disease,<br>impaired kidney function (estimated glomerular filtration rate (eGFR) <30 mL/min/1.73 m^2)<br>contraindications to metformin or sulfonylurea,<br>gastrointestinal surgeries that induce chronic malabsorption,<br>history of cancer (except basal cell carcinoma) or treatment for cancer within 5 years,<br>blood dyscrasias or any disorders causing hemolysis or unstable erythrocytes,<br>treatment with anti-obesity drugs 3 months prior to consent,<br>use of any treatment at screening that leads to unstable body weight,<br>treatment with systemic steroids at time of consent,<br>change in dosage of thyroid hormones within 6 weeks of consent,

	alcohol or drug abuse<br>investigational drug intake within 30 days of the trial.
**Notes**	The open label arm in Häring 2013 including patients with HbAb1c >10% is not included in the analyses in this review.<br>ClinicalTrials.gov identifier: NCT01159600. The 52-week extension data (i.e. 76 weeks in total) from Häring 2015 are included in the analyses.

Risk of bias table

**Bias**	**Authors' judgement**	**Support for judgement**
Random sequence generation (selection bias)	Low risk	Third-party interactive voice and web response system and was stratified by HbA1c.
Allocation concealment (selection bias)	Unclear risk	Not described in detail but implied in interactive voice and web response system (computer generated) used for randomization.
Blinding of participants and personnel (performance bias)	Low risk	Double blind placebo controlled.
Blinding of outcome assessment (detection bias)	Low risk	Double blind placebo controlled.
Incomplete outcome data (attrition bias)	Low risk	666/669 initially randomized completed (contributed data to the final analysis set FAS). Last observation carried forward. All drops outs and losses to follow up accounted for.
Selective reporting (reporting bias)	Low risk	All clinical relevant outcomes are defined and reported.
Other bias	Unclear risk	This study was funded by Boehringer Ingelheim. Medical writing assistance, was supported financially by Boehringer Ingelheim.

Inagaki 2013

**Methods**	12 week, multicenter, randomized, placebo-controlled, parallel-group study
**Participants**	383 initially randomized. 300mg canagliflozin n=75; 200mg canagliflozin n=77; 100mg canagliflozin n=74; placebo n=75
**Interventions**	Canagliflozin (50, 100, 200 or 300 mg once daily (oral dose) versus placebo for 12 weeks.<br>Eligible patients were randomized using a block allocation method into one of five groups (ratio 1:1:1:1:1) to receive placebo or one of the four doses of canagliflozin.<br>Add on: diet and exercise therapy.
**Outcomes**	**Primary outcomes of the trial**<br>change in HbA1c from the last day of the run-in period (baseline) to the end of the treatment period (12 weeks).<br>**Secondary outcomes of the trial**<br>change in FPG,<br>percentages of patients with HbA1c <7.0%,<br>changes in urinary glucose/creatinine ratio,<br>body weight,<br>BMI,<br>waist circumference,<br>lipid levels,<br>blood pressure,<br>insulin and proinsulin levels,<br>homeostasis model assessment of β-cell function (HOMA-β)<br>meal tolerance-related parameters.<br>**Adverse events and safety assessments**<br>AEs and safety assessments (AEs were classified according to system organ class in terms of their potential relationship with the study drug (no causal relationship or possible causal relationship) and severity (mild, moderate or severe),<br>vital signs,<br>12-lead electrocardiography,<br>clinical laboratory tests (blood chemistry, hematology, coagulation, bone markers and urinalysis),<br>hypoglycemic symptoms.
**Inclusion criteria**	men and women<br>T2DM for at least 3 months before the run-in period,

	HbA1c levels of 6.9–9.9% at the start of the run-in period,<br>to have undergone diet and exercise therapy, with no change in their regimen for !8 weeks before the study.<br>aged 20–80 years<br>Japanese
**Exclusion criteria**	history of or current serious diabetic complications (e.g. proliferative diabetic retinopathy, stage 3 or later overt nephropathy, diabetic ketoacidosis or serious diabetic neuropathy),<br>FPG ≥270 mg/dl (1 mg/dl FPG=0.0555 mmol/l),<br>indication for insulin therapy,<br>hereditary glucose-galactose malabsorption<br>renal glycosuria.
**Notes**	ClinicalTrials.gov identifier: NCT01022112<br>"In terms of concomitant treatments, antihyperglycemic drugs were prohibited after randomization until the end of the follow-up period. Diet and exercise interventions were to be continued without modification after randomization until the end of the follow-up period."<br>"Patient characteristics were generally well balanced among the five groups, with no marked differences among treatment groups (Table 1) except for height, body weight and presence/absence of neuropathy or hypertension, which were unbalanced among the five groups at p<0.15."

Risk of bias table

**Bias**	**Authors' judgement**	**Support for judgement**
Random sequence generation (selection bias)	Low risk	Randomization was conducted by a central committee, which provided the investigators at each site with randomization codes stored in sealed envelopes.
Allocation concealment (selection bias)	Low risk	The randomization code was stored in sealed envelopes was not to be broken until data entry had been completed or unless needed in an emergency.
Blinding of participants and personnel	Low risk	Investigators and patients were blinded to the study drug received during the treatment phase.

(performance bias)		
Blinding of outcome assessment (detection bias)	Low risk	Investigators and patients were blinded to the study drug received during the treatment phase.
Incomplete outcome data (attrition bias)	Low risk	361/383 completed (i.e. 22 withdrew after randomization). All drops outs and losses to follow up accounted for.<br><br>Primary and secondary analyses were conducted in the full analysis set (FAS), defined as all allocated patients, excluding patients who did not receive any study drug or who did not have any efficacy data after entering the treatment phase. In the event of missing data for efficacy variables, the last observation carried forward (LOCF) approach was used to impute missing values in the FAS analyses.
Selective reporting (reporting bias)	Low risk	All major and clinically relevant outcomes were reported
Other bias	Unclear risk	This study was funded by Mitsubishi Tanabe Pharma Corporation, the manufacturer of canagliflozin.

Jabbour 2014

**Methods**	24-week, randomized, double-blind, placebo-controlled, parallel-group, phase 3 study, multicenter in 6 countries (Argentina, Germany, Mexico, Poland, the United Kingdom, and the United States)
**Participants**	451 initially randomized: dapagliflozin n = 225; placebo n = 226.
**Interventions**	Dapagliflozin 10 mg or placebo (oral dose, once daily)<br><br>Add on: metformin, sitagliptin. Participants in stratum 1, study treatment was added to sitagliptin monotherapy; in stratum 2 received open-label oral metformin immediate release 500 mg tablets (≥1500 mg/day).<br><br>A rescue therapy, open-label oral glimepiride permitted ≤6 mg/day, was given to patients with FPG >270 mg/dl (15.0 mmol/l)—weeks 0–4; FPG >240 mg/dl (13.3 mmol/l)—weeks 4–12; or FPG >200 mg/dl

	(11.1 mmol/l) or HbA1c >8.0% (64 mmol/mol)—weeks 12–24.<br><br>Sitagliptin monotherapy was allowed only in countries where it was approved. No other oral antidiabetic agents permitted.
**Outcomes**	**Primary outcomes of the trial**<br><br>change in HbA1c from baseline at week 24.<br><br>**Secondary outcomes of the trial**<br><br>change in total body weight from baseline to week 24,<br>change in HbA1c in patients with baseline HbA1c ≥8% (64 mmol/mol),<br>change in FPG from baseline to week 24, change in seated SBP in patients with baseline seated SBP ≥130 mmHg from baseline to week 8,<br>glycemic response rate (HbA1c reduction ≥0.7% (7.7 mmol/mol) from baseline),<br>change in 2-h post liquid meal glucose (PPG) from baseline (day 0) (PPG, C-peptide, and insulin determination),<br>proportion of subjects achieving a therapeutic glycemic response (HbA1c <7.0% (53 mmol/mol)),<br>change in seated SBP from baseline,<br>percent change in fasting lipids (total cholesterol, low-density lipoprotein cholesterol, high-density lipoprotein cholesterol, and triglycerides),<br>change in β-cell function (HOMA-2)),<br>insulin resistance (as measured by the HOMA for Insulin Resistance).<br><br>**Adverse events and safety assessments**<br><br>reported adverse events (AEs),<br>laboratory values,<br>electrocardiogram, pulse, BP,<br>hypoglycemic events,<br>calculated creatinine clearance,<br>estimated glomerular filtration rate,<br>physical examination findings.
**Inclusion criteria**	men and women<br>≥18 years (An upper age limit was imposed for those receiving metformin where local label restrictions applied). Mean age approximately 55 years.<br>T2DM<br>HbA1c values between 7.7% (61 mmol/mol) and 10.5% (91

	mmol/mol) for individuals not receiving a DPP-4 inhibitor at enrolment and between 7.2% (55 mmol/mol) and 10.0% (86 mmol/mol) for those receiving a DPP-4 inhibitor.<br>prior to randomization, HbA1c values were required to be between 7.0% (53 mmol/mol) and 10.0% (86 mmol/mol) for all patients.
**Exclusion criteria**	type 1 diabetes<br>fasting plasma glucose (FPG) >270 mg/dL (15.0 mmol/l)<br>pregnant or breast-feeding women<br>treatment with OADs other than metformin or DPP-4 inhibitors within the 10 weeks prior to enrolment<br>receiving metformin with a calculated creatinine clearance <60 ml/min or serum creatinine values ≥1.5 mg/dl for men or ≥1.4 mg/dl for women<br>if not treated with metformin and with a baseline calculated creatinine clearance <50 ml/min SBP ≥170 mmHg and/or diastolic BP (DBP) ≥110 mmHg (at randomization SBP <160 mmHg and/or a DBP <100 mmHg)
**Notes**	ClinicalTrials.gov identifier: NCT00984867<br>24-week blinded extension period (not included in our analyses as it was only site- and patient-blind).

Risk of bias table

**Bias**	**Authors' judgement**	**Support for judgement**
Random sequence generation (selection bias)	Low risk	Not described, but use of stratification implies computerized method.<br>Randomized patients were stratified by concomitant metformin use at baseline: in stratum 1, study treatment was added to sitagliptin monotherapy; in stratum 2, study treatment was added to sitagliptin plus metformin IR (≥1500 mg/day, administered BID with meals).
Allocation concealment (selection bias)	Unclear risk	Not described.
Blinding of participants and personnel	Low risk	24-week double-blind period, (we excluded the 24-week extension: only site- and patient-blind period).

(performance bias)		
Blinding of outcome assessment (detection bias)	Low risk	24-week double-blind period, (we excluded the 24-week extension: only site- and patient-blind period).
Incomplete outcome data (attrition bias)	Low risk	411/451 completed. Efficacy data were analyzed with a full analysis set (FAS) that included all randomized individuals who took at least one dose of double-blind study medication, had a non-missing baseline value and ≥1 post-baseline efficacy value for ≥1 efficacy variable. The safety set comprised patients who took ≥1 dose of double-blind study medication. All drop outs and losses to follow up accounted for. (Minor discrepancy between paper n=432 randomized and supplementary data n=451)
Selective reporting (reporting bias)	Low risk	All major clinically relevant outcomes were reported
Other bias	Unclear risk	This study was sponsored by Bristol-Myers Squibb and AstraZeneca. Medical writing assistance was funded by Bristol-Myers Squibb and AstraZeneca.

Ji 2014

**Methods**	24- week randomized, double-blind, placebo-controlled, parallel group, phase III study at 40 sites (26 in China, 5 each in Korea and Taiwan, and 4 in India)
**Participants**	393 initially randomized: placebo n=132; dapagliflozin 5 mg n=128; dapagliflozin 10 mg n=133
**Interventions**	Patients randomized to Dapagliflozin 5 mg or 10 mg or placebo once daily for 24 weeks taken orally once per day before the first meal of the day.<br>Six weeks lead-in on diet and exercise.<br>Rescue medication permitted (metformin (500mg daily, titrated to 2000 mg if necessary)<br>Add on: none
**Outcomes**	**Primary outcomes of the trial**

	mean change in HbA1c level at week 24 dapagliflozin vs placebo<br>using the last-observation carried forward (LOCF)<br><br>**Secondary outcomes of the trial**<br><br>change from baseline in FPG<br>change from baseline<br>in 2-hourPPG (after a liquid meal challenge)<br>change from baseline in total weight<br>proportion of patients achieving a therapeutic glycemic response,<br>defined as HbA1c levels <7.0% (53mmol/mol),<br>change from base- line in β cell function and insulin resistance<br>(HOMA-2),<br>waist circumference<br>lipids (total cholesterol, LDL-C,HDL-C, and fasting triglyceride)<br>≥ 3% or ≥ 5% reduction<br>spot fasting urinary glucose to creatinine ratio<br><br>**Adverse events and safety assessments**<br><br>discontinuations due to AEs,<br>laboratory tests<br>vital signs,<br>hypoglycemia,<br>genital infection and UTI,
**Inclusion criteria**	men and women >18 years,<br>type 2 diabetes,<br>drug naive, Including Chinese herbal medicines<br>HbA1c ≥7.5 ≤10.5 % at enrolment<br>C-peptide level ≥1.0 ng/mL(0.34nmol/L)<br>BMI < 45 kg/m2.
**Exclusion criteria**	diabetes insipidus<br>symptoms of poorly controlled diabetes including marked polyuria<br>and polydipsia with >10%weight loss<br>diabetic ketoacidosis<br>hyper osmolar nonketotic coma<br>bone fractures (osteoporosis)<br>severe uncontrolled hypertension<br>myocardial infarction<br>cardiac surgery bypass surgery<br>unstable angina<br>congestive heart failure<br>transient ischemic attack or significant cerebrovascular disease<br>arrhythmia

	renal disease<br>congenital renal glycosuria<br>Significant hepatic or hepatobiliary disease<br>history of hepatotoxicity<br>hemoglobinopathy,<br>donation of blood or blood products to a blood bank, blood transfusion,<br>malignancy<br>immunocompromised, HIV<br>allergies to study medications or metformin<br>any antidiabetic therapy, including Chinese traditional medicine,<br>systemic corticosteroid therapy,<br>bariatric surgery<br>administration of sibutramine, phentermine, orlistat, rimonabant, benzphetamine, diethylpropion, methamphetamine, and/orphendimetrazine within 30daysofenrollmentvisit<br>at risk for dehydration or volume depletion<br>alcohol or other drug<br>previous participation in a clinical trial with dapagliflozin and/oranyotherSGLT2-inhibitors<br>other investigational drug within 30 days<br>unstable or serious vascular, renal, hepatic, hematologic, oncologic, endocrine, psychiatric, or rheumatic diseases<br>severe hyper triglyceridemia
**Notes**	Dapagliflozin 10 mg only included in our analyses.<br>ClinicalTrials.gov identifier: NCT01095653

Risk of bias table

**Bias**	**Authors' judgement**	**Support for judgement**
Random sequence generation (selection bias)	Low risk	Randomized sequentially by using a random numbers in a 1:1:1 ratio, stratified according to site, Using interactive voice response system in a blinded manner to one of three treatment groups, stratified according to site.
Allocation concealment (selection bias)	Unclear risk	Not stated, but implied by interactive voice response system for randomization.
Blinding of participants and	Low risk	Double-blind.

personnel (performance bias)		
Blinding of outcome assessment (detection bias)	Low risk	Double-blind.
Incomplete outcome data (attrition bias)	Low risk	343/393 completed. The last observation carried forward method to impute missing observations, excluding data asked the rescue therapy was used. All drop outs and losses to follow-up accounted for.
Selective reporting (reporting bias)	Low risk	All the prespecified clinically relevant outcomes reported.
Other bias	Unclear risk	This study was funded by Bristol-Myers Squibb and Astrazeneca. Editorial and writing assistance was funded by Bristol-Myers Squibb and Astrazeneca. Some of the trialists are employees of Bristol-Myers Squibb.

Kadowaki 2014

**Methods**	12-week, randomized, double-blind, placebo-controlled, parallel-group, dose-finding phase II trial, in 32 centers in Japan.
**Participants**	547 initially randomized: empagliflozin 5mg n= 10, 10mg n=110, 25mg n=109, 50 mg n=110, placebo (n=109)
**Interventions**	Randomized (1:1:1:1:1) to receive once-daily oral empagliflozin 5, 10, 25, 50 mg, versus placebo as monotherapy for 12 weeks (oral dose).<br><br>Add on: diet and exercise counselling
**Outcomes**	At week 12.<br><br>**Primary outcomes of the trial**<br><br>change from baseline in HbA1c at week 12.<br><br>**Secondary outcomes of the trial**<br><br>percentage of patients with HbA1c ≥7.0% at baseline who achieved HbA1c <7.0%<br>change from baseline in fasting plasma glucose (FPG)<br>changes from baseline in body weight

	waist circumference<br>systolic (SBP) and diastolic (DBP) blood pressure<br>percentage of patients with >5% reduction in body weight<br>percentage of patients who had uncontrolled blood pressure at baseline who had controlled blood pressure (SBP <130 mmHg and DBP <80 mmHg)<br>changes from baseline in HbA1c at week 12 were analyzed in subgroups of patients with baseline HbA1c<8.0% and ≥8.0%.<br><br>**Adverse events and safety assessments**<br><br>clinical laboratory tests,<br>vital signs,<br>12-lead electrocardiogram,<br>physical examination,<br>use of rescue therapy<br>confirmed hypoglycemic AEs (plasma glucose ≤70 mg/dL (≤3.9 mmol/L) and/or assistance required<br>urinary tract infection (UTI)<br>genital infection<br>volume depletion
**Inclusion criteria**	men and women<br>aged ≥20 and ≤80 years<br>T2DM<br>BMI ≥18 and ≤40 kg/m^2<br>on a diet and exercise regimen<br>drug-naive (no antidiabetes agents for ≥10 weeks prior to consent)<br>HbA1c ≥7.0% and ≤10.0% at screening,<br>receiving 1 oral antidiabetes agent other than thiazolidinedione, glucagon-like peptide analogues or insulin (unchanged for ≥10 weeks prior to consent)
**Exclusion criteria**	uncontrolled hyperglycemia (plasma glucose (240 mg/dL (13.3 mmol/L) after an overnight fast during the washout/placebo run-in period, confirmed by a second measurement),<br>moderate or severe renal impairment (eGFR)\60 mL/min/1.73 m^2 using the Japanese GFR estimation<br>liver disease (serum alanine aminotransferase, aspartate aminotransferase, or alkaline phosphatase >3 x upper limit of normal)<br>acute coronary syndrome<br>a stroke or transient ischemic attack within 12 weeks of consent<br>anti-obesity drugs within 12 weeks of consent<br>bariatric surgery within 2 years, systemic steroids<br>change in the dose of thyroid hormones

	uncontrolled endocrine disorder other than T2DM<br>women of child-bearing potential were required to use adequate contraception
**Notes**	ClinicalTrials.gov identifier NCT01193218.<br><br>There is a 40-week randomized extension period (EMPA Kadowaki 2015), in which patients who received empagliflozin 10 or 25 mg in the first 12-week treatment period continued this treatment, and patients treated with placebo, empagliflozin 5 or 50 mg in the first 12-week treatment period were switched to empagliflozin 10 or 25 mg. The results from this extension period are not included in our analyses.

Risk of bias table

**Bias**	**Authors' judgement**	**Support for judgement**
Random sequence generation (selection bias)	Low risk	Computer-generated random sequence and an interactive web response system, stratified by previous antidiabetes medication (sulfonylurea, non-sulfonylurea, none), HbA1c levels and renal function.
Allocation concealment (selection bias)	Unclear risk	Not described in detail but implied in the computer-generated random sequence.
Blinding of participants and personnel (performance bias)	Low risk	Double-blind.
Blinding of outcome assessment (detection bias)	Low risk	Double-blind.
Incomplete outcome data (attrition bias)	Low risk	528/547 completed. All drops outs and losses to follow up accounted for.
Selective reporting (reporting bias)	Low risk	All clinical relevant outcomes are defined and reported.
Other bias	Unclear risk	This study was funded by Boehringer Ingelheim, Ingelheim, Germany and Eli Lilly, Indianapolis, USA. Page processing charges for this manuscript were

		covered by Boehringer<br>Ingelheim, Ingelheim, Germany. Medical writing assistance was funded by Boehringer Ingelheim. Some of the trialists are employees of Boehringer Ingelheim.

Kaku 2013

**Methods**	12-week multicenter, randomized, five-arm, parallel-group, double-blind, placebo-controlled study conducted at 26 centers in Japan.
**Participants**	279 initially randomized: placebo n=54; or dapagliflozin (1 mg n=59; 2.5mg n=56; 5mg n=58; 10 mg n=52).
**Interventions**	Placebo or dapagliflozin (1, 2.5, 5 or 10mg) daily oral dose.<br><br>Add on: none.
**Outcomes**	**Primary outcomes of the trial**<br><br>change in HbA1c from baseline to week 12.<br><br>**Secondary outcomes of the trial**<br><br>change from baseline in FPG at week 12,<br>proportion of patients achieving HbA1c <7.0% at week 12,<br>proportion of patients achieving a therapeutic response (>0.5% placebo-corrected reduction in HbA1c or <7.0% HbA1c),<br>change from baseline in body weight,<br>area under the curve from 0 to 180min (AUC0–180 min) for postprandial glucose; urinary glucose excretion (UGE) and UGE/creatinine ratio; fasting insulin,<br>C-peptide response to an oral glucose tolerance test (OGTT),<br>β-cell function (HOMA-2),<br>insulin resistance (as measured by HOMA-2).<br><br>**Adverse events and safety assessments**<br><br>adverse events (AEs),<br>laboratory variables,<br>physical examination,<br>electrocardiogram,<br>vital signs,<br>urinary infections<br>genital infections

	hypoglycemic and hyperglycemic events.
**Inclusion criteria**	men and women<br>Japanese<br>18 – 79 years (mean age, years (SD) placebo 58.4 (10.0); 1mg 55.9 (9.7); 2.5mg 57.7 (9.3); 5mg 58.0 (9.5); 10mg 56.5 (11.5))<br>T2DM<br>were strictly treatment naive – defined as never having received medications for diabetes (insulin and/or OADs); or were relatively treatment naive – defined as having received medications for diabetes for less than 30 days since diagnosis and who, during the 30 day period prior to enrolment, did not receive OADs for more than 3 consecutive or 7 non- consecutive days and HbA1c ≥7.0% and ≤10% at the enrolment visit; and were HbA1c ≥7.0% and ≤10% at the enrolment visit,<br>were treated with medications for diabetes but had not been treated within 6 weeks of enrolment,<br>were treated up to enrolment with a single OAD agent or less than a half-maximal dose of 2 OAD agents, HbA1c ≤8% at the enrolment visit, and fasting plasma glucose (FPG) ≤13.3 mmol/l (240 mg/dl) at the enrolment visit,<br>fasting C-peptide >1.0 ng/ml (0.33 nmol/l),<br>body mass index ≤40 kg/m^2,<br>serum creatinine (Scr) <1.5 mg/dl (132.6 µmol/l) for men and <1.4 mg/dl (123.8 µmol/l) for women and glomerular filtration rate>60 ml/min/1.73m2 (calculated by the modification of diet in renal disease formula.
**Exclusion criteria**	chronic insulin therapy within 30 days before the enrolment visit (previous acute (≤2 weeks) intermittent use of insulin was allowed, as long as the last dose of insulin was administered more than 2 weeks before the enrolment visit or for the treatment of gestational diabetes), FPG >13.3mmol/l (240mg/dl) at weeks -10, -6, -4 or -1 for patients treated with OADs up to enrolment,<br>cardiovascular events,<br>unstable renal disease,<br>retinopathy,<br>hepatic disease,<br>hematologic disease.
**Notes**	ClinicalTrials.gov identifier: NCT00972244

Risk of bias table

Bias	Authors' judgement	Support for judgement
Random sequence generation (selection bias)	Low risk	A computer-generated randomization list was used, based on a permuted block method via an interactive voice response system. A computer-generated randomization list was used, based on a permuted block method, to allocate patients to placebo or dapagliflozin (1, 2.5, 5 or 10 mg) daily.
Allocation concealment (selection bias)	Unclear risk	Not stated, but implied as patients were randomized via an interactive voice response system.
Blinding of participants and personnel (performance bias)	Low risk	Described as double-blind.
Blinding of outcome assessment (detection bias)	Low risk	Described as double-blind.
Incomplete outcome data (attrition bias)	Low risk	258/279 completed. Analysis of efficacy variables was made using the full analysis set (FAS). The FAS included all randomized patients who received at least one dose of study medication, and who had a non-missing baseline value and at least one post-baseline efficacy value for at least one efficacy variable. The safety analysis set included all randomized patients who received at least one dose of study medication. All drop outs and losses to follow up accounted for.
Selective reporting (reporting bias)	Low risk	All clinically relevant outcomes were reported
Other bias	Unclear risk	Sponsored by Bristol-Myers Squibb and AstraZeneca. Medical writing assistance funded by Bristol-Myers Squibb and AstraZeneca.

Kaku 2014

**Methods**	24-week multicenter, randomized, five-arm, parallel-group, double-blind, placebo-controlled study conducted in Japan.

**Participants**	261 initially randomized: placebo n=87; 5,g n=86; 10mg n=88
**Interventions**	Placebo or dapagliflozin (5 or 10 mg) once daily for 24 weeks<br>Add on: not stated
**Outcomes**	**Primary outcomes of the trial**<br>change in HbA1c from baseline to week 12.<br>**Secondary outcomes of the trial**<br>change from baseline to week 24 in total body weight in patients with baseline body mass index (BMI) ≥25 kg/m2,<br>fasting insulin and C-peptide levels,<br>seated systolic blood pressure (SBP) overall and in patients with baseline seated SBP ≥130mmHg,<br>fasting lipids (total cholesterol, low-density lipoprotein cholesterol, high-density lipoprotein cholesterol, free fatty acid and triglyceride levels),<br>proportion of patients achieving a therapeutic glycemic response (defined as HbA1c <7%) after 24weeks in patients with baseline HbA1c ≥7%,<br>proportion of patients discontinued for lack of efficacy or rescued for failing to maintain FPG below prespecified rescue criteria after 24weeks.<br>**Adverse events and safety assessments**<br>reported adverse events (AEs),<br>laboratory values,<br>ECG, heart rate,<br>blood pressure,<br>hypoglycemic events,<br>calculated creatinine clearance, eGFR,<br>physical examination findings.
**Inclusion criteria**	≥20 years<br>T2DM<br>drug-naive (never received medical treatment for diabetes or received treatment for <30 days after diagnosis, and during the 30-day period before screening did not receive oral antidiabetic agents for >3 consecutive or >7 non-consecutive days, or were previously treated for diabetes but not within 6 weeks of enrolment),<br>or receiving ongoing treatment for diabetes within 6 weeks of enrolment (not drug-naive).

	HbA1c values ≥6.5 and ≤10% for patients defined as drug-naive, and HbA1c values ≤8% for patients with ongoing treatment.<br>1 week before randomization, HbA1c ≥6.5 and ≤10% inadequately controlled by diet and exercise.
**Exclusion criteria**	type 1 diabetes or FPG >240mg/dl (13.3mmol/l)<br>pregnant or breastfeeding women,<br>creatinine kinase >3× upper limit of normal (ULN),<br>eGFR <45 ml/min,<br>serum creatinine value of >1.5mg/dl (>133 µmol/l) for men and >1.4mg/dl (>124 µmol/l) for women,<br>severe hepatic insufficiency and/or significant abnormal liver function (aspartate aminotransferase >3 ×ULN and/or alanine aminotransferase >3 × ULN),<br>New York Heart Association class IV congestive heart failure; unstable or acute congestive heart failure,<br>treatment with thiazolidinediones <6 months before enrolment.
**Notes**	No ClinicalTrials.gov identifier specified.

Risk of bias table

**Bias**	**Authors' judgement**	**Support for judgement**
Random sequence generation (selection bias)	Unclear risk	Not described, described as randomized.
Allocation concealment (selection bias)	Unclear risk	Not described, described as randomized no details of allocation concealment procedure.
Blinding of participants and personnel (performance bias)	Low risk	Described as double-blind
Blinding of outcome assessment (detection bias)	Low risk	Described as double-blind
Incomplete outcome data	Low risk	239/262 completed.

(attrition bias)		Analysis of efficacy variables was made using the full analysis set (FAS). The FAS included all randomized patients who received at least one dose of study medication, and who had a non-missing baseline value and at least one post-baseline efficacy value for at least one efficacy variable. The safety analysis set included all randomized patients who received at least one dose of study medication. FAS used last observation carried forward (LOCF). All drop outs and losses to follow up accounted for.<br><br>The safety set comprised patients who took ≥1 dose of double-blind study medication and who provided any safety records.
Selective reporting (reporting bias)	Low risk	All relevant outcomes seem to have been reported
Other bias	Unclear risk	The study was funded by AstraZeneca and Bristol-Myers Squibb. Medical writing assistance was funded by AstraZeneca and Bristol-Myers Squibb.

Kovacs 2014

**Methods**	76-week, randomized, placebo-controlled trial, at 69 centers in eight countries
**Participants**	499 initially randomized: empagliflozin 10mg n=165; 25 mg n=168; placebo n=165
**Interventions**	Empagliflozin (10mg and 25 mg once daily, oral dose) versus placebo<br><br>Add on: pioglitazone alone or pioglitazone plus metformin
**Outcomes**	**Primary outcomes of the trial**<br><br>change from baseline in HbA1c at week 24.<br><br>**Secondary outcomes of the trial**<br><br>change from baseline in fasting plasma glucose (FPG)<br>change from baseline in body weight<br>HbA1c and FPG<br>percentage with HbA1c ≥7% at baseline who achieved HbA1c <7% at week 24;<br>percentage of patients achieving a >5% reduction in body weight at

	week 24;<br>changes from baseline in waist circumference,<br>SBP and diastolic blood pressure (DBP) at week 24<br>percentage of patients with uncontrolled blood pressure at baseline who had controlled blood pressure (SBP <130mmHg and DBP<80 mmHg) at week 24.<br>change from baseline in HbA1c at week 24 was analyzed in subgroups of patients receiving background therapy of pioglitazone alone and pioglitazone plus metformin. Use of rescue therapy was<br>UGE was not measured in this study.<br><br>**Adverse events and safety assessments**<br><br>clinical laboratory tests<br>vital signs,<br>12-lead electrocardiogram (ECG)<br>physical examination.<br>confirmed hypoglycemic AEs (plasma glucose ≤3.9 mmol/l and/or requiring assistance),<br>urinary tract infection (UTI)<br>genital infection<br>in patients receiving pioglitazone: a dedicated examination for signs and symptoms of heart failure and edema was performed 6 weeks after randomization, in addition to the standard physical examination performed during the placebo run-in and at week 24.
**Inclusion criteria**	men and women<br>aged ≥18 years (and ≤65 years in India)<br>T2DM<br>BMI ≤45 kg/m^2<br>HbA1c ≥7 and ≤10% at screening<br>on a diet and exercise regimen<br>for ≥12 weeks prior to randomization, had been receiving unchanged doses of pioglitazone monotherapy (≥30 mg/day, or the maximum tolerated dose, or the maximum dose according to the local label) or pioglitazone plus metformin (≥1500 mg/day, or maximum tolerated dose or maximum dose according to the local label).
**Exclusion criteria**	uncontrolled hyperglycemia (plasma glucose >13.3 mmol/l after an overnight fast, confirmed by a second measurement),<br>severe renal impairment (estimated glomerular filtration rate (eGFR) <30 ml/min/1.73m2 using the Modification of Diet in Renal Disease (MDRD) equation),<br>contraindication to pioglitazone and/or metformin according to the local label,

	indication of liver disease (serum alanine aminotransferase, aspartate aminotransferase or alkaline phosphatase >3×upper limit of normal)<br>acute coronary syndrome, stroke or a transient ischemic attack within 3months of consent,<br>anti-obesity drugs<br>bariatric surgery in past 2 years<br>systemic steroids<br>change in the dose of thyroid hormones<br>uncontrolled endocrine disorder except T2D2
**Notes**	ClinicalTrials.gov identifier: NCT01210001<br>Empagliflozin vs placebo in patients on pioglitazone or pioglitazone plus metformin. Empagliflozin 10 mg arm not included as this low dose was outside our inclusion criteria.<br>Double blind 52-week extension trial (i.e. 76 weeks in total) results (Kovacs 2015) included in these analyses.<br>A non-randomized extension (NCT01289990) was also conducted; these data are not included in our analyses.<br>"A dedicated Phase 3 trial (NCT01370005) will provide further information on the effects of empagliflozin on BP."

Risk of bias table

Bias	Authors' judgement	Support for judgement
Random sequence generation (selection bias)	Low risk	Computer-generated random sequence and an interactive voice and web response system
Allocation concealment (selection bias)	Unclear risk	Not described in detail but random sequence was computer-generated.
Blinding of participants and personnel (performance bias)	Low risk	Double blind placebo controlled.
Blinding of outcome assessment (detection bias)	Low risk	Double blind placebo controlled.

Incomplete outcome data (attrition bias)	Low risk	457/499 initially randomized completed (all 499 contributed data to the final analysis set FAS). Last observation carried forward. All drops outs and losses to follow up accounted for.
Selective reporting (reporting bias)	Low risk	All clinically relevant outcomes are defined and reported.
Other bias	Unclear risk	This study was funded by Boehringer Ingelheim. Medical writing assistance, supported financially by Boehringer Ingelheim.

Lambers Heerspink 2013

**Methods**	12-week randomized, placebo-controlled, double-blind, multicenter, three-arm, parallel-group, study conducted in Canada, The Netherlands and the USA.
**Participants**	75 initially randomized: placebo (n=25); dapagliflozin (n=24); hydrochlorothiazide (n=26)
**Interventions**	Eligible patients (who met the inclusion criteria and adhered to study medication) were randomly assigned to dapagliflozin 10 mg/day, hydrochlorothiazide 25 mg/day, or matched placebo in a 1:1:1 ratio (oral dose) for 12 weeks.<br>Add on: metformin, SU
**Outcomes**	**Primary outcomes of the trial**<br>The study was exploratory in nature and designed as a mechanism-of-action study to assess the effects of dapagliflozin on renal function, BP and circulating plasma volume,<br>24-h ambulatory BP monitoring,<br>GFR was determined by plasma disappearance of iohexol,<br>plasma volume and red cell mass.<br>**Secondary outcomes of the trial**<br>blood pressure, body weight and plasma volume.<br>**Adverse events and safety assessments**<br>adverse events,<br>urinary tract infection,<br>genital infection,

	hypoglycemia,<br>other adverse events including dizziness, syncope,<br>discontinuation of study medication to adverse event.
**Inclusion criteria**	men and women<br>T2DM<br>aged between 18 and 70 years<br>inadequate glycemic control, defined as HbA1c ≥6.6% and ≤9.5%<br>receiving a stable dose of metformin and/or a sulfonylurea derivative for at least 4weeks prior to study entry.<br>C-peptide ≥ 0.27 nmol/l,<br>eGFR >60 ml/min/1.73m2 and <150 ml/min/1.73m^2,<br>urine albumin:creatinine ratio <300 mg/g,<br>body mass index ≤ 45.0 kg/m^2,<br>inadequate BP control, defined as systolic blood pressure (SBP) ≥ 130 and <165 mmHg, and/or diastolic BP ≥ 80 and <105 mmHg.
**Exclusion criteria**	type 1 diabetes<br>cardiovascular disease within 6 months of study entry,<br>pregnant women<br>history of adverse reaction to radiocontrast dye,<br>allergy to or contraindication for thiazide diuretics.
**Notes**	Participants in the placebo group were slightly older than those in the other two treatment groups.<br>ClinicalTrials.gov identifier: NCT00976495

Risk of bias table

**Bias**	**Authors' judgement**	**Support for judgement**
Random sequence generation (selection bias)	Low risk	Study treatments were allocated using a central, computer-based randomization procedure.
Allocation concealment (selection bias)	Unclear risk	Not stated, but implied as study treatments were allocated using a central, computer-based randomization procedure.
Blinding of participants and personnel	Low risk	Blinding of patients and investigators to study treatment was achieved using a double-dummy technique.

(performance bias)		
Blinding of outcome assessment (detection bias)	Low risk	Not described if all outcome assessments were blinded (e.g. coding of adverse events), but likely they were as study describes the use of MedDRA terms and study described as double blinded.
Incomplete outcome data (attrition bias)	Low risk	74/75 completed (1 withdrew consent). If no week 12 measurement was available, the last available post-baseline measurement was used for analysis of 24-h, day-time and night-time BP, and the last available post-baseline measurement after week 4 was used for analysis of GFR, plasma volume and red cell mass.
Selective reporting (reporting bias)	Unclear risk	Due to the exploratory nature of the study, no statistical comparisons between treatment arms were made. Median changes in 24-h ambulatory BP, plasma volume and red cell mass are reported in the sub study because of the small sample size of the sub study and the fact that mean changes can be driven by a few extreme values.
Other bias	Unclear risk	The trial was sponsored by Bristol-Myers Squibb and AstraZeneca. Some of the trialists are employees of Bristol-Myers Squibb.

Leiter 2014

**Methods**	52-week, multicenter, randomized, double-blind, age-stratified, placebo-controlled study in 173 centers in 10 countries United States, Canada, Australia, Chile, Argentina, and five European countries.
**Participants**	964 initially randomized: placebo n=483; dapagliflozin n=482
**Interventions**	Randomized 1:1 to receive once-daily dapagliflozin 10 mg or matched placebo for 24 weeks.<br><br>participants reduced their average daily insulin dose by 25% at randomization<br>rescue medication permitted and rescue criteria became stricter during the course of the study, as follows: weeks 0–4, FPG >270 mg/dL; weeks 5–8, FPG >240 mg/dL; weeks 9–24, FPG >200 mg/dL; week 25 onward, HbA1c >8.0%.<br>antihypertensive rescue (choice of medication at investigator discretion) was given after repeated confirmed measurements of

	blood pressure.<br><br>Add on: pre-existing, stable background treatment of antidiabetic drugs, including insulin, i.e. participants continued study treatment and concomitant medications.
**Outcomes**	**Primary outcomes of the trial**<br><br>Glycemic efficacy at 24 weeks<br>mean change in HbA1c from baseline and proportion of participants achieving a three item outcome measure of combined clinical benefit: simultaneous HbA1c decrease of 0.5% or greater, total body weight reduction of 3% or greater, and systolic BP (SBP) reduction of 3 mmHg or more from baseline.<br><br>**Secondary outcomes of the trial**<br><br>mean percent change in total body weight (BW) from baseline to week 24, the proportion of patients with a baseline body mass index ≥27 kg/m^2 achieving a reduction in BW of ≥5% at week 24, seated systolic BP at weeks 8 and 24, and seated systolic BP in patients with a baseline seated systolic BP≥130 mmHg.<br>mean change from baseline in diastolic BP overall and in patients with seated baseline systolic BP≥130 mmHg at weeks 8 and 24;<br>mean change in seated systolic BP in patients who had baseline systolic BP≥130 mmHg at week 24; mean change in BW from baseline to week 24;<br>pulse<br>change in hemoglobin A1c (HbA1c) in patients with baseline HbA1c ≥8.0% and HbA1c ≥9.0% at week 24;<br>change in FPG at week 1 and week 24;<br>change in calculated average daily insulin dose in patients treated with insulin at baseline at week 24;<br>change in plasma uric acid levels at week 24.<br>proportion with seated systolic BP<130 mmHg at week 24 in the population with seated systolic BP≥130 mmHg at baseline;<br>rescue for failing to maintain FPG below prespecified rescue criteria with dapagliflozin 10 mg versus placebo at weeks 4, 8, 16, and 24;<br>failure to maintain BP (systolic and diastolic) below prespecified rescue criteria with dapagliflozin 10 mg versus placebo at weeks 8, 16, and 24;<br>reduction from baseline of 3 mmHg or more and 5 mmHg or more in seated systolic BP at week 24;<br>reduction from baseline of 3 mmHg or more and 5 mmHg or more in seated systolic BP at week 24;<br>proportion of patients achieving a therapeutic response, defined as

	a reduction in HbA1c of 0.5% or more, at week 24.<br><br>**Adverse events and safety assessments**<br><br>urinary tract infection<br>genital infection<br>discontinuations due to AEs,<br>prespecified laboratory abnormalities<br>renal impairment and failure<br>changes in creatinine renal clearance and blood creatinine<br>kidney infection<br>hypotension, dehydration, or hypovolemia<br>syncope<br>circulatory collapse<br>increases in hematocrit<br>liver function tests<br>glomerular function
**Inclusion criteria**	men and women<br>older adults "Participants aged 75 and older (7.7% of total) were distributed as follows: 46 participants in the dapagliflozin group (9.6%) and 28 in the placebo group (5.8%)" Mean age (SD) placebo 63.6 (7.0); dapagliflozin 63.9 (7.6)<br>T2DM (HbA1c 7.0–10.0%)<br>preexisting cardiovascular disease (defined as prior documented coronary heart disease, including history of myocardial infarction or revascularization or coronary artery stenosis >50%, confirmed with angiography or abnormal stress test imaging, compatible with ischemias or prior myocardial infarction; or prior documented stroke or transient ischemic attack; or (3) prior documented peripheral artery disease treated with revascularization (excluding amputation).
**Exclusion criteria**	type 1 diabetes mellitus,<br>use of rosiglitazone or three or more oral antihyperglycemic drugs,<br>symptoms of poorly controlled diabetes such as marked polyuria, polydipsia, and/or >10% weight loss,<br>fasting plasma glucose (FPG) >270 mg/dL,<br>cardiovascular events within 2 months of enrolment,<br>New York Association class IV congestive heart failure,<br>unstable or acute congestive heart failure,<br>systolic blood pressure (BP) ≥160 mmHg and/or diastolic BP ≥100 mmHg at randomization,<br>calculated creatinine clearance <60 mL/min; urine albumin:creatinine ratio >1,800 mg/g,

	history of unstable or rapidly progressing renal disease.
**Notes**	ClinicalTrials.gov identifier: NCT01042977<br><br>"Participants aged 75 and older (7.7% of total) were distributed as follows: 46 participants in the dapagliflozin group (9.6%) and 28 in the placebo group (5.8%)"<br><br>"Demographic and baseline characteristics of participants were well balanced between treatments (Table 1) and age strata"<br><br>Trial report includes 28- and 52-week extension periods. The primary efficacy analysis was conducted at week 24, with exploratory efficacy outcomes at week 52.

Risk of bias table

**Bias**	**Authors' judgement**	**Support for judgement**
Random sequence generation (selection bias)	Low risk	Patients were assigned a unique enrolment number using Interactive Web Response System (IWRS) or Interactive Voice Response System (IVRS) at visit1 (enrolment visit). Patients were randomized to the treatment groups using the method of randomly permuted blocks with a block size of four after evaluation for all inclusion/exclusion criteria. Patients were stratified into one of the eightage-by-insulin use-by-time from most recent qualifying cardiovascular event strata according to age group (<65 years vs≥65 years at enrolment), use of insulin (no vs. yes at randomization) and time from most recent qualifying cardiovascular event (more than 1 year vs1 year or less (i.e., within 12 months) before enrolment). Patients were given medication with the bottle number allocated by the IWRS/IVRS.
Allocation concealment (selection bias)	Unclear risk	Not stated, but implied by use of computerized system, as described for randomization.
Blinding of participants and personnel (performance bias)	Low risk	Double-blind.
Blinding of	Low risk	Double-blind.

outcome assessment (detection bias)		
Incomplete outcome data (attrition bias)	Unclear risk	870/964 completed. All drop outs and losses to follow up accounted for. Published report states that 962 comprised the full analysis set. 965 started double-blind placebo treatment in total (i.e. one more than the number randomized)
Selective reporting (reporting bias)	Low risk	All the prespecified clinically relevant outcomes reported.
Other bias	Unclear risk	Funded by AstraZeneca and Bristol-Myers Squibb. The sponsors had no part in the study and design. AstraZeneca and Bristol-Myers Squibb, provided editorial support.

Lewin 2015

**Methods**	52-week phase 3, randomized, double-blind, parallel-group study in 197 centers in 22 countries.
**Participants**	677 initially randomized: empagliflozin 25 mg/linagliptin 5 mg (n = 137), empagliflozin 10 mg/linagliptin 5 mg (n = 136), empagliflozin 25 mg (n = 135), empagliflozin 10 mg (n = 134), or linagliptin 5 mg (n = 135)
**Interventions**	Randomized (1:1:1:1:1) to receive empagliflozin 25 mg/linagliptin 5 mg, empagliflozin 10 mg/linagliptin 5 mg, empagliflozin 25 mg, empagliflozin 10 mg, or linagliptin 5 mg for 52 weeks. Once daily oral dose.<br>Add on: diet and exercise.<br>Rescue medication permitted in case of hyperglycemia.
**Outcomes**	**Primary outcomes of the trial**<br>change from baseline in HbA1c at week 24.<br>**Secondary outcomes of the trial**<br>change from baseline in FPG at week 24,<br>change from baseline in weight at week 24<br>proportion of subjects with baseline HbA1c ≥7% ($53 mmol/mol) who had HbA1c <7% (<53 mmol/mol) at week 24. Exploratory end

	points were as follows:<br>changes from baseline in HbA1c at week 24 in subgroups with HbA1c ≥8.5% (≥69 mmol/mol) and <8.5% (<69 mmol/mol) at baseline<br>changes from baseline in HbA1c, FPG,<br>weight,<br>systolic BP (SBP), and diastolic BP (DBP) at week 52;<br>proportion of subjects with baseline HbA1c ≥7% (≥53 mmol/mol) who had HbA1c <7% (<53 mmol/mol) at week 52.<br><br>**Adverse events and safety assessments**<br><br>vital signs,<br>clinical laboratory parameters,<br>adverse events including all events with an onset after the first dose and up to 7 days after the last dose of study medication.<br>confirmed hypoglycemic events<br>urinary tract infection (UTI),<br>genital infection,<br>volume depletion,<br>hypersensitivity reactions,<br>pancreatitis.
**Inclusion criteria**	men and women,<br>≥18 years,<br>T2DM,<br>BMI ≤45 kg/m^2,<br>HbA1c >7% to ≤10.5% (>53 to <91 mmol/mol) at screening despite a diet and exercise regimen,<br>not received treatment with oral antidiabetes therapy, GLP-1 analogue, or insulin for ≥12 weeks prior to randomization.
**Exclusion criteria**	uncontrolled hyperglycemia (glucose level .240 mg/dL after an overnight fast during the placebo run-in,<br>eGFR >60 mL/min/1.73 m2 (using the MDRD equation)<br>acute coronary syndrome, stroke, or transient ischemic attack within 3 months prior to consent;<br>bariatric surgery in the last 2 years<br>treatment with anti-obesity drugs within 3 months prior to consent.
**Notes**	ClinicalTrials.gov identifier: NCT01422876<br><br>Initial treatment was with empagliflozin for 24 weeks, extended to 52 weeks.

Risk of bias table

Bias	Authors' judgement	Support for judgement
Random sequence generation (selection bias)	Unclear risk	Randomized but not described fully in the published report of the study.
Allocation concealment (selection bias)	Unclear risk	Not described.
Blinding of participants and personnel (performance bias)	Low risk	Double-blind.
Blinding of outcome assessment (detection bias)	Low risk	Double-blind.
Incomplete outcome data (attrition bias)	Low risk	572/677 subjects completed 52 weeks treatment. 667 comprised the analysis data set. All drops outs and losses to follow up accounted for.
Selective reporting (reporting bias)	Low risk	All clinically relevant outcomes are defined and reported.
Other bias	Unclear risk	This study was funded by Boehringer Ingelheim and Eli Lilly and Company. The trialists has served on scientific advisory boards for, or are employees of, Boehringer Ingelheim<br>Medical writing assistance was supported financially by Boehringer Ingelheim.

List 2009

**Methods**	12-week, randomized, parallel-group, double-blind, placebo-controlled study 98 clinical centers in the U.S., 24 in Canada, 8 in Mexico, and 3 in Puerto Rico.
**Participants**	389 initially randomized: 5 mg dapagliflozin n = 58; 10 mg dapagliflozin n = 47; 20 mg dapagliflozin n = 59; 50 mg dapagliflozin n= 56; metformin n = 56
**Interventions**	Randomly assigned equally to once-daily dapagliflozin (2.5, 5, 10, 20,

	or 50 mg), metformin XR (750 mg force-titrated at week 2 to 1,500 mg) (therapeutic benchmark), or placebo. Daily for x12weeks (oral dose).<br><br>Add on: none.<br><br>Use of rescue medication, not described in the published study report.
**Outcomes**	**Primary outcomes of the trial**<br><br>mean A1C change from baseline after 12 weeks.<br><br>**Secondary outcomes of the trial**<br><br>FPG change from baseline,<br>dose dependent trends in glycemic efficacy, proportion of patients achieving A1C ≤7%,<br>change in 24-h urinary glucose- to-creatinine ratio<br>total weight loss.<br><br>**Adverse events and safety assessments**<br><br>vital signs,<br>brief physical examination,<br>adverse event assessment at each visit.<br>complete physical examination and electrocardiograms<br>adverse events<br>safety topics of special interest were summarized by interest categories. e.g. urinary tract infections
**Inclusion criteria**	men and women<br>aged 18–79<br>T2DM<br>A1C ≥7% and ≤10%<br>fasting C peptide >1.0 ng/ml,<br>BMI ≤40 kg/m^2,<br>renal status: glomerular filtration rate >60 ml/min per 1.73 m^2,<br>serum creatinine <1.5 mg/dl (men)/<1.4mg/dl (women), and urine microalbumin/ creatinine ratio ≤300 mg/g.
**Exclusion criteria**	No exclusion criteria were described in the published report.
**Notes**	ClinicalTrials.gov identifier: NCT00263276<br><br>Three arms; dapagliflozin vs placebo, dapagliflozin vs. metformin.

	Baseline demographics and disease characteristics were similar among all groups

Risk of bias table

Bias	Authors' judgement	Support for judgement
Random sequence generation (selection bias)	Unclear risk	Not reported.
Allocation concealment (selection bias)	Unclear risk	Not reported.
Blinding of participants and personnel (performance bias)	Low risk	Double blind.
Blinding of outcome assessment (detection bias)	Low risk	Double blind.
Incomplete outcome data (attrition bias)	Low risk	348/389 348 completed week 12, and 41 discontinued. Last observation carried forward.
Selective reporting (reporting bias)	Unclear risk	All clinical relevant outcomes are defined and reported, however it is unclear which secondary or exploratory outcomes were of interest as these were not all described prospectively in the published report of the study results.
Other bias	Unclear risk	This study was supported by Bristol-Myers Squibb and Astra- Zeneca. Editorial support was funded by Bristol-Myers Squibb.

Mathieu 2015

**Methods**	24-week, multicenter, randomized, double-blind, placebo-controlled, parallel-group, phase 3 study in multiple centers across 7 countries

**Participants**	320 initially randomized: placebo n = 160, dapagliflozin 10 mg n = 160.
**Interventions**	Randomized (blocked 1:1 ratio within each stratum by HbA1c level) to receive once-daily dapagliflozin 10 mg/day plus saxagliptin and metformin, or placebo.<br>Add on: saxagliptin 5mg/day and metformin.<br>Rescue mediation permitted in case of hyperglycemia.
**Outcomes**	**Primary outcomes of the trial**<br>mean change from baseline in HbA1c at week mean change from baseline in HbA1c level after 24 weeks of double-blind treatment with dapagliflozin versus placebo add-on to saxagliptin plus metformin.<br>**Secondary outcomes of the trial**<br>mean change from baseline at 24 weeks in FPG level, 2-h postprandial glucose (PPG) level following a liquid meal tolerance test (MTT),<br>body weight,<br>mean proportion of patients achieving a therapeutic glycemic response, defined as an HbA1c level of <7.0% (53 mmol/mol), after 24 weeks,<br>proportion of patients rescued or discontinued from the study for lack of efficacy,<br>change from baseline in the PPG area under the concentration-time curve from 0 to 180 min (AUC0–180 min) during a liquid MTT,<br>change from baseline in serum lipid levels.<br>**Adverse events and safety assessments**<br>adverse events (AEs),<br>hypoglycemia, (major, requiring assistance and minor plasma glucose concentration of <63 mg/dL,<br>vital signs,<br>severe cutaneous events,<br>decreased lymphocyte count,<br>decreased thrombocyte count,<br>opportunistic infection,<br>pancreatitis,<br>pancreatic cancer,<br>fracture,<br>severe hypersensitivity,

	worsening renal function,<br>genital infections,<br>urinary tract infections,<br>bladder neoplasm,<br>breast neoplasm,<br>volume depletion,<br>heart failure.<br>suspected cardiovascular AEs,<br>liver injury.
**Inclusion criteria**	adults with T2DM and inadequate glycemic control<br>HbA1c level of 7.0–10.5% (53–91 mmol/mol)
**Exclusion criteria**	pregnancy,<br>cardiovascular events within 3 months of screening,<br>EGFR <60 mL/min/1.73 m^2<br>serum creatinine level of ≥1.5 mg/dL in men or ≥1.4 mg/dL in women,<br>microscopic hematuria with no known cause in men, significant hepatic disease.<br>on any antidiabetes medication, other than metformin and DPP-4 inhibitors, for >14 days during the 12 weeks before screening.<br>uncontrolled hypertension (systolic blood pressure ≥160 mm Hg and/or diastolic blood pressure ≥100 mm Hg) were allowed to enter the open-label period provided that their antihypertensive therapy was adjusted appropriately.
**Notes**	ClinicalTrials.gov identifier: NCT01646320<br>The results of a long-term extension will be presented in a subsequent report

Risk of bias table

**Bias**	**Authors' judgement**	**Support for judgement**
Random sequence generation (selection bias)	Low risk	Randomly assigned by an interactive voice response system in a centrally blocked 1:1 ratio within each stratum (by HbA1c level)
Allocation concealment (selection bias)	Unclear risk	Not described in detail but implied in computer generated random system.

Blinding of participants and personnel (performance bias)	Low risk	Double blind.
Blinding of outcome assessment (detection bias)	Low risk	Double blind.
Incomplete outcome data (attrition bias)	Low risk	301/320 completed (full analysis set; FAS). A last observation carried forward (LOCF) approach was used with terms for treatment group, stratum, and baseline value in the statistical model.
Selective reporting (reporting bias)	Low risk	All clinically relevant outcomes are defined and reported.
Other bias	Unclear risk	This study was funded by Bristol-Myers Squibb and AstraZeneca. Medical writing support was funded by AstraZeneca. Some of the study authors are employees of Bristol-Myers Squibb and AstraZeneca.

Matthaei 2015

**Methods**	52-week, international, multicenter, randomized, double-blind, parallel group, placebo-controlled, phase 3b study in 46 centers in 6 countries North America (Canada) and Europe (Czech Republic, Germany, Poland, Slovak Republic, and Spain)
**Participants**	218 initially randomized dapagliflozin 10 mg/day n = 109; or placebo n = 109
**Interventions**	Randomized 1:1 dapagliflozin 10mg once daily or matched placebo (oral dose)<br>Rescue medication permitted.<br>Add on: metformin, sulfonylurea
**Outcomes**	**Primary outcomes of the trial**<br>change from baseline to week 52 in HbA1c levels at 24 weeks<br>**Secondary outcomes of the trial**<br>change from baseline to week 52 in fasting plasma glucose (FPG)<br>total body weight,

	proportion of patients achieving a therapeutic glycemic response (defined as HbA1c <7.0% (53 mmol/mol))<br>change from baseline to week 52 in seated SBP<br>change from baseline to week 24 in fasting lipids (total cholesterol, LDL cholesterol, HDL cholesterol, LDL/HDL cholesterol ratio, and triglycerides)<br>C-peptide<br>proportion of patients who discontinued for lack of efficacy or were rescued for failing to maintain FPG below prespecified rescue criteria.<br><br>**Adverse events and safety assessments**<br><br>cardiovascular events,<br>laboratory<br>values,<br>electrocardiogram,<br>vital signs,<br>hypoglycemic events,<br>calculated creatinine<br>clearance,<br>estimated glomerular filtration<br>rate, and<br>physical examination<br>undesirable medical condition or the deterioration of a pre-existing medical condition<br>urinary tract infection<br>genital infection<br>hypoglycemia
**Inclusion criteria**	men<br>women who were not of childbearing potential<br>at least 18 years of age<br>T2DM<br>receiving a stable-dose combination therapy of metformin ≥1,500 mg/day<br>inadequate glycemic control (HbA1c ≥7.0% (53 mmol/mol) to ≤ 10.5% (91 mmol/ mol) at randomization).
**Exclusion criteria**	type 1 diabetes,<br>BMI ≥45.0 kg/m^2,<br>serum creatinine value of ≥1.5 mg/dL (133 mmol/L) for men or ≥1.4 mg/dL (124 mmol/L) for women,<br>unstable or rapidly progressing renal disease,<br>cardiovascular events<br>congestive heart failure (New York Heart Association Class IV),

	systolic BP (SBP) ≥160 mmHg, or diastolic BP (DBP) ≥100 mmHg
**Notes**	ClinicalTrials.gov identifier: NCT01392677<br><br>Matthaei 2015 is a 28-week extension of the original 24-week trial (Matthaei 2015a) (i.e. 52 weeks in total). We have included the data from the 28-week extension into our analysis.<br><br>Treatment groups were balanced with respect to demographics and diabetes-related baseline characteristics, with a higher proportion of women in the dapagliflozin treatment arm.

Risk of bias table

**Bias**	**Authors' judgement**	**Support for judgement**
Random sequence generation (selection bias)	Low risk	An interactive response system (web or voice based).
Allocation concealment (selection bias)	Unclear risk	Not described. Implied by centralized randomization.
Blinding of participants and personnel (performance bias)	Low risk	Placebo identical in size, color, smell, taste, packaging, and labelling.
Blinding of outcome assessment (detection bias)	Low risk	Placebo identical in size, color, smell, taste, packaging, and labelling.
Incomplete outcome data (attrition bias)	Unclear risk	202/219 completed. 1 participant died before randomization. 216/219 entered the full analysis set, which included all randomized patients who received at least one dose of study medication during the 24-week double-blind treatment period with a non-missing baseline value and one or more post-baseline value for at least one efficacy variable analyzed at week 24. **The intention-to-treat principle was preserved despite the exclusion of patients who took no study medication.** The primary safety analyses included all data regardless of

		rescue. All drops and losses to follow accounted for "Approximately 93% of the patients in each treatment arm completed the 24-week double-blind treatment period"
Selective reporting (reporting bias)	Low risk	All clinically relevant outcomes reported
Other bias	Unclear risk	This study was sponsored by Bristol-Myers Squibb and AstraZeneca. Medical writing assistance was funded by AstraZeneca

Nauck 2011

**Methods**	52-week randomized, double- blind, parallel-group, active-controlled, phase III, non-inferiority trial with a 156- week extension period ongoing at 95 sites in 10 countries (Argentina, France, Germany, U.K., Italy, Mexico, the Netherlands, South Africa, Spain, Sweden.
**Participants**	814 initially randomized. Dapagliflozin plus metformin n=406; glipizide plus metformin n=408
**Interventions**	Randomized in a 1:1 ratio to receive double-blind treatment with dapagliflozin or glipizide uptitrated to maximally tolerated doses (up to 10 and 20 mg, respectively). Rescue medication was permitted.<br>Daily oral dose for 52 weeks.<br>Add on: metformin
**Outcomes**	Assessed at week 52.<br>**Primary outcomes of the trial**<br>absolute change in HbA1c from baseline<br>**Secondary outcomes of the trial**<br>proportion of patients reporting at least one episode of hypoglycemia<br>proportion of patients achieving a TBW decrease ≥5% from baseline<br>change from baseline body weight in patients with a baseline BMI ≥30 kg/m^2 and in those with baseline BMI ≥27 kg/m^2,<br>waist circumference,<br>change in HbA1c in patients with an HbA1c of ≥7% at baseline

	FPG<br>proportions of patients with HbA1c <7% at week 52 in patients with baseline HbA1c ≥7% and proportions of patients with HbA1c ≤6.5% at week 52<br>absolute changes from baseline to week 52 for seated systolic and diastolic blood pressure,<br>percent changes from baseline to week 52 for total cholesterol, LDL cholesterol, HDL cholesterol triglycerides, and free fatty acids.<br><br>**Adverse events and safety assessments**<br><br>hypoglycemic events,<br>laboratory tests,<br>calculated creatinine clearance,<br>urinary glucose<br>creatinine ratio,<br>electrocardiographic and<br>physical examinations,<br>vital signs<br>genital infections and urinary tract infections.
**Inclusion criteria**	men and women<br>aged >18 years<br>inadequately controlled type 2 diabetes (HbA1c >6.5 and ≤10%), a maximum of 25% of randomized patients had a baseline HbA1c <7% while receiving metformin or metformin and one other OAD administered up to half-maximal dose for at least 8 weeks before enrolment.<br>fasting plasma glucose (FPG) ≤15mmol/L and C-peptide concentration of ≥0.33 nmol/L.
**Exclusion criteria**	Diabetes related: type 1 diabetes; diabetes insipidus; corticosteroid-induced type 2 diabetes; a history of diabetic ketoacidosis or hyperosmolar non-ketotic coma; poorly controlled diabetes characterized by polyuria/polydipsia with >10% weight loss; use of insulin within 1 year of enrolment, except in the case of hospitalization or use in gestational diabetes.<br>General: body mass index (BMI) >45.0 kg/m2,<br>calculated creatinine clearance <60 mL/min,<br>urine albumin:creatinine ratio >203.4 mg/mmol,<br>aspartate aminotransferase and/or alanine aminotransferase and/or creatine kinase ≥3x upper limit of normal range,<br>serum total bilirubin >34 µmol/L,<br>hemoglobin (Hb) ≤11 g/dL for men and ≤10 g/dL for women,<br>abnormal thyroid stimulating hormone level,<br>systolic blood pressure ≥180 mmHg and/or diastolic blood pressure

	≥110 mmHg,<br>cardiovascular event within 6 months of enrolment; congestive heart failure;<br>congenital renal glycosuria,<br>significant renal, hepatic, respiratory, hematological, oncological, endocrine, immunological (including hypersensitivity to study medications),<br>alcohol and/or substance misuse disorders,<br>pregnancy and/or lactation,<br>use of systemic corticosteroids equivalent to >10 mg of oral prednisolone within 30 days of enrolment, a history of bariatric surgery; and use of weight loss medication within 30 days of enrolment.
**Notes**	Only data from the 52-week double-blind treatment period are used in this analysis.<br><br>ClinicalTrials.gov identifier: NCT00660907

Risk of bias table

**Bias**	**Authors' judgement**	**Support for judgement**
Random sequence generation (selection bias)	Low risk	Patients were randomized sequentially at study level according to a predefined computer-generated randomization scheme provided by AstraZeneca.
Allocation concealment (selection bias)	Unclear risk	Not stated, but implied as allocation of study treatments was performed via an Interactive Web Response System in balanced block sizes of 4, to ensure approximate balance among treatment groups.
Blinding of participants and personnel (performance bias)	Low risk	Blinding of patients and investigators to study treatment was achieved using a double-dummy technique.
Blinding of outcome assessment (detection bias)	Low risk	Not described if all outcome assessments were blinded (e.g. coding of adverse events), but likely they were as study describes the use of MedDRA terms and study described as double blinded.
Incomplete outcome data	Low risk	622/814 completed the initial 52-week trial. Two analysis sets were defined: the safety analysis set, consisting of all

(attrition bias)		patients who received one or more doses of the investigational product, and the full analysis set, consisting of all randomized patients who received one or more doses of the investigational product and who had a non-missing baseline and one or more post-baseline efficacy value for one or more efficacy variable. Missing values at week 52 were replaced using the LOCF method. More participants did not complete in the glipizide plus metformin group.
Selective reporting (reporting bias)	Low risk	Primary, key secondary, and exploratory end points were analyzed using the full analysis set. Besides the major prespecified outcomes, a number of exploratory endpoints were assessed (but these were clinically relevant).
Other bias	Unclear risk	This study was sponsored by Bristol-Myers Squibb and AstraZeneca. Medical writing assistance was funded by AstraZeneca.

Ridderstråle 2013

**Methods**	104-week randomized, active-controlled, double-blind, phase 3 trial
**Participants**	1549 initially randomized, empagliflozin n=769; glimepiride n=780.
**Interventions**	1:1 ratio empagliflozin (25 mg once daily, orally) or glimepiride (1–4 mg once daily, oral dose) for 104 weeks.<br>Add on: metformin immediate release and diet and exercise.<br>Rescue treatment permitted in case of hyperglycemia.
**Outcomes**	**Primary outcomes of the trial**<br>change from baseline in HbA1c concentrations at weeks 52 and 104.<br>**Secondary outcomes of the trial**<br>changes from baseline in bodyweight,<br>systolic blood pressure, and diastolic blood pressure at weeks 52 and 104,<br>percentage of patients who received rescue therapy (increases in the dose of metformin or additional antidiabetes treatment) over 104 weeks,

	percentage of patients with HbA1c concentrations of at least 7% who achieved a level of less than 7%, percentage of patients with bodyweight reductions of more than 5%, and changes from baseline in fasting plasma glucose at weeks 52 and 104,<br>change from baseline in HbA1c concentration at week 104 in subgroup with baseline HbA1c of at least 8·5% and less than 8·5%,<br>lipids profiles cholesterol and triglycerides,<br>Substudies: sub-studies: changes from baseline in mean daily glucose at weeks 52 and 104 were assessed with eight-point glucose profiles; baseline in 2-h post-prandial glucose at weeks 52 and 104; trunk fat, limb fat, total fat mass, fat-free mass, abdominal visceral adipose tissue, and sub cutaneous adipose tissue.<br><br>**Adverse events and safety assessments**<br><br>confirmed hypoglycemic events (plasma glucose ≤3·9 mmol/L or requiring assistance) up to weeks 52 and 104,<br>vital signs,<br>clinical laboratory findings,<br>urinary tract infection, genital infection,<br>volume depletion.
**Inclusion criteria**	Adults (aged ≥18 years)<br>T2DM<br>BMI less than or equal to 45 kg/m²<br>HbA1c concentrations of ≥7 to 10%,<br>receiving an unchanged dose of metformin immediate release (≥1500 mg/day, maximum tolerated dose, or maximum dose according to the local label) for at least 12 weeks before randomization<br>If female: post-menopausal, or pre-menopausal and using appropriate contraception; not pregnant/breastfeeding.
**Exclusion criteria**	eGFR of less than 60 mL/min per 1·73 m^2 (Modified Diet Renal Disease formula)<br>blood glucose concentration >13·3 mmol/L after an overnight fast during the placebo run-in, confirmed by a second measurement,<br>use of antidiabetes drugs other than metformin immediate release any time during the 12 weeks before randomization.<br>bariatric surgery within 2 years; treatment with anti-obesity drugs within 3 months of screening; any treatment leading to unstable body weight<br>indication of liver disease (ALT, AST or alkaline phosphatase >3 x ULN) during screening or placebo run-in<br>cancer within 5 years (except basal cell carcinoma)

	acute coronary syndrome, stroke or transient ischemic attack within 3 months of informed consent<br>unstable red blood cells<br>treatment with systemic steroids<br>change in dose of thyroid hormones within 6 weeks of screening<br>any uncontrolled endocrine condition (except T2DM)<br>alcohol or drug abuse within 3 months of informed consent<br>taking an investigational drug within 30 days prior to receiving study drug
**Notes**	ClinicalTrials.gov identifier: NCT01167881.<br>52-week study, extended to 104 weeks.

Risk of bias table

**Bias**	**Authors' judgement**	**Support for judgement**
Random sequence generation (selection bias)	Low risk	The study sponsor did the randomization, stratified by HbA1c concentration at screening, eGFR, and region, using an interactive response system with a computer-generated random sequence.
Allocation concealment (selection bias)	Unclear risk	Not described, but implied in the computer-generated stratified randomization sequence technique.
Blinding of participants and personnel (performance bias)	Low risk	Patients and investigators were masked to treatment assignment. Double-blind, double-dummy.
Blinding of outcome assessment (detection bias)	Low risk	Patients and investigators were masked to treatment assignment. Double-blind, double-dummy
Incomplete outcome data (attrition bias)	Low risk	1300/1549 completed, 1549 included in full data analysis set. All drops outs and losses to follow up accounted for.
Selective reporting (reporting bias)	Low risk	All clinically relevant outcomes are defined and reported. Lipid profile was specified in the study protocol, but not reported in the main study report (present in the appendix).

Other bias	Unclear risk	This study was sponsored by Boehringer Ingelheim and Eli Lilly. Medical writing assistance, supported financially by Boehringer Ingelheim. Some of the trialists are employees of Boehringer Ingelheim.

Roden 2013

**Methods**	24 week, double-blind, parallel-group, randomized at 124 in 9 countries
**Participants**	899 initially randomized: empagliflozin 10 mg n=224; empagliflozin 25 mg n=224; sitagliptin n=22
**Interventions**	Randomly allocated patients (1:1:1:1) to empagliflozin 10 mg, 25 mg, sitagliptin 100 mg or placebo (oral dose) for 24 weeks.<br>Rescue medication permitted in case of hyperglycemia.<br>Add on: none
**Outcomes**	**Primary outcomes of the trial**<br>change from baseline in HbA1c at week 24.<br>**Secondary outcomes of the trial**<br>change from baseline in bodyweight at week 24 and<br>change from baseline in systolic and diastolic blood pressures at week 24.<br>percentage of patients with HbA1c of at least 7·0% at baseline who had HbA1c lower than 7·0% at week 24,<br>change from baseline in fasting plasma glucose at week 24,<br>percentage of patients with a greater than 5·0% reduction in bodyweight at week 24,<br>change from baseline in waist circumference at week 24,<br>percentage of patients who had uncontrolled blood pressure at baseline who had controlled blood pressure (systolic blood pressure <130 mm Hg and diastolic blood pressure <80 mm Hg) at week 24.<br>change from baseline in HbA1c at week 24 in subgroups of patients with baseline HbA1c of at least 8·5% and lower than 8·5%.<br>use of rescue therapy<br>**Adverse events and safety assessments**<br>vital signs,<br>clinical laboratory parameters,

	confirmed hypoglycemic adverse events<br>urinary tract infection<br>genital infection
**Inclusion criteria**	men and women<br>with T2DM<br>>18 years (≥20 years in Japan or 18–65 years in India)<br>BMI ≥45 kg/m^2,<br>insufficient glycemic control despite a diet and exercise regimen<br>HbA1c 7·0–10·0% (or HbA1c 7·0–9·0% in Germany) at screening.
**Exclusion criteria**	uncontrolled hyperglycemia (glucose concentration >13·3 mmol/L after an overnight fast during the placebo run-in phase and confirmed by a second measurement),<br>eGFR, estimated with the modification of diet in renal disease (MDRD) equation) of <50 mL/min per 1·73 m² (or <60 mL/min per 1·73 m² in China),<br>contraindications to sitagliptin according to the local label,<br>treatment with antiobesity drugs within 3 months before informed consent,<br>treatment with systemic steroids<br>change in dose of thyroid hormones within 6 weeks before informed consent,<br>uncontrolled endocrine disorder apart from type 2 diabetes.
**Notes**	ClinicalTrials.gov identifier: NCT01177813.<br>52-week non-randomized extension (NCT01289990) and open arm with poor glycemic control (HbA1c >10 %), not included in meta-analysis.

Risk of bias table

**Bias**	**Authors' judgement**	**Support for judgement**
Random sequence generation (selection bias)	Low risk	Patients with HbA1c 7-10 randomly allocated in a triple-dummy manner (1:1:1:1 ratio) to oral in block sizes of four and stratified by region (Asia, Europe, and North America), HbA1c at screening ≥8·5%) and eGFR at screening (≥90 mL/min per 1·73 m²,<br>60–89 mL/min per 1·73 m², and 50–59 mL/min per 1·73 m²).
Allocation	Low risk	Interactive voice and Internet-based response system,

concealment (selection bias)		with a computer generated random sequence, with no access by the investigators.<br><br>"Access to the randomization code was strictly limited to non-trial team functions including a randomization operator, a trained person to generate the randomization scheme, clinical trial supply unit staff responsible for packaging and labelling, an independent randomization statistician to verify and release the randomization scheme, a system operator for clinical data systems to perform the technical aspects of uploading the randomization scheme, a dedicated contract research organization (CRO) responsible for the interactive voice and internet-based response system, and a dedicated CRO supporting the Data Monitoring Committee. Investigators did not have access to the randomization code however, a code break was available to the investigator" via the interactive voice and internet-based response system in emergencies.
Blinding of participants and personnel (performance bias)	Low risk	Participants, investigators, and individuals involved in analysis of trial data were masked to treatment assignment during the randomized treatment period. Medications administered in a triple-dummy manner.
Blinding of outcome assessment (detection bias)	Low risk	Investigators, and individuals involved in analysis of trial data were masked to treatment assignment during the randomized treatment period.
Incomplete outcome data (attrition bias)	Low risk	803/889 completed. All 880 randomized were included in the full analysis set. Drop outs and losses to follow up accounted for.
Selective reporting (reporting bias)	Low risk	All clinically relevant outcomes are defined and reported.
Other bias	Unclear risk	Boehringer Ingelheim as study sponsor was involved in study design, data collection, and data analysis. Eli Lilly co-sponsored the study but was not involved in study design, data collection, or data analysis. Medical writing assistance, supported financially by Boehringer Ingelheim. Some of the trialists are employees of Boehringer Ingelheim.

Rosenstock 2012

**Methods**	12 week randomized, double-blind, placebo-controlled, parallel-group, multicenter, dose-ranging study in 85 study sites in 12 countries.
**Participants**	451 initially randomized: canagliflozin 50mg n=64; canagliflozin 100mg n=64; canagliflozin 300mg n=64; canagliflozin sitagliflozin n=65; placebo n= 65.
**Interventions**	Seven treatment groups: canagliflozin at doses of 50, 100, 200, or 300 mg once daily (QD) or 300 mg twice daily (BID); sitagliptin 100 mg QD, versus placebo for 12 weeks<br><br>Add on: metformin
**Outcomes**	**Primary outcomes of the trial**<br><br>change in A1C from baseline to week 12.<br><br>**Secondary outcomes of the trial**<br><br>fasting plasma glucose (FPG),<br>overnight urinary glucose-to-creatinine (UGlucose-to-UCreatinine) ratio,<br>body weight,<br>change in the percentage of participants with A1C ≥7.0% and ≥6.5%<br>change in fasting serum lipids (triglycerides, HDL cholesterol, LDL cholesterol, total cholesterol, and total cholesterol– to–HDL cholesterol ratio), β-Cell function was indirectly assessed by changes in homeostasis model assessment 2 (HOMA2) index of β-cell function (HOMA2-%B)<br><br>**Adverse events and safety assessments**<br><br>adverse event (AE) reports,<br>vital signs,<br>12-lead electrocardiograms,<br>physical examinations,<br>laboratory assessments.<br>vaginal swabs for Candida culture and<br>urine cultures were to be obtained from all at baseline and week 12 and at the time of a vulvovaginal AE<br>hypoglycemia.
**Inclusion criteria**	men and women

	aged 18–65 years (mean 52.9 years, SD 8.1))<br>T2DM for at least 3 months (mean duration 6.0 SD 4.9)<br>A1C level ≥7% and ≥10.5%,<br>on metformin monotherapy at a stable (>3 months) dose of >1,500 mg/day,<br>stable body weight and BMI 25–45 kg/m^2 (24–45 kg/m^2 for those of Asian descent),<br>serum creatinine levels <1.5 mg/dL for men and <1.4 mg/dL for women.
**Exclusion criteria**	None described.
**Notes**	ClinicalTrials.gov identifier: NCT00642278

Risk of bias table

Bias	Authors' judgement	Support for judgement
Random sequence generation (selection bias)	Unclear risk	Not described.
Allocation concealment (selection bias)	Unclear risk	Not described.
Blinding of participants and personnel (performance bias)	Low risk	Double blind.
Blinding of outcome assessment (detection bias)	Low risk	Double blind.
Incomplete outcome data (attrition bias)	Low risk	402/451 initially randomized completed the 12 week intervention.<br>Efficacy analyses were based on the intent-to-treat analysis set (all randomized participants), and safety analyses included all intent-to-treat participants who received at least one dose of the study medication.<br>Discontinuation because of AEs was documented (11 overall, 9 of whom were in the canagliflozin groups) and

		the numbers of drops out and losses to follow up and the reasons for this were described.
Selective reporting (reporting bias)	Low risk	All major clinically relevant outcomes are reported.<br><br>The reporting of urogenital infections is divided into many different terms (i.e. VVAEs in female participants, Urinary tract AEs, Symptomatic genital infections, Urinary tract infection, vulvovaginal mycotic infection). It is unclear how these items overlap.
Other bias	Unclear risk	This study was funded by Janssen Global Services, LLC. Editorial assistance was supported by Janssen Global Services, LLC.

Rosenstock 2012a

**Methods**	24-week, randomized, double-blind, placebo-controlled, parallel group, 105 sites in Argentina, Canada, India, Mexico, Peru, Philippines, Taiwan, and United States
**Participants**	420 initially randomized dapagliflozin 5mg (n = 141) or 10 mg (n = 140) or placebo (n = 139)
**Interventions**	Randomized patients received double-blind dapagliflozin 5 or 10 mg or placebo every day (oral administration) with open-label pioglitazone 30 or 45 mg/day, stratified by pre-enrollment diabetes treatment<br>group A and B.<br><br>Add on: pioglitazone<br><br>Rescue medication permitted
**Outcomes**	**Primary outcomes of the trial**<br><br>change at 24 weeks from baseline in HbA1c<br><br>**Secondary outcomes of the trial**<br><br>change from baseline in FPG,<br>postprandial glucose (PPG) measured by 120-min post-challenge response to an oral glucose tolerance test<br>total body weight.<br><br>**Adverse events and safety assessments**

	adverse events,<br>laboratory abnormalities,<br>vital signs<br>hypoglycemia<br>urinary tract infection (UTI) and<br>genital infection<br>seated blood pressure
**Inclusion criteria**	men and women<br>T2DM<br>≥18 years<br>fasting C-peptide ≥1.0 ng/mL<br>BMI ≤45.0 kg/m2 entered group A or B.<br>Group A patients had received ≥12 weeks of pioglitazone 30 or 45 mg/day and had HbA1c≥7.0 and ≤10.5%.<br>Group B patients were drug naive for the previous 10 weeks with HbA1c ≥8.0 and ≤ 11.0% or had received pioglitazone 15 mg/day or any dose of rosiglitazone with HbA1c ≥8.0 and ≤11.0%or had received ≥8 weeks of metformin 1700 mg/day or sulfonylurea less than or equal to half the maximal dose with HbA1c ≥7.0 and ≥11.0%.<br>Group B patients could not be on >1 oral antidiabetic medication, and patients on more than half the maximum dose of sulfonylurea or metformin were excluded.
**Exclusion criteria**	aspartate or alanine aminotransferases >2.5 times the upper limit of normal,<br>total bilirubin >2.0 mg/dL,<br>serum creatinine ≥2.0mg/dL,<br>urine albumin/creatinine ratio >1,800 mg/g,<br>calculated creatinine<br>clearance <50 mL/min,<br>congestive heart failure class III or IV
**Notes**	Study MB102030<br><br>Background treatment pioglitazone, open label rescue medication (metformin or SU) from week 24-48. Data from these patients excluded from efficacy data. 48 weeks safety data are included in our analyses.

Risk of bias table

Bias	Authors'	Support for judgement

	judgement	
Random sequence generation (selection bias)	Unclear risk	Not described in detail. Stated to be randomized
Allocation concealment (selection bias)	Unclear risk	Not described.
Blinding of participants and personnel (performance bias)	Low risk	Double blind placebo.
Blinding of outcome assessment (detection bias)	Low risk	Double blind placebo.
Incomplete outcome data (attrition bias)	Low risk	367/420 completed. All dropouts and losses to follow-up accounted for.
Selective reporting (reporting bias)	Unclear risk	All clinically relevant outcomes are defined and reported
Other bias	Unclear risk	Study supported by Bristol-Myers Squibb and AstraZeneca. Some of the trialists are Employees of Bristol Myers squib. Bristol-Myers Squibb provided editorial assistants

Rosenstock 2013

**Methods**	12-week randomized dose-ranging, double-blind, placebo-controlled trial at 104 centers in 16 countries
**Participants**	495 initially randomized: placebo n = 71, 1mg empagliflozin n = 71, 5mg empagliflozin n = 71,10 mg empagliflozin = 71,25 mg empagliflozin n=70,50 mg empagliflozin n=70, open-label sitagliptin n=70).
**Interventions**	Randomized to receive one of five doses of empagliflozin (1, 5, 10, 25 or 50 mg oral dose, once daily), or placebo,<br>Add on: metformin
**Outcomes**	**Primary outcomes of the trial**<br>change in HbA1c from baseline to week 12 with empagliflozin

	groups versus placebo.<br><br>**Secondary outcomes of the trial**<br><br>change in HbA1c over time,<br>change from baseline to week 12 in fasting plasma glucose (FPG), body weight<br>the proportion of participants achieving HbA1c ≤7% or lowering of ≥0.5% at week 12 (measured at screening and at visits 2−7).<br><br>**Adverse events and safety assessments**<br>At visits 2-7:<br><br>hematocrit;<br>serum sodium, potassium, chloride, magnesium, calcium, phosphate, bicarbonate,<br>uric acid; microalbumin and α1-microglobulin levels in urine,<br>plasma levels of high-density lipoprotein cholesterol (HDL-C), low-density lipoprotein cholesterol (LDL-C) and triglycerides),<br>vital signs (including systolic and diastolic blood pressure),<br>adverse events.
**Inclusion criteria**	men and women<br>aged ≥18 to <80 years<br>T2DM<br>body mass index (BMI) ≤40 kg/m2;<br>previous treatment with metformin alone or with metformin and one other oral antidiabetic agent (OAD)<br>unchanged antidiabetic therapy for ≥10 weeks prior to screening including stable metformin therapy (≥1500 mg/day or maximum tolerated dose);<br>hemoglobin A1c (HbA1c) of ≥6.5 to ≤9% for patients on metformin and one other OAD, discontinued at the start of the washout period;<br>HbA1c >7 to ≤10% for those on metformin monotherapy; and HbA1c>7 to 10% for all participants at start of placebo run-in period.
**Exclusion criteria**	history of myocardial infarction, stroke or transient ischemic attack within 6 months<br>impaired hepatic or renal function;<br>diseases of the central nervous system;<br>chronic or clinically relevant acute infections;<br>history of clinically relevant allergy/hypersensitivity;<br>treatment with thiazolidinediones,<br>glucagon-like peptide-1 (GLP-1) analogues or insulin within 3

	months.
**Notes**	ClinicalTrials.gov identifier: NCT00749190<br>Dosage 25 mg versus placebo, as add-on to metformin, included in our analyses. Open label Sitagliptin arm not included.

Risk of bias table

**Bias**	**Authors' judgement**	**Support for judgement**
Random sequence generation (selection bias)	Low risk	Random allocation, by an interactive voice response system.
Allocation concealment (selection bias)	Unclear risk	Not fully described, but implied in the randomization schedule generated by interactive voice response system.
Blinding of participants and personnel (performance bias)	Low risk	Double blind placebo.
Blinding of outcome assessment (detection bias)	Low risk	Double blind placebo
Incomplete outcome data (attrition bias)	Low risk	473/495 completed. Full analysis set based on a modified last observation carried forward. Drop outs and losses to follow up accounted for.
Selective reporting (reporting bias)	Low risk	All clinically relevant outcomes are defined and reported.
Other bias	Unclear risk	This study was funded by Boehringer Ingelheim. Medical writing assistance, supported financially by Boehringer Ingelheim.

Rosenstock 2014

**Methods**	52-week, randomized, double-blind, placebo-controlled, parallel-group study in 104 centers across 14 countries.

**Participants**	563 initially randomized: placebo n = 188, empagliflozin 10 mg n = 186, or empagliflozin 25 mg n = 189.
**Interventions**	Randomized (1:1:1) to receive once-daily empagliflozin 10 mg, empagliflozin 25 mg, or placebo.<br>Add on: MDI insulin, with or without metformin, for 52 weeks (oral dose). Diet and exercise counselling based on local recommendations. Rescue medication permitted in case of hyperglycemia.
**Outcomes**	**Primary outcomes of the trial**<br>change from baseline in HbA1c at week 18.<br>**Secondary outcomes of the trial**<br>changes from baseline at week 52 in insulin daily dose, body weight, and HbA1c.<br>body weight at week 18;<br>changes from baseline at weeks 18 and 52 in fasting plasma glucose (FPG),<br>systolic blood pressure (SBP), and diastolic blood pressure (DBP);<br>percentage of patients with HbA1c ≥7% (≥53 mmol/mol) at baseline who had HbA1c <7% at weeks 18 and 52;<br>use of rescue therapy.<br>**Adverse events and safety assessments**<br>vital signs,<br>clinical laboratory parameters,<br>adverse events (AEs) up to 7 days after the last dose of study drug<br>confirmed hypoglycemic AEs<br>with urinary tract<br>genital tract infections.
**Inclusion criteria**	obese adults (BMI ≥30 and ≤45 kg/m2)<br>T2DM<br>insufficient glycemic control (HbA1c ≥7.5 to ≤10% ($58 to #86 mmol/mol) at screening) despite diet and exercise counselling and treatment with MDI insulin (total daily dose >60 international units) alone or in combination with metformin<br>(immediate or extended release, ≥1,500 mg/day, maximum tolerated dose, or maximum dose according to the local label).<br>For ≥12 weeks prior to randomization, insulin dose was not to be changed by >10% and metformin dose was to be unchanged.

	Premixed insulins were not permitted.
**Exclusion criteria**	uncontrolled hyperglycemia (glucose level >13.3 mmol/L after an overnight fast;<br>acute coronary syndrome, stroke, or transient ischemic attack within 3 months prior to consent;<br>indication of liver disease;<br>impaired renal function during screening or run-in (eGFR using the modification of diet and renal disease equation <60 mL/min/1.73 m^2);<br>gastrointestinal surgeries that induce malabsorption;<br>cancer (except for basal cell carcinoma);<br>disorders causing hemolysis or unstable erythrocytes;<br>treatment with systemic steroids;<br>change in dosage of thyroid hormones within 6 weeks;<br>treatment with anti-obesity drugs;<br>alcohol or drug abuse;<br>investigational drug intake within 30 days of intake of study drug.
**Notes**	ClinicalTrials.gov identifier: NCT01306214

Risk of bias table

**Bias**	**Authors' judgement**	**Support for judgement**
Random sequence generation (selection bias)	Low risk	Randomization was undertaken using a third-party interactive voice- and web-response system and was stratified by HbA1c, eGFR, region (Europe, North America, Latin America), and background antidiabetes therapy (insulin alone, insulin plus metformin).
Allocation concealment (selection bias)	Unclear risk	Not fully described, but implied in the third-party interactive voice- and web-response system.
Blinding of participants and personnel (performance bias)	Low risk	Double-blind.
Blinding of outcome assessment	Low risk	Double-blind.

(detection bias)		
Incomplete outcome data (attrition bias)	Low risk	475/563 Completed the 52-week intervention period. The full analysis dataset (FAS) uses a LOCF method and is patients treated with study medication who had a baseline HbA1c measurement. The PPS-completers-52 set is patients in the FAS who were on treatment up to day 357 and did not have important protocol violations. The reasons for drops outs and losses to follow up are in the supplementary publication of this study.
Selective reporting (reporting bias)	Low risk	All clinically relevant outcomes are defined and reported.
Other bias	Unclear risk	This study was funded by Boehringer Ingelheim and Eli Lilly. Some of the trialists are employees of Boehringer Ingelheim.

Rosenstock 2015

**Methods**	78-week, randomized, double-blind, placebo-controlled, parallel-group study in 97 centers across 7 countries
**Participants**	494 initially randomized: placebo empagliflozin 10 mg (n=169), empagliflozin 25 mg (n=155) or placebo (n=170)
**Interventions**	Randomized (1:1:1) to receive once-daily empagliflozin 10 mg, empagliflozin 25 mg, or placebo.<br>Add on: insulin with or without metformin and sulfonyl ureas<br>Rescue mediation permitted in case of hyperglycemia.
**Outcomes**	**Primary outcomes of the trial**<br>change from baseline in HbA1c at week 18.<br>**Secondary outcomes of the trial**<br>changes from baseline to week 78 in basal insulin dose and HbA1c.<br>Changes from baseline to weeks 18 and 78:<br>FPG<br>bodyweight,<br>percentage with HbA1c≥7% (≥53mmol/mol) at baseline who had

	HbA1c<7% (<53mmol/mol) at weeks 18 and 78.<br>systolic (SBP)<br>diastolic blood pressure (DBP).<br><br>**Adverse events and safety assessments**<br><br>vital signs,<br>clinical laboratory measures<br>lipid variables<br>adverse events including included all events with an onset after the first dose and up to 7 days after the last dose of study medication,<br>confirmed hypoglycemic AEs (plasma glucose ≤3.9mmol/l (≤70mg/dl) and/or requiring assistance),<br>suspected and confirmed urinary tract infections (i.e. confirmed infections were those that led to hospitalization or discontinuations of the study drug),<br>genital infections.
**Inclusion criteria**	adults with T2DM<br>BMI ≤45 kg/m^2,<br>inadequately controlled type 2 diabetes (HbA1c >7 to ≤10% (>53 to ≤86mmol/mol) at screening), despite treatment with basal glargine or detemir insulin (≥20 IU/day) or NPH insulin (≥14 IU/day; at a dose unchanged by >10% of baseline value for ≥12 weeks before randomization), with or without metformin and/or sulphonylurea use (unchanged for ≥12weeks prior to randomization).
**Exclusion criteria**	uncontrolled hyperglycemia [glucose level >13.3mmol/l (>240mg/dl) after an overnight fast or >22.2mmol/l (>400mg/dl) from a random assessment during placebo run-in),<br>frequent hypoglycemic events on basal insulin therapy (in the opinion of the investigator),<br>myocardial infarction,<br>stroke or transient ischemic attack <3months before consent,<br>eGFR <30 ml/min/1.73m^2,<br>bariatric surgery,<br>investigational drug intake within 2 months of consent,<br>treatment with anti-obesity drugs,<br>any oral anti diabetes medication (other than metformin or sulphonylurea),<br>chronic short-acting insulin or glucagon-like peptide-1 receptor agonists within 3 months of consent,<br>impaired hepatic function (serum alanine aminotransferase, aspartate aminotransferase, or alkaline phosphatase >3 times the upper limit of normal),<br>history of cancer (except for basal cell and squamous cell skin

	cancer) within 5 years,<br>contraindication to background anti-diabetes medication according to the local label,<br>disorders causing hemolysis or unstable red blood cells; treatment with systemic steroids at time of consent,<br>change in dosage of thyroid hormones within 6 weeks prior to consent,<br>alcohol or drug abuse.
**Notes**	ClinicalTrials.gov identifier: NCT01011868

Risk of bias table

Bias	Authors' judgement	Support for judgement
Random sequence generation (selection bias)	Low risk	Randomization was performed using a third-party interactive voice and web response system, and was stratified by HbA1c at screening (<8.5% (<69mmol/mol ≥8.5% (≥69mmol/mol)) and center.
Allocation concealment (selection bias)	Unclear risk	Not fully described, but implied in the third-party interactive voice- and web-response system.
Blinding of participants and personnel (performance bias)	Low risk	Stated to be double blind, placebo controlled although no details given.
Blinding of outcome assessment (detection bias)	Low risk	Stated to be double blind, placebo controlled although no details given.
Incomplete outcome data (attrition bias)	Low risk	429/494 completed 18 weeks treatment, 360/494 completed 78 weeks. The full analysis set comprised randomized patients treated with ≥1 dose of study drug and who had a baseline HbA1c value) at week 18.
Selective reporting (reporting bias)	Low risk	All clinically relevant outcomes are defined and reported.
Other bias	Unclear risk	This study was funded by Boehringer Ingelheim and Eli Lilly. Some of the trialists are employees of Boehringer Ingelheim.

Rosenstock 2015a

**Methods**	24-week, multicenter, randomized, double-blind, active-controlled, parallel- group phase 3 study in eight countries (Canada, Mexico, Poland, Puerto Rico, Republic of Korea, Romania, South Africa, United States)
**Participants**	534 initially randomized: 179 saxagliptin + dapagliflozin + metformin; 176 saxagliptin + metformin; 179 dapagliflozin + metformin
**Interventions**	Randomized 1:1:1 using a centralized blocked randomization schedule to receive saxagliptin (5 mg/day) and dapagliflozin (10 mg/day) plus MET (SAXA+Dapagliflozin 10mg+MET), saxagliptin (5 mg/day) and placebo plus MET<br>(SAXA+MET), or dapagliflozin (10mg/day) and placebo plusMET (Dapagliflozin 10mg+MET) for 24 weeks. Other anti-diabetic medications prohibited during the screening and treatment periods.<br>Add on: metformin<br>Rescue medication open-label rescue medication, including insulin or other antidiabetic medications, except metformin, GLP-1 receptor agonists, and other DPP-4 inhibitors or SGLT2 inhibitors, was given.
**Outcomes**	**Primary outcomes of the trial**<br>adjusted mean change from baseline in HbA1c after 24 weeks of double-blind treatment.<br>**Secondary outcomes of the trial**<br>adjusted mean change from baseline at 24 weeks in 2-h postprandial glucose (PPG),<br>adjusted mean change from baseline at 24 weeks in FPG,<br>adjusted mean proportion of patients achieving a therapeutic glycemic response, defined as HbA1c >7.0% (53 mmol/mol), after 24 weeks,<br>adjusted mean change from baseline in body weight.<br>PPG after the administration of a liquid meal<br>adjusted mean changes from baseline at 24 weeks in fasting serum lipids.<br>**Adverse events and safety assessments**<br>adverse events (AEs),<br>hypoglycemia,<br>laboratory abnormalities,

	vital signs.<br>severe cutaneous events,<br>decreased lymphocyte count,<br>decreased thrombocyte count,<br>opportunistic infection,<br>pancreatitis,<br>hepatic AEs,<br>fracture,<br>hypersensitivity,<br>worsening renal function,<br>genital infections,<br>urinary tract infections,<br>bladder neoplasm,<br>breast neoplasm<br>blood pressure (investigators adjusted antihypertensive therapy as needed).
**Inclusion criteria**	≥18 years, mean age of 54<br>T2DM<br>inadequate glycemic control, defined as HbA1c ≥8.0% and ≤12.0% (64–108 mmol/mol)<br>on stable metformin therapy (≥1,500 mg/day) for ≥8 weeks<br>C peptide concentrations ≥1.0 ng/mL<br>BMI ≤45.0 kg/m^2 at screening.
**Exclusion criteria**	pregnancy<br>uncontrolled hypertension (systolic blood pressure ≥160mmHg and diastolic blood pressure ≥100 mmHg) at randomization,<br>fasting plasma glucose (FPG) ≥270 mg/dL during the 4-week lead-in period,<br>cardiovascular disease within 3 months of screening,<br>congestive heart failure (New York Heart Association functional class IV),<br>eGFR 60 mL/min/1.73m2 or<br>serum creatinine ≥1.5mg/dL in men or ≥1.4mg/dL in women,<br>significant hepatic disease<br>any antidiabetic medication, other than metformin, for more than 14 days during the 12 weeks before screening.
**Notes**	ClinicalTrials.gov identifier: NCT01606007<br><br>Patient demographics and baseline characteristics were balanced across treatment groups

Risk of bias table

Bias	Authors' judgement	Support for judgement
Random sequence generation (selection bias)	Low risk	Randomized 1:1:1 using a centralized blocked randomization schedule
Allocation concealment (selection bias)	Unclear risk	Not described but implied by computerized randomization.
Blinding of participants and personnel (performance bias)	Low risk	Double blind placebo.
Blinding of outcome assessment (detection bias)	Low risk	Double blind placebo.
Incomplete outcome data (attrition bias)	Low risk	534/590 completed. All dropouts and lost to follow-up accounted for. The primary efficacy data set included all randomized patients who received at least one dose of a study medication during the double-blind treatment period.
Selective reporting (reporting bias)	Low risk	All clinical relevant outcomes are defined and reported.
Other bias	Unclear risk	This study was funded by Bristol-Myers Squibb and AstraZeneca. Medical writing support funded from Bristol Myers squib and AstraZeneca.

Ross 2015

**Methods**	16-week, randomized, double-blind, placebo-controlled, parallel-group study in multiple centers across Europe, North America, Latin America)
**Participants**	983 initially randomized: empagliflozin 12.5mg twice daily (n=219), 25mg once daily (n=218), 5 mg twice daily (n=219) or 10 mg once daily (n=220), or placebo (n=107).
**Interventions**	Randomized 2 : 2 : 2 : 2 : 1 to receive empagliflozin 12.5mg twice daily, 25mg once daily, 5mg twice daily or 10mg once daily, or

	placebo, for 16 weeks<br>Add on: diet and exercise counselling based on local recommendations.<br>Rescue mediation permitted in case of hyperglycemia (>13.3mmol/l).
**Outcomes**	**Primary outcomes of the trial**<br>change from baseline in HbA1c at week 16.<br>**Secondary outcomes of the trial**<br>change from baseline in fasting plasma glucose (FPG) at week 16,<br>proportion of patients with HbA1c ≥7% at baseline who had HbA1c <7% at week 16,<br>changes from baseline at week 16,<br>weight,<br>systolic blood pressure (SBP),<br>diastolic blood pressure (DBP).<br>**Adverse events and safety assessments**<br>confirmed hypoglycemic AEs (defined as AEs with plasma glucose ≤3.9mmol/l and/or requiring assistance).<br>urinary tract infection (UTI),<br>genital infection,<br>increased urination,<br>volume depletion.
**Inclusion criteria**	adults with T2DM<br>BMI ≤45 kg/m^2,<br>glycated hemoglobin (HbA1c) level of ≥7 and ≤10% at screening (despite a diet and exercise regimen),<br>treatment with a stable dose of metformin IR ≥1500mg/day) for ≥12 weeks before randomization.
**Exclusion criteria**	uncontrolled hyperglycemia (plasma glucose >13.3 mmol/l after an overnight fast during a 2-week placebo run-in period, confirmed by a second measurement),<br>renal impairment (estimated creatinine clearance rate <60 ml/min using the Cockcroft-Gault formula),<br>indication of liver disease (serum alanine aminotransferase, aspartate aminotransferase, or alkaline phosphatase >3 x upper limit of normal) at screening or during the placebo run-in,<br>acute coronary syndrome,

	stroke,<br>transient ischemic attack within 3 months prior to consent,<br>had received anti-obesity drugs within 3 months of consent, had undergone bariatric surgery within 2 years,<br>any uncontrolled endocrine disorder except T2DM,<br>had received any antidiabetes agent other than metformin IR in the 12 weeks prior to consent.
**Notes**	EudraCT number 2012-000905-53<br><br>NB: we compared the 25mg arm with placebo

Risk of bias table

**Bias**	**Authors' judgement**	**Support for judgement**
Random sequence generation (selection bias)	Low risk	Implied by the declaration that 'randomization was stratified by region (Europe, North America, Latin America) and by HbA1c (<8.5 and ≥8.5%) and estimated glomerular filtration rate (≤60–89 ml/min/1.73m2 and ≥90 ml/min/1.73m2) at screening'.
Allocation concealment (selection bias)	Unclear risk	No details given about allocation concealment.
Blinding of participants and personnel (performance bias)	Low risk	Stated to be double blind, but no details given.
Blinding of outcome assessment (detection bias)	Low risk	Stated to be double blind, but no details given.
Incomplete outcome data (attrition bias)	Low risk	916/983 completed 16-week treatment period. Al drop outs and losses to follow up accounted for. The full analysis dataset uses a LOCF method and is patients treated with study medication ≥1 dose of study drug and had a baseline and on-treatment HbA1c value).
Selective reporting (reporting bias)	Low risk	All clinically relevant outcomes are defined and reported.

Other bias	Unclear risk	Study funded by Boehringer Ingelheim and Eli Lilly and Company. Medical writing assistance, supported financially by Boehringer Ingelheim. Some of the study authors are employees of Boehringer Ingelheim.

Schernthaner 2013

**Methods**	52 week, randomized, double-blind, active- controlled, multicenter study phase 3 study at 140 centers in 17 countries
**Participants**	755 initially randomized: canagliflozin 300mg n=378; sitagliflozin n= 378)
**Interventions**	Canagliflozin 300 mg versus sitagliptin 100 mg once daily (1:1) (oral dose) for 52 weeks.<br><br>Add on: metformin
**Outcomes**	**Primary outcomes of the trial**<br><br>Change in A1C from baseline to week 52.<br><br>**Secondary outcomes of the trial**<br><br>change from baseline in FPG,<br>systolic blood pressure (BP),<br>percent change from baseline in body weight,<br>triglycerides, and HDL cholesterol (HDL-C).<br>proportion of subjects reaching A1C >7.0% (53 mmol/mol) and >6.5% (48 mmol/mol),<br>change in diastolic BP,<br>percent change in other fasting plasma lipids.<br>Indices of β-cell function (βCF) (HOMA2-%B),<br>proinsulin/insulin ratio, and proinsulin/ C-peptide ratio,<br>a subset underwent a FS-MMTT and postprandial glucose measurements.<br><br>**Adverse events and safety assessments**<br><br>adverse events (AEs),<br>clinical laboratory tests,<br>vital sign measurements,<br>physical examinations,<br>self-monitored blood glucose,<br>12-lead electrocardiograms,<br>hypoglycemic episodes,

	genital mycotic infections and urinary tract infections (UTIs),<br>incidence of hypoglycemia.
**Inclusion criteria**	men and women 18 years of age or older<br>T2DM<br>using stable metformin and sulfonylurea therapy.<br>(metformin ≥2,000 mg/day (or ≥1,500 mg/day if unable to tolerate a higher dose); sulfonylurea at half-maximal labelled dose or more),<br>A1C ≥7.0% (53 mmol/mol) and ≤10.5% (91 mmol/mol),<br>met all other enrolment criteria
**Exclusion criteria**	(FPG) or fasting self-monitored blood glucose measurements ≥16.7 mmol/L (300mg/dL), or both, during the pre-treatment phase;<br>history of type 1 diabetes,<br>cardiovascular disease,<br>uncontrolled hypertension,<br>treatment with either a PPARγ agonist, ongoing insulin therapy, another SGLT2 inhibitor, or any other AHA (other than metformin and a sulfonylurea) within 12 weeks before screening,<br>eGFR 55 mL/min/1.73 m^2 (60 mL/min/1.73 m^2 if based on restriction of metformin use in the metformin local label),<br>serum creatinine ≥124 mmol/L (men) and ≥115 mmol/L (women).
**Notes**	ClinicalTrials.gov identifier: NCT01137812<br><br>The study consisted of a 52-week double-blind treatment phase, and a 4-week follow-up period data from which are included in our analyses.

Risk of bias table

**Bias**	**Authors' judgement**	**Support for judgement**
Random sequence generation (selection bias)	Low risk	Interactive Voice Response System/ Interactive Web Response System. The computer-generated randomization schedule was prepared by the sponsor before the study, and randomization was balanced using permuted blocks with the following two stratification criteria: whether the pre randomization A1C was ≥9.0% (75 mmol/mol) and whether a subject underwent the frequently sampled mixed-meal tolerance test (FS-MMTT).

Allocation concealment (selection bias)	Unclear risk	Not described, but implied in interactive voice response system/ interactive web response system. Computer-generated randomization schedule.
Blinding of participants and personnel (performance bias)	Low risk	Participants, investigators, and local sponsor personnel remained blinded to treatment assignment and urine samples for glucosuria until all participants completed the study (week 52 visit) and the final database was locked.
Blinding of outcome assessment (detection bias)	Low risk	After randomization, A1C and FPG values and all glucose levels from the FS- MMTT were masked to the study centers, unless FPG/A1C values met specific study criteria for discontinuation.<br><br>Participants, investigators, and local sponsor personnel remained blinded to treatment assignment and urine samples for glucosuria until all participants completed the study (week 52 visit) and the final database was locked.
Incomplete outcome data (attrition bias)	Low risk	464/756 completed the intervention. The primary analysis was based on the modified intent- to-treat population (all randomized participants who received one or more doses of study drug) with a last observation carried forward approach to impute missing data at the end point.<br><br>A higher rate of discontinuation was observed with sitagliptin 100 mg (44.4%) than with canagliflozin 300 mg (32.6%).
Selective reporting (reporting bias)	Low risk	All major clinically relevant outcomes were reported
Other bias	Unclear risk	This study was supported by Janssen Research & Development LLC, who funded editorial support.

Stenløf 2013

**Methods**	26-week, randomized, double-blind, placebo-controlled, phase 3 trial in 17 countries.
**Participants**	587 initially randomized: canagliflozin 100mg n=195; canagliflozin 300mg n=197; placebo n=192.
**Interventions**	Participants in the main study were randomly assigned to receive daily oral doses of canagliflozin 100 or 300mg versus placebo (1 : 1 :

	1) once daily (oral dose) for 26 weeks.<br><br>Add on: diet and exercise continued during the intervention period.
**Outcomes**	**Primary outcomes of the trial**<br><br>change in HbA1c from baseline to week 26.<br><br>**Secondary outcomes of the trial**<br><br>proportion of participants reaching HbA1c <7.0%,<br>changes from baseline at week 26 in FPG and systolic blood pressure (BP),<br>percent changes from baseline in body weight,<br>high-density lipoprotein cholesterol (HDL-C) and triglycerides,<br>other fasting plasma lipids, including low-density lipoprotein cholesterol (LDL-C), non – HDL-C and the LDL-C/HDL-C ratio,<br>change from baseline in apolipoprotein B (Apo B) in a subset of participants in the main study (based on availability of paired baseline and week 26 archive samples<br>changes in diastolic BP.<br><br>**Adverse events and safety assessments**<br><br>adverse event (AE) reports,<br>safety laboratory tests,<br>vital sign measurements,<br>physical examinations and 12-lead electrocardiograms,<br>urinary tract infections (UTIs) and genital mycotic infections,<br>documented hypoglycemia episodes,<br>severe hypoglycemia episodes.
**Inclusion criteria**	men and women 18 to 80 years of age<br>T2DM<br>not on an AHA at screening<br>HbA1c ≥7.0 and ≤10.0%,<br>on AHA monotherapy (except peroxisome proliferator-activated receptor-γ (PPARγ) agonist) or metformin plus sulfonylurea combination therapy (at ≤50% of maximally or near-maximally effective doses) with HbA1c ≥6.5 and ≤9.5% at screening and HbA1c ≥7.0 and ≤10.0% and fasting plasma glucose (FPG) <15.0 mmol/l at week −2.
**Exclusion criteria**	repeated FPG measurements >15.0mmol/l during the pre-treatment phase (or >19.4 mmol/l for the high glycemic sub study),<br>type 1 diabetes,<br>hereditary glucose-galactose malabsorption,

	primary renal glucosuria,<br>cardiovascular (CV) disease (including myocardial infarction, unstable angina, revascularization procedure or cerebrovascular accident),<br>treatment with a PPARγ agonist, insulin, another SGLT2 inhibitor or any other AHA except as specified in the inclusion criteria within12 weeks before screening,<br>eGFR <50 ml/min/1.73m^2 at screening.
**Notes**	ClinicalTrials.gov identifier: NCT01081834<br><br>In this review, only the main placebo controlled study contributed data to our analyses, i.e. the study continued for 26 weeks (single blind) after initial intervention period, this review reports only outcomes from the initial 26-week double blind intervention. (An active-controlled, 26-week extension (blinded switch of placebo-treated patients to sitagliptin 100 mg (placebo/sitagliptin)) (CANA Stenløf 2014).<br><br>"For subjects with HbA1c values above the inclusion range (HbA1c ≥7.0 and ≤10.0%), a sub study was conducted to assess the efficacy in elevated glycemic states. Subjects were eligible to participate in the high glycemic sub study if they had HbA1c >10.0 and <12.0% at screening or week −1 and FPG >19.4mmol/l at week −1. Subjects eligible for this sub study entered a 1-week, single-blind, placebo run-in period followed by a 26-week, double-blind, active-treatment period. Given the poorer glycemic control, all subjects received active treatment with canagliflozin 100 or 300 mg; double-blinding was to the dose of canagliflozin. Subjects in the high glycemic sub study were not eligible for the 26-week extension period. In this report, the placebo-controlled study component will be referred to as the ‘main study’."

Risk of bias table

Bias	Authors' judgement	Support for judgement
Random sequence generation (selection bias)	Unclear risk	Not described
Allocation concealment	Unclear risk	Not described

(selection bias)		
Blinding of participants and personnel (performance bias)	Low risk	Described as double-blind
Blinding of outcome assessment (detection bias)	Low risk	Described as double-blind
Incomplete outcome data (attrition bias)	Low risk	584/587 completed the intervention i.e. they received at least one dose of the study medication.<br>Efficacy and safety analyses for the main study and the high glycemic sub study were performed separately using the modified intent-to-treat (mITT) population consisting of all randomized participants who received ≥1 dose of the study drug. The last observation carried forward (LOCF) approach was used to impute missing efficacy data. For participants who received rescue therapy, the last post-baseline value prior to the initiation of rescue therapy was used for the efficacy analyses.<br>All drops outs and losses to follow up accounted for. Slightly higher rates of discontinuation were seen with placebo.
Selective reporting (reporting bias)	Low risk	Some additional outcomes, besides the pre-specified ones were reported. But all major clinically relevant pre-specified outcomes reported.
Other bias	Unclear risk	This study was sponsored by Janssen Research & Development, LLC who provided assistance and contribution to the clinical management, data review and preparation of the study report.

Strojek 2011

**Methods**	24-week randomized, double-blind, parallel-group, placebo-controlled, multicenter trial at 84 sites in the Czech Republic (11 centers), Hungary (16), Republic of Korea (12), Philippines (5), Poland (29), Thailand (3) and Ukraine (8)

**Participants**	597 randomized to study drug + glimepiride 4 mg/day. 146 placebo; 154 dapagliflozin 2.5 mg/day; 145 dapagliflozin 5 mg/day; 151 dapagliflozin 10 mg/day.
**Interventions**	Randomized to receive double- blind dapagliflozin 2.5, 5 or 10 mg or placebo taken orally once per day before the first meal of the day and added to continuing open-label glimepiride 4 mg/day.<br><br>Add on: glimepiride and rescue therapy were administered as open label treatments for 24 weeks with a 24-weeks double blind extension period.
**Outcomes**	**Primary outcomes of the trial**<br><br>The primary endpoint was change in central laboratory HbA1c percentage from baseline to week 24,<br><br>**Secondary outcomes of the trial**<br><br>change in total body weight (TBW) from baseline to week 24<br>change from baseline to week 24 in 2-h post-challenge plasma glucose (PPG) rise in response to an oral glucose-tolerance test (OGTT) using 75 g of glucose<br>proportion of patients achieving a therapeutic<br>glycemic response, defined as HbA1c < 7% at week 24<br>change in TBW from baseline to week 24 in patients<br>with baseline BMI ≥27 kg/m2<br>change in FPG from<br>baseline to week 24.<br>proportions of patients receiving rescue therapy for failing to reach prespecified glycemic targets or discontinuing for lack of efficacy,<br>seated systolic and diastolic blood pressure<br>lipids<br><br>**Adverse events and safety assessments**<br><br>adverse events<br>hypoglycemic events,<br>laboratory tests,<br>electrocardiographic and physical examinations<br>vital signs (including orthostatic hypotension).<br>urinary tract infection (UTI)<br>genital infection
**Inclusion criteria**	men and women<br>aged ≥18 years

	inadequately controlled T2DM,<br>HbA1c ≥7 and ≤10%, who were receiving a stable dose of sulphonylurea monotherapy that was at a dose level of at least half the maximum recommended for at least 8 weeks prior to enrolment.<br>fasting plasma<br>glucose (FPG) >15 mmol/l and fasting C-peptide ≥0.33 nmol/l.
**Exclusion criteria**	type 1 diabetes,<br>diabetes insipidus,<br>corticosteroid-induced type 2 diabetes,<br>a history of diabetic ketoacidosis or hyperosmolar non-ketotic coma,<br>poorly controlled diabetes characterized by polyuria/polydipsia with >10% weight loss,<br>use of insulin for >7 consecutive days during the 24 weeks prior to enrolment,<br>and use of glimepiride >4 mg/day during the 8 weeks up to and including enrolment,<br>body mass index (BMI) >45.0 kg/m^{2},<br>calculated creatinine clearance <50 mL/min or serum creatinine >177 µmol/L,<br>urine albumin/creatinine ratio >203.4 mg/mmol,<br>aspartate aminotransferase and/or alanine aminotransferase and/or creatine kinase ≥3 ×upper limit of normal range,<br>serum total bilirubin >34 µmol/L; Hb ≤10 g/dL for men and ≤9.5 g/dL for women,<br>systolic blood pressure ≥180 mmHg and/or diastolic blood pressure ≥110 mmHg,<br>cardiovascular event within 6 months of enrolment,<br>congenital renal glycosuria,<br>significant renal, hepatic, hematological, oncological, endocrine, immunological (including hypersensitivity to study medications),<br>psychiatric disease (including alcohol and substance misuse),<br>pregnancy or lactation,<br>use of systemic corticosteroids for >4 weeks within 3 months of enrolment,<br>weight loss medication within 30 days of enrolment.
**Notes**	ClinicalTrials.gov identifier: NCT00680745<br><br>Data from the 24-week double-blind extension period Strojek 2014 were included in our analyses.<br><br>Demographic and baseline characteristics were balanced across treatment groups, with 30.6% of patients recruited from the

	Asia/Pacific region

Risk of bias table

Bias	Authors' judgement	Support for judgement
Random sequence generation (selection bias)	Low risk	A computer-generated randomization schedule was provided by AstraZeneca using blocks to balance the treatment groups in a 1 : 1 : 1 : 1 ratio. Patients were randomized strictly sequentially at each center.
Allocation concealment (selection bias)	Unclear risk	Not described in detail but implied by computer-generated randomization schedule using block randomization.
Blinding of participants and personnel (performance bias)	Low risk	Blinding of dapagliflozin tablets was achieved by double-blind allocation and use of a double- dummy technique because the dapagliflozin 10 mg tablet size was slightly larger than that for the 2.5 and 5 mg doses.
Blinding of outcome assessment (detection bias)	Low risk	Double blind. double-blind allocation and use of a double dummy technique because the dapagliflozin 10 mg tablet size was slightly larger than that for the 2.5 and 5 mg doses.
Incomplete outcome data (attrition bias)	Low risk	546/597 completed. Two analysis sets were defined: the safety analysis set consisting of all patients who received ≥1 dose of study medication and the full analysis set consisting of all randomized patients who received ≥1 dose of study medication and who had a non-missing baseline and ≥1 post-baseline efficacy value for ≥1 efficacy variable. Primary, secondary and exploratory endpoints were analyzed with the full analysis set. Observations after initiation of rescue therapy were excluded, and these and other missing values were replaced using the LOCF method. The majority of randomized patients completed the study (91.5%), All dropouts and lost to follow-up accounted for.
Selective reporting (reporting bias)	Low risk	Besides the major prespecified outcomes, a number of exploratory endpoints were assessed, which included proportions of patients receiving rescue therapy for failing to reach prespecified glycemic targets or discontinuing for

		lack of efficacy, seated systolic and diastolic blood pressure and lipid parameters.
Other bias	Unclear risk	Medical writing and editorial was funded by AstraZeneca and Bristol-Myers Squibb. Some of the trialists are employees of AstraZeneca. Data analysis was conducted by a company which is contracted to support data analysis for AstraZeneca.

Wilding 2009

**Methods**	12-week randomized, double-blind, three arm parallel-group, placebo-controlled, 26-center trial (U.S. and Canada).
**Participants**	71 initially randomized: placebo n=23; 10mg dapagliflozin n= 24; 20 mg dapagliflozin n=24
**Interventions**	Randomly assigned 1:1:1 on day 1 to double-blind placebo, 10 mg dapagliflozin, or 20 mg dapagliflozin once daily, in addition to open-label therapy with 50% of their usual daily insulin dose and their OAD(s).<br>Add on: metformin, insulin, pioglitazone, rosiglitazon
**Outcomes**	**Primary outcomes of the trial**<br>change from baseline in A1C at week 12<br>**Secondary outcomes of the trial**<br>changes from baseline in FPG<br>total daily dose of insulin (TDDI),<br>proportion achieving a decrease in A1C 0.5% from baseline,<br>proportion patients achieving A1C 7%.<br>total body weight<br>postprandial glucose (PPG) measured by an oral glucose tolerance test.<br>**Adverse events and safety assessments**<br>emergent adverse events,<br>vital signs,<br>laboratory measurements, including 24-h urine collections for volume and electrolytes.
**Inclusion criteria**	men and women

	with type 2 diabetes,<br>aged 18–75 years<br>with BMI ≤45 kg/m^2<br>A1C 7.5–10%,<br>stable-dose insulin sensitizer therapy (metformin 1,000 mg and/or pioglitazone 30 mg or rosiglitazone 4 mg) for 6 weeks and insulin therapy for 12 weeks before enrolment<br>fasting C-peptide 0.8 ng/ml,<br>serum creatinine 1.5 mg/dl (men) or 1.4 mg/dl (women)<br>urine microalbumin-to creatinine ratio 300 mg/g<br>24-h urine total protein 3 g/24 h.
**Exclusion criteria**	type 1 diabetes,<br>aspartate transaminase and/or alanine transaminase2.5 times the upper limits of normal,<br>creatine kinase 3 times the upper limits of normal,<br>symptoms of severely uncontrolled diabetes,<br>history of severe hypoglycemia,<br>unstable condition or serious cardiovascular, renal, or hepatic disease.
**Notes**	No ClinicalTrials.gov identifier.<br>Receiving high doses of insulin and insulin sensitizers

Risk of bias table

**Bias**	**Authors' judgement**	**Support for judgement**
Random sequence generation (selection bias)	Unclear risk	Randomly assigned, but no details provided.
Allocation concealment (selection bias)	Unclear risk	No details provided.
Blinding of participants and personnel (performance bias)	Low risk	Double blind placebo.
Blinding of outcome assessment (detection	Low risk	Double blind placebo.

bias)		
Incomplete outcome data (attrition bias)	Low risk	60/71 completed. Last observation carried forward. The primary efficacy dataset consisted of all randomly assigned patients who took 1 dose of double-blind study medication. All dropouts and lost to follow-up accounted for.
Selective reporting (reporting bias)	Low risk	All clinically relevant outcomes are defined and reported.
Other bias	Unclear risk	This study was funded by Bristol-Myers Squibb and AstraZeneca. Bristol-Myers Squibb provided writing and editorial support.

Wilding 2012

**Methods**	48-week, randomized, placebo-controlled, parallel group, multicenter trial 126 centers worldwide (Austria, Bulgaria, Canada, Finland, Great Britain, hungry, the Netherlands, Romania, Russian Federation, Slovakia, Spain, United States).
**Participants**	808 initially randomized: placebo plus insulin n = 193; dapagliflozin 2.5 mg plus insulin N = 202; dapagliflozin 5 mg plus insulin n = 211; dapagliflozin 10 mg plus insulin n= 194
**Interventions**	Randomly assigned on a 1:1:1:1 basis to receive placebo or 2.5, 5, or 10 mg of dapagliflozin, once daily, for 24 weeks, extended to 48 weeks.<br><br>Add on: open-label therapy with their usual daily dose of insulin and existing oral antidiabetic drugs (OADs).<br><br>No rescue medication reported.
**Outcomes**	**Primary outcomes of the trial**<br><br>change in HBA1c from baseline to 48 weeks<br><br>**Secondary outcomes of the trial**<br><br>bodyweight,<br>insulin dose,<br>fasting plasma glucose level at 24 weeks<br><br>**Adverse events and safety assessments**

	adverse events,<br>laboratory variables,<br>vital signs,<br>hyperglycemia,<br>genital infection,<br>urinary tract infection (UTI)
**Inclusion criteria**	men and women<br>aged 18 to 80 years<br>T2DM<br>BMI ≤ 45 kg/m^2<br>inadequate glycemic control (HbA1c ≥7.5% and ≤10.5%).<br>Participants had to have received a stable insulin regimen with a mean daily insulin dose of 30 U or more for at least 8 weeks, with daily insulin requirements varying by more than 10% on no more than 1 occasion in the 7 days before randomization.
**Exclusion criteria**	Type I diabetes mellitus,<br>symptoms of poorly controlled diabetes,<br>calculated creatinine clearance less than 50 mL per minute per 1.73 m^2, or measured serum creatinine level greater than hundred and 77 µmol per liter (>2 mg/dL) or if receiving metformin greater than 133 µmol per liter (>1.5 micrograms per deciliter) for men and at least 124 µmol per liter (≥1.4 mcg/dL) for women
**Notes**	ClinicalTrials.gov identifier: NCT00673231<br><br>We included the 24-week extension study results (Wilding 2014) in our analyses (i.e. 48-week outcome data).

Risk of bias table

**Bias**	**Authors' judgement**	**Support for judgement**
Random sequence generation (selection bias)	Low risk	A computer-generated, stratified, block-randomization schedule containing stratum, randomization code, and treatment was provided by AstraZeneca.
Allocation concealment (selection bias)	Unclear risk	Not described in detail but implied in computer-generated randomization.
Blinding of	Low risk	Patients, investigators, study monitors, and personnel at

participants and personnel (performance bias)		AstraZeneca and Bristol-Myers Squibb had no access to the randomization scheme, except for cases of medical emergencies.
Blinding of outcome assessment (detection bias)	Unclear risk	Because primary efficacy analyses were planned at 24 weeks, personnel at AstraZeneca and Bristol- Myers Squibb had access to the data at that time. During the double-blind extension periods, investigators, patients, and study monitors remained blinded, except for cases of medical emergencies.
Incomplete outcome data (attrition bias)	Low risk	Two analysis sets were defined: the safety set, comprising all randomly assigned patients who received at least 1 dose of study medication, and the full set, comprising all randomly assigned patients who received at least 1 dose of study medication and had a non-missing baseline value and at least 1 post-baseline efficacy value for at least 1 efficacy variable. Efficacy variables were analyzed with the full analysis set. Completers PLA: 157/197, Dapagliflozin 10mg 170/196.
Selective reporting (reporting bias)	Low risk	All major clinically relevant outcomes have been reported.
Other bias	Unclear risk	Sponsored by AstraZeneca and Bristol Myers squib. Sponsors involved in the study design, data collection, review and analysis, editorial assistants funded by the sponsors.

Wilding 2013

**Methods**	78 week randomized, double-blind, placebo-controlled, multicenter study at 85 study centers in 11 countries followed by a 26-week, double-blind, extension period.
**Participants**	469 initially randomized: placebo n= 156; 100 mg canagliflozin n=157; canagliflozin n= 156
**Interventions**	1:1:1 ratio to receive canagliflozin 100 or 300 mg or placebo once daily (oral dose) before the first meal of the day for 26 weeks (extended to 52 weeks).<br>Add on: metformin, sulfonylurea
**Outcomes**	**Primary outcomes of the trial**

	change from baseline in HbA1c at week 52.<br><br>**Secondary outcomes of the trial**<br><br>proportion of patients achieving HbA1c <7.0%,<br>change from baseline in FPG and systolic BP,<br>per cent change from baseline in body weight,<br>high density lipoprotein cholesterol (HDL-C), and triglycerides.<br>Homeostasis Model Assessment (HOMA2-% B),<br>a fasting measure of β-cell function, based on FPG and C-peptide measurements,<br>in subset of patients: FS-MMTT on day 1 and at week 26, 2-h postprandial glucose (PPG), glucose area under the concentration-time curve (AUCG), incremental AUCG (#AUCG), and the ratio of C-peptide AUC (AUCC) to AUCG were assessed.<br><br>**Adverse events and safety assessments**<br><br>adverse event (AE) reports over the 52-week period,<br>safety laboratory tests,<br>vital sign measurements,<br>12-lead electrocardiograms,<br>physical examinations,<br>genital mycotic infections and urinary tract infections (UTIs),<br>hypoglycemia events.
**Inclusion criteria**	men and women<br>aged 18– 80 years<br>T2DM<br>inadequate glycemic control (HbA1c ≥7.0% to ≤10.5%),<br>on metformin plus sulphonylurea, with both agents at maximally or near-maximally effective doses.
**Exclusion criteria**	history of diabetic ketoacidosis,<br>T1DM<br>repeated fasting plasma glucose (FPG) ≥15.0 mmol/l during the pretreatment phase,<br>history of at least 1 severe hypoglycemia episode within 6 months before screening,<br>eGFR < 55 ml/min/1.73 m2 (or < 60 ml/min/1.73 m^2 based upon restriction of metformin use in the local label) or serum creatinine ≥124 µmol/l for men and ≥115 µmol/l for women,<br>uncontrolled hypertension (systolic BP ≥160 mmHg or diastolic BP ≥100 mmHg),<br>taking any ant hyperglycemic agent other than metformin plus

	sulphonylurea within 12 weeks prior to screening.
**Notes**	ClinicalTrials.gov identifier: NCT01106625<br>In this review, data from the 26 week 'core' period and the extension period (52 weeks) contributed data to our analyses.

Risk of bias table

**Bias**	**Authors' judgement**	**Support for judgement**
Random sequence generation (selection bias)	Low risk	Randomization was performed using an Interactive Voice Response System/Interactive Web Response System based on a computer-generated schedule prepared by the sponsor before the study.
Allocation concealment (selection bias)	Unclear risk	Randomization was performed using an Interactive Voice Response System/Interactive Web Response System based on a computer-generated schedule.
Blinding of participants and personnel (performance bias)	Low risk	To maintain blinding after randomization, HbA1c and FPG values were masked to study centers unless these values met prespecified glycemic rescue criteria or after glycemic rescue therapy was started. Patients, investigators and local sponsor personnel remained blinded throughout the core study and extension period.
Blinding of outcome assessment (detection bias)	Unclear risk	After completion of the core treatment period, the database was locked and the study was unblinded by the sponsor for regulatory filing; patients, investigators and local sponsor personnel remained blinded throughout the extension period.
Incomplete outcome data (attrition bias)	Low risk	264/469 randomized completed i.e. took at least 1 dose of double- blind study drug. Primary efficacy analyses were conducted using the modified intent-to-treat (mITT) population (all randomized patients who took 1 dose of double- blind study drug. Efficacy data were analyzed according to randomized treatment with the last observation carried forward (LOCF) approach used to impute missing values.
Selective reporting	Low risk	Major clinically relevant outcomes reported.

(reporting bias)		
Other bias	Unclear risk	This study was supported by Janssen Research & Development, LLC who provided assistance in clinical review of patient safety and adherence to the study protocol and contributions to the analysis and interpretation of study data and editorial support.

Footnotes

eGFR: estimated glomerular filtration rate

T2DM: Type 2 diabetes mellitus

T1DM : type 1 diabetes mellitus

BMI: body mass index

T2DM: type 2 diabetes mellitus

HbA1c: glycated hemoglobin

OAD: oral antidiabetic

SU: sulphonylurea
