# Supplementary material for: Benefits and Harms of Sodium-Glucose Co-Transporter 2 Inhibitors in Patients with Type 2 Diabetes: A Systematic Review and Meta-Analysis
Source: PLoS One. 2016 Nov 11;11(11):e0166125. doi: 10.1371/journal.pone.0166125 (PMC5106000; doi:10.1371/journal.pone.0166125)
Supplement: S2 Table — (PDF) [file pone.0166125.s007.pdf]

S2 Table. Primary outcome effect sizes, all comparisons.

Outcome	Effect Measure	Total n	Effect Estimate	CI Start	CI End	I^2(Q)%
**Efficacy of SGLT2-i versus placebo: primary outcomes**						
Primary outcome: change in HbA1c	MD	9154	-0.69	-0.75	-0.62	74.6
Serious adverse events	RR	11140	0.99	0.88	1.13	0.0
Mortality	RR	9780	1.36	0.69	2.66	0.0
Severe hypoglycemia	RR	5083	0.75	0.23	2.43	0.0
All cancers	RR	6453	1.04	0.60	1.83	3.2
Bladder cancer	RR	5244	0.33	0.01	8.18	n/a
Breast cancer	RR	5558	1.73	0.56	5.36	0.0
CVD	RR	10746	1.24	0.86	1.81	0.0
**SGLT2-i versus metformin: primary outcomes**						
Primary outcomes: change in HbA1c	MD	522	-0.05	-0.21	0.12	0.0
Dapagliflozin 10mg	MD	522	-0.05	-0.21	0.12	0.0
Serious adverse events	RR	535	1.19	0.37	3.85	0.0
Dapagliflozin 10mg	RR	535	1.19	0.37	3.85	0.0
Mortality	RR	531	0.32	0.01	7.73	n/a
Dapagliflozin 10mg	RR	531	0.32	0.01	7.73	n/a
All cancers	RR	103	0.00	0.00	0.00	n/a
Dapagliflozin 10mg	RR	103	0.00	0.00	0.00	n/a
Bladder cancer	RR	530	0.00	0.00	0.00	n/a
Dapagliflozin 10mg	RR	530	0.00	0.00	0.00	n/a
Breast cancer	RR	530	0.00	0.00	0.00	n/a
Dapagliflozin 10mg	RR	530	0.00	0.00	0.00	n/a
**SGLT2-i versus sulfonylureas (glipizide or glimepiride): primary outcomes**						
Primary outcome: change in HbA1c	MD	2662	-0.15	-0.21	-0.08	0.0
Canagliflozin 300mg vs. glimepiride (8mg)	MD	967	-0.19	-0.29	-0.09	n/a
Dapagliflozin 10mg vs. glipizide (20mg)	MD	150	-0.30	-0.79	0.19	n/a
Empagliflozin 25mg vs. glimepiride (1 to 4 mg)	MD	1545	-0.11	-0.19	-0.03	n/a

Serious adverse events	RR	3565	0.96	0.65	1.42	81.2
Canagliflozin 300mg vs. glimepiride (8mg)	RR	1036	0.68	0.48	0.96	n/a
Dapagliflozin 10mg vs. glimepiride (8mg)	RR	895	0.93	0.70	1.23	n/a
Empagliflozin 25mg vs. glimepiride (8mg)	RR	1634	1.36	1.06	1.76	n/a
Mortality	RR	3338	0.86	0.36	2.04	0.0
Canagliflozin 300mg vs. glimepiride (8mg)	RR	969	1.49	0.25	8.88	n/a
Dapagliflozin 10mg vs. glimepiride (8mg)	RR	819	0.40	0.08	2.06	n/a
Empagliflozin 25mg vs. glimepiride (8mg)	RR	1550	1.02	0.30	3.51	n/a
Severe hypoglycemia	RR	1800	0.13	0.02	0.73	21.5
Canagliflozin 300 mg vs glimepiride	RR	983	0.06	0.01	0.47	n/a
Dapagliflozin 10mg vs. glimepiride (8mg)	RR	817	0.33	0.03	3.21	n/a
CVD	RR	2518	0.88	0.28	2.79	0.0
Canagliflozin 300mg vs. glimepiride (8mg)	RR	968	0.33	0.01	8.11	n/a
Empagliflozin 25mg vs. glimepiride (8mg)	RR	1550	1.02	0.30	3.51	n/a
**SGLT2-i versus DPP-4i (saxagliptin, sitagliptin, linagliptin): primary outcomes**						
Primary outcome: change in HbA1c	MD	2834	-0.25	-0.36	-0.14	72.5
Canagliflozin 300mg vs. sitagliptin 100mg	MD	1605	-0.22	-0.40	-0.05	75.0
Dapagliflozin 10mg vs. saxagliptin 5mg	MD	294	-0.32	-0.53	-0.11	n/a
Empagliflozin 25mg vs. linagliptin 5mg	MD	534	-0.33	-0.66	0.00	87.7
Empagliflozin 25mg vs. sitagliptin 100mg	MD	401	-0.12	-0.27	0.03	n/a
Serious adverse events	RR	2954	1.09	0.73	1.63	7.3
Canagliflozin 300mg vs. sitagliptin 100mg	RR	1593	1.09	0.67	1.77	0.0
Dapagliflozin 10mg vs. saxagliptin 5mg	RR	361	0.33	0.07	1.60	n/a
Empagliflozin 25mg vs. sitagliptin 100mg	RR	452	0.83	0.26	2.69	n/a
Empagliflozin 25mg vs. linagliptin 5mg	RR	548	1.98	0.52	7.48	58.1
Mortality	RR	2774	4.07	0.69	24.11	0.0
Canagliflozin 300mg vs. sitagliptin 100mg	RR	1435	3.65	0.40	32.92	0.0
Dapagliflozin 10mg vs. saxagliptin 5mg	RR	355	0.00	0.00	0.00	n/a
Empagliflozin 25mg vs. sitagliptin 100mg	RR	446	0.00	0.00	0.00	n/a
Empagliflozin 25mg vs. linagliptin 5mg	RR	538	5.00	0.24	103.18	n/a
Severe hypoglycemia	RR	2039	1.21	0.60	2.47	0.0
Canagliflozin 300mg vs. sitagliptin 100mg	RR	1501	1.21	0.60	2.47	0.0
Empagliflozin 25mg vs. linagliptin 5mg	RR	538	0.00	0.00	0.00	n/a

All cancers	RR	2292	2.41	0.69	8.37	0.0
Canagliflozin 300mg vs. sitagliptin 100mg	RR	1491	2.31	0.60	8.95	0.0
Dapagliflozin 10mg vs. saxagliptin 5mg	RR	355	0.00	0.00	0.00	n/a
Empagliflozin 25mg vs. sitagliptin 100mg	RR	446	3.00	0.12	73.25	n/a
Bladder cancer	RR	2289	0.00	0.00	0.00	n/a
Canagliflozin 300mg vs. sitagliptin 100mg	RR	1488	0.00	0.00	0.00	n/a
Dapagliflozin 10mg vs. saxagliptin 5mg	RR	355	0.00	0.00	0.00	n/a
Empagliflozin 25mg vs. sitagliptin 100mg	RR	446	0.00	0.00	0.00	n/a
Breast cancer	RR	2289	2.99	0.12	73.20	n/a
Canagliflozin 300mg vs. sitagliptin 100mg	RR	1488	2.99	0.12	73.20	n/a
Dapagliflozin 10mg vs. saxagliptin 5mg	RR	355	0.00	0.00	0.00	n/a
Empagliflozin 25mg vs. sitagliptin 100mg	RR	446	0.00	0.00	0.00	n/a
