# Supplementary material for: Benefits and Harms of Sodium-Glucose Co-Transporter 2 Inhibitors in Patients with Type 2 Diabetes: A Systematic Review and Meta-Analysis
Source: PLoS One. 2016 Nov 11;11(11):e0166125. doi: 10.1371/journal.pone.0166125 (PMC5106000; doi:10.1371/journal.pone.0166125)
Supplement: S3 Table — (PDF) [file pone.0166125.s008.pdf]

S3 Table. Characteristics of excluded studies

Araki 2015

**Reason for exclusion**	RCT(empagliflozin) (NCT01368081). No blinded control group.

Bailey 2012

**Reason for exclusion**	RCT (dapagliflozin) (NCT 00528372), but not 10 mg, low doses used, outside our inclusion criteria for doses of at least 10 mg dapagliflozin.

Bailey 2015

**Reason for exclusion**	RCT (dapagliflozin) (NCT 00528372) but not 10mg. Low dose (2.5 mg) versus placebo. Previous inadequate control on diet and exercise, randomised trial of Dapagliflozin 10mg 2.5mg versus placebo then 500mg metformin given to placebo group at week 24.

Barnett 2014

**Reason for exclusion**	RCT (empagliflozin) (NCT01164501), participants had chronic kidney disease and were organised into groups based on the severity of their kidney disease prior to randomisation.

Ferrannini 2013b

**Reason for exclusion**	78-week extension of Ferrannini 2013, (empagliflozin) open, placebo switched to active (NCT00881530).

Fulcher 2015

**Reason for exclusion**	RCT (canagliflozin) (NCT01032629), 'CANVAS' study. Outcome data not reported for both intervention group and control group.

Inagaki 2014

**Reason for exclusion**	RCT(canagliflozin) (NCT01022112), low doses used, outside our inclusion criteria for doses of at least 300 mg of canagliflozin.

Kadowaki 2015

**Reason for exclusion**	Extension of Kadowaki 2014 (empagliflozin). In the 40-week extension period patients on other than 10 mg and 25 mg empagliflozin, were reallocated to empagliflozin 10 or 25 mg (NCT01193218).

Kohan 2013

**Reason for exclusion**	RCT (dapagliflozin) (NCT00663260) performed in patients with kidney disease, outside our inclusion criteria of normal kidney function.

Ljunggren 2012

**Reason for exclusion**	Additional report of Bolinder 2012 (NCT00855166), (dapagliflozin) reports safety outcomes including serum markers of bone formation (procollagen type 1 N-terminal propeptide; P1NP) and resorption (C-terminal cross-linking telopeptides of type I collagen; CTX), bone mineral density (BMD) as assessed by standardized Dual-Energy X-ray Absorptiometry (DXA) measurements and adverse events of fracture were evaluated as safety objectives.

Matthaei 2015a

**Reason for exclusion**	RCT (NCT01619059). Not intervention versus placebo or intervention versus OAD. Compares add on therapy (saxagliptin) to dapagliflozin. Saxagliptin add on to metformin and dapagliflozin versus placebo add on to metformin and dapagliflozin.

NCT01294423

**Reason for exclusion**	Unpublished data at time of search in September 2015

Neal 2015

**Reason for exclusion**	RCT (canagliflozin)'CANVAS' study (NCT01032629). No outcome data available for intervention and control group. ("Efficacy end points of a continuous nature were assessed using an ANCOVA model, with treatment and stratification factor (i.e., background glucose-lowering therapy at screening) as fixed effects, and the corresponding baseline value as a covariate. Least squares mean differences between treatment groups and the associated

	two-sided 95% CIs were estimated based on this model.")

Schumm 2015

**Reason for exclusion**	Unpublished data at time of search in September 2015.

Stenløf 2014

**Reason for exclusion**	RCT (canagliflozin). Extension of Stenløf 2013, open, placebo switched to active (NCT01081834).

Wilding 2014

**Reason for exclusion**	Extension of Wilding 2012 (dapagliflozin) with additional 24 and 56 week data but at 49 weeks, participants switch from 5mg to 10mg of dapagliflozin (NCT00673231).

Yale 2013

**Reason for exclusion**	RCT (canagliflozin), performed in patients with kidney disease, outside our inclusion criteria of normal kidney function.

Footnotes

RCT: randomized controlled trial

See S1 Appendix.docx for data sources (Reference list of excluded studies).
